# Identification of Methylglyoxal Reactive Proteins with Photocaged Glycating Agents

**DOI:** 10.1002/cbic.202500275

**Published:** 2025-09-29

**Authors:** Saskia Sokoliova, I. Raluca Sardaru, Franciszek P. Warguła, Jos H. Hermans, Hjalmar P. Permentier, Peter L. Horvatovich, Martin D. Witte

**Affiliations:** ^1^ Stratingh Institute for Chemistry University of Groningen 9747 AG Groningen The Netherlands; ^2^ Department of Analytical Biochemistry Groningen Research Institute of Pharmacy University of Groningen 9700 AD Groningen The Netherlands

**Keywords:** chemical probes, chemoproteomics, glycation, photocage, protein profiling

## Abstract

Methylglyoxal is a highly reactive metabolite that is formed spontaneously in the glycolytic pathway. The side chains of various amino acid residues react with methylglyoxal to form advanced‐glycation end products (AGEs). This enzyme‐independent process introduces post‐translational modifications onto the proteins and it is long thought that the resulting AGEs primarily inhibit proteins. More recent studies have shown that these AGEs can act in signaling and feedback loops and that a large number of proteins react reversibly with methylglyoxal. These findings lead to a renewed interest in methylglyoxal‐induced AGEs and lead to the development of novel tools and methodologies that can be used to identify the modified proteins. Many of studies are nowadays still performed by adding methylglyoxal exogenously, often in a high concentration, despite the high reactivity of methylglyoxal. Herein, new photocaged‐methylglyoxal derivatives are reported that allow the direct release of methylglyoxal in the sample of interest by irradiating the photocaged probe with UV light. It is shown that this labeling approach is more efficient. A far larger number of proteins are labeled with the photocaged probes than with the chemically activated probes. The here reported approach should allow studying in situ glycation under physiological more relevant conditions.

## Graphical abstract

Photocaged methylglyoxal (PC‐MGO) derivatives enable studying protein glycation under mild conditions. The active glycating agents can be released in the protein solution by irradiating the sample with UV light. Compared to the earlier reported chemically activated MGO probes, the photocaged probes are more potent. The light triggered release of MGO probes will open opportunities to study glycation with a higher cellular precision.

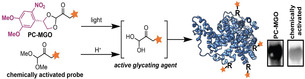

## 1 Introduction

**Figure 1:**
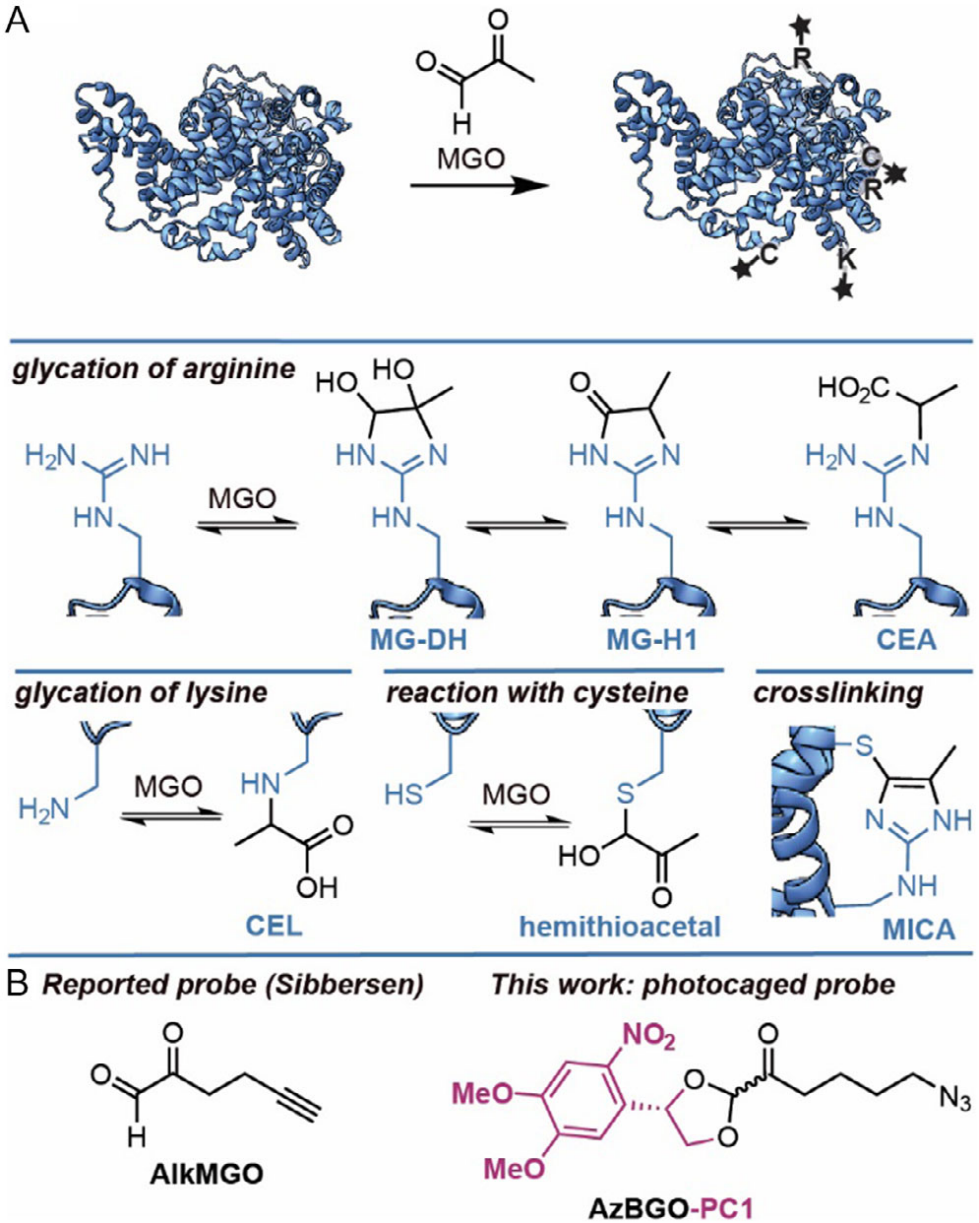
Schematic representation of the glycation process. A) Amino acid residues react with MGO yielding glycated proteins. The adducts formed between MGO and arginine, cysteine, and lysine residues are depicted. B) Molecular structure of the probe developed by Sibbersen et al. and the here reported photocaged probe. Image of the protein structure was generated from PDB: 4F5S with ChimeraX and was further modified in Adobe Illustrator.

Cells produce a large variety of metabolites. It has been known for decades that a subset of these metabolites modify proteins in an enzyme‐independent manner. The post‐translational modifications (PTMs) resulting from these reactions alter the protein's structure and as a result can alter the protein's interaction with other (bio)molecules, the protein's activity, and the protein's stability.^[^ ^1^ ^,^ ^2^ ^]^ A notable example of a metabolite that reacts with proteins is methylglyoxal (MGO) (**Figure** **1**A).^[^ ^3^ ^]^ This highly reactive α‐oxoaldehyde metabolite is spontaneously formed in the glycolytic pathway from triosephosphates.^[^ 4, 5, 6, 7, 8, 9 ^]^ MGO reacts reversibly with the guanidinium group in arginine residues to form MGO‐derived dihydroxyimidazolidines (MG‐DH). Dehydration of MG‐DH slowly converts the initial adducts into MGO‐derived hydroimidazolines (MG‐H1−3), which are more stable. Three regioisomers of MG‐H are formed, of which MG‐H1 is the most abundant (depicted in Figure 1A).^[^ ^4^ ^,^ ^5^ ^]^ Hydrolysis of MG‐H can eventually lead to the formation of carboxyethyl arginine (CEA). The ε‐amino group of lysine residues reacts with MGO as well. The keto‐group forms a Schiff base with the amine and this adduct can subsequently tautomerize into carboxyethyl lysine (CEL) (Figure 1A).^[^ ^6^ ^]^ Finally, cysteine residues have been shown to react with MGO to form hemithioacetals.^[^ ^7^ ^]^ Recent work by Moellering and co‐workers demonstrated that the hemithioacetals react further with guanidinium groups of arginine, resulting in the formation of mercaptomethylimidazole cross‐links between arginine residues and cysteine residues (Figure 1A).^[^ ^8^ ^,^ ^9^ ^]^ These combined processes, together with several other modifications, are known as protein glycation and the products are referred to as advanced‐glycation end products.

MGO is cytotoxic because of its high reactivity, and the formed glycation products contribute to further aggravating cellular damage.^[^ ^10^ ^]^ Under normal physiological conditions, MGO is rapidly converted into less reactive metabolites by enzymatic detoxification mechanisms, which minimizes cellular damage. The most important pathway for detoxification is the cytosolic glyoxalase system, which employs glutathione to tautomerize α‐oxoaldehydes into α‐hydroxy acids.^[^ ^3^ ^,^ ^11^ ^]^

It was long thought that the glycation adducts merely inhibited the natural functions of proteins. The discovery that reactive metabolites may act in regulatory feedback loops led to a renewed interest in nonenzymatic PTM of proteins in general,^[^ 12, 13, 14 ^]^ and glycation in specific,^[^ ^8^ ^,^ ^9^ ^,^ 15, 16, 17, 18 ^]^ and led to the development of novel tools and strategies to identify the proteins that react with MGO, the so‐called dicarbonyl proteome. Global proteomic studies with MGO have been performed to identify glycated proteins (for a review, see ref. [19]). Glycation of isolated proteins and peptide libraries followed by mass‐spectrometry analysis has been used to determine preferential modification sites in a systematic manner.^[^ ^20^ ^,^ ^21^ ^]^ Recently, a quantitative proteomic strategy was developed to identify proteins that are cross‐linked by MGO.^[^ ^18^ ^]^

A challenge in identifying MGO‐derived PTMs with proteomics is that MGO forms structurally diverse PTMs for which antibodies are not always available and may have variable specificity. Moreover, some of these PTMs have a limited stability.^[^ ^22^ ^]^ Chemoproteomic approaches and chemical probes have been developed that overcome some of these limitations. Moellering employed competitive reactive cysteine profiling with iodoacetamide alkyne (IA‐alkyne) to identify cysteine residues that react reversibly with MGO.^[^ ^23^ ^]^ Sibbersen et al. reported an alkyne‐functionalized MGO derivative, AlkMGO (Figure 1B), for the direct identification of stable MGO‐derived PTMs.^[^ ^15^ ^]^ The bioorthogonal tag in AlkMGO simplified the enrichment and identification of glycated proteins in red blood cells and plasma.^[^ ^16^ ^]^ A homolog of AlkMGO was used by David and co‐workers to study histone glycation.^[^ ^24^ ^]^ Johannsen and co‐workers demonstrated that the glyoxalase system in cells metabolizes AlkMGO into lactoylglutathione.^[^ ^16^ ^,^ ^25^ ^]^ The increased levels of lactoylglutathione induce enzyme‐independent lactoylation of lysine residues and the proteins that are susceptible to lactoylation were readily identified using AlkMGO.^[^ ^26^ ^]^

The reported chemical probes have several limitations that hamper their application. The high reactivity of MGO derivatives reduces the shelf life of the reported probes considerably. Consequently, the aldehyde group in the probes has to be stored as the corresponding dimethyl acetal (DMA) form. This protecting group must be hydrolyzed by heating the probe in 5% aqueous sulfuric acid prior to the labeling reaction.^[^ ^15^ ^,^ ^16^ ^]^ The harsh conditions used to hydrolyze the dimethyl acetal are incompatible with proteins and therefore, the α‐oxoaldehyde probes cannot be generated directly in the presence of proteins. Moreover, often relatively high concentrations of the probe are used to detect glycated protein forms. In combination with the high reactivity of dicarbonyl compounds, these approaches lead to artifacts and off‐target labeling.

We hypothesized that off‐target labeling may be circumvented by synthesizing photocaged derivatives of the MGO probes. In these reagents, the reactive group is masked with the photocage and the compound only becomes active after the removal of the photocage by irradiating the sample with light of the appropriate wavelength, when the probe penetrated the sample and reached the site of action. This approach is commonly used in photopharmacology,^[^ ^27^ ^]^ but it has also been used to mask cysteine reactive probes,^[^ ^28^ ^]^ and recently by Hurben et al. to develop a targetable MGO precursor.^[^ ^29^ ^]^ In the presented study, we report the synthesis and biological study of a series of nitroaryl‐caged MGO probes (AzBGO‐PC1, AlkMGO‐PC1, AlkMGO‐PC2) that get activated upon irradiation (365 nm) and release MGO species directly into the biological sample of interest.

## 2 Results and Discussion

**Figure 2:**
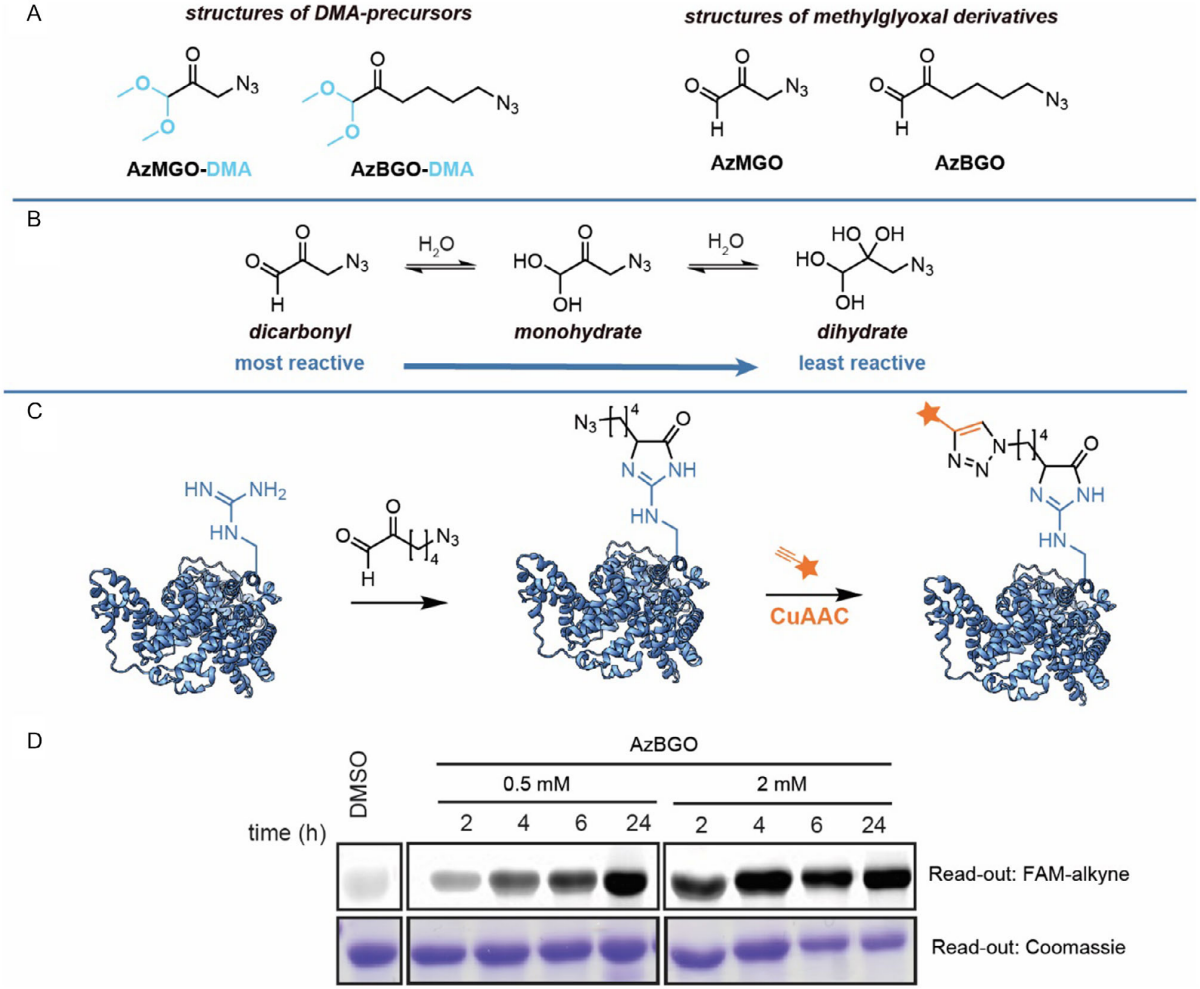
A) Structures of the AzMGO and AzBGO and the corresponding dimethyl acetal precursors AzMGO‐DMA and AzBGO‐DMA. B) Hydrate formation by the probe. C) Schematic representation of the labeling with AzBGO. D) In‐gel fluorescence read‐out of BSA labeling with AzBGO. The probe was prepared by hydrolyzing AzBGO‐DMA with 5% H_2_SO_4_ at 100 °C for 30 min prior to the labeling step. The crude probe mixture was neutralized with potassium hydroxide to pH 7. BSA (1 mg mL^−1^) in PBS (pH 7.4) was incubated with the probe for the indicated time. The incubation with the probe was followed by a CuAAC reaction with 5‐FAM alkyne for 2 h at RT to visualize the labeled proteins. FAM‐alkyne was detected by in‐gel fluorescence scanning on a Typhoon FLA 9500. Image of the protein structure was generated from PDB: 4F5S with ChimeraX and was further modified in Adobe Illustrator. For uncropped images, see Figure S1, Supporting Information.

The reported MGO probes feature an alkyne handle for read‐out purposes.^[^ ^15^ ^,^ ^24^ ^]^ A one or two methylene spacer separates the bio‐orthogonal handle from the α‐oxoaldehyde reactive group. Molecules that have similar structural motifs have been shown to isomerize in the presence of transition metals,^[^ ^30^ ^]^ acids,^[^ ^31^ ^]^ and bases into the conjugated alkenes,^[^ ^32^ ^,^ ^33^ ^]^ in particular when the compound contains a single methylene unit. Isomerization of the alkyne would, in the case of these probes, lead to loss of the bio‐orthogonal handle and thus results in less efficient detection of the adducts. Moreover, it can complicate the synthesis of photocaged derivatives (see Scheme S4, Supporting Information). Even though the alkyne‐functionalized MGO probes were successfully used to study glycation, we first focused our attention on azide‐functionalized probes (**Figure** **2**A). Initially, we intended to prepare azidomethylglyoxal (AzMGO). This probe is comparable in size to MGO since the bio‐orthogonal handle is directly placed on the methyl group. However, attempts to prepare the dimethyl acetal of AzMGO were not successful. Bromination of MGO 1,1‐dimethyl acetal followed by a substitution reaction with sodium azide gave a complex mixture, presumably caused by the limited stability of the bromomethylglyoxal intermediate and potentially also the product.^[^ ^34^ ^]^

A spacer was introduced between the bio‐orthogonal handle and the α‐oxoaldehyde reactive group to increase the stability of the probe. While the resulting AzBGO has a larger molecular footprint than MGO, we reason that the reactivity of AzBGO is more comparable to MGO since the spacer reduces the electron withdrawing effect of the azido group on the reactive group. Consequently, the handle will have a less pronounced effect on hydrate formation and thereby on the reactivity of the probe (Figure 2B). Starting from 6‐bromo‐1‐hexene, the dimethyl acetal precursor of azidobutylglyoxal (AzBGO‐DMA) was synthesized in five synthetic steps (Scheme S2, Supporting Information). The dimethyl acetal precursor was prepared to increase the shelf life of the compound (Figure 2A). The final synthetic step—hydrolysis of the dimethyl acetal with aqueous sulfuric acid—was performed prior to the labeling experiments. The crude product of the hydrolysis reaction was, after neutralization and dilution with DMSO, directly used to assess AzBGO's glycation efficiency. Various studies have shown that plasma proteins are particularly susceptible to glycation,^[^ ^16^ ^,^ ^35^ ^]^ and therefore, we selected bovine serum albumin (BSA) as a model protein to assess if AzBGO is bona fide glycating agent. Covalent modification of BSA by AzBGO was detected via the azido group. A copper‐catalyzed azide–alkyne click (CuAAC) reaction with fluorescein‐5‐carboxamido alkyne (5‐FAM alkyne) allowed visualization of the labeled proteins by scanning the in‐gel fluorescence (Figure 2C). In the DMSO control samples, some labeling of BSA was observed, presumable resulting from nonspecific interactions between the fluorophore and the protein, and/or from side reactions in the click reactions resulting in an increased background fluorescence.^[^ ^36^ ^]^ Strong fluorescent labeling of BSA was observed in samples that were treated with AzBGO (Figure 2D). However, the signal intensities in the samples that were treated with 2 mM of AzBGO reached saturation more rapidly. Efficient labeling of BSA was already achieved when the protein was incubated with 2 mM of AzBGO at 37 °C for 2 h. Longer incubation times led to a marginal increase of the labeling intensity (Figure 2D). AzBGO was less efficient at a lower concentration (0.5 mM) and a similar labeling intensity was only reached after 24 h.

**Figure 3:**
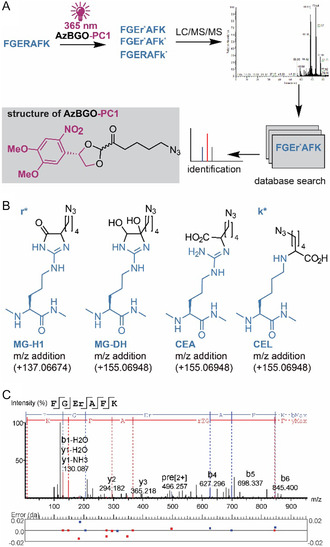
Labeling of the model peptide with the photocaged glycating agent AzBGO‐PC1. A) Schematic representation of the workflow and structure of AzBGO‐PC1. The peptide (sequence: FGERAFK; colored blue) are potentially modified at Lys and Arg residues (modified residues are written as k* and r*). The photocage in AzBGO‐PC1 is depicted in magenta. B) Molecular structures of the expected modifications on arginine and lysine after glycation with AzBGO‐PC1 including the expected mass shifts. The amino acids are depicted in blue, the PTMS in black. C) A MS/MS spectrum of the modified peptide (MG‐H, +137.06674 Da) at the arginine residue identified by PEAKS Xpro with the highest score (–10logP = 35.87).

Our next goal was to design a probe that allows light‐mediated release of AzBGO. The probe had to have a low intrinsic reactivity to proteins to circumvent labeling in the absence of light. Inspired by the work of Abo et al. on photocaged cysteine labeling reagents,^[^ ^28^ ^]^ we prepared the photocaged reagent AzBGO‐PC1 (**Figure** **3**A, for synthetic route see Scheme S3 and S4, Supporting Information), as a mixture of *cis‐trans* isomers. We opted to protect the aldehyde group with a nitroaryl photocage (PC1) since α‐oxoacetal compounds are considerably less reactive than the corresponding dicarbonyl compounds. Removal of the photocage with UV irradiation should liberate the active glycating agent AzBGO and thereby trigger the labeling event. First, we assessed if AzBGO‐PC1 could be uncaged. The probe dissolved in HEPES buffer was irradiated with a handheld UV lamp (365 nm). The probe has an absorption maximum at 360 nm which is characteristic for nitroaryl photocages. Upon irradiation, the peak at 360 nm gradually disappeared and complete uncaging was observed after 30 min (Figure S2, Supporting Information). A model reaction on a synthetic peptide with the sequence FGERAFK was performed to assess if irradiating AzBGO‐PC1 with UV light indeed led to glycation (Figure 3A). The selected amino acid sequence derived from human albumin and the arginine residue within this sequence is subject to glycation.^[^ ^5^ ^,^ ^35^ ^,^ ^37^ ^]^ Therefore, we reasoned that glycation of the model peptide by AzBGO‐PC1 would provide a conclusive proof that AzBGO is released upon exposure of the probe to UV light. Besides the formation of the reported MG‐H on the arginine residue (Figure 3B), three additional modifications might potentially be detected as well during the model reaction, namely MG‐DH, CEA, and CEL (Figure 3B). To detect the potential glycation products, we submitted a sample of the reaction to LC–MS/MS analysis. The data from the LC–MS/MS were analyzed by interpreting raw MS/MS spectra manually (Figure S3, Supporting Information) and using a database search with custom design PTMs for the above‐described modification in PEAKSs studio Xpro (Figure 3).^[^ ^38^ ^]^

In the LC–MS/MS data of the peptides labeled with AzBGO‐PC1, we primarily found the molecular ion [M + H]^+^ and the double charged [M + 2H]^2+^ that correspond to a peptide carrying an arginine MG‐H modification, which confirms that in vitro glycation took place. We selected the double charged ion [M + 2H]^2+^ as precursor ion and analyzed its corresponding MS/MS spectrum (Figure S3, Supporting Information). The MS/MS spectrum contained several fragments with the mass shift for the modification MG‐H (+137.06674 Da at the Arg) and CEA or MG‐DH (+155.06948 Da at the Arg), but also arginine‐containing fragments without modification were observed. This suggests that exposure of the MG‐H derivate to high energy fragmentation can lead to the ring opening, i.e., formation of CEA, and concomitant neutral loss of the carboxyethyl group. These observations are in line with earlier work by Schmidt et al. and this information is especially important for the analysis of more complex biological samples as well as enrichment of the peptides with the modification from the complex mixtures.^[^ ^37^ ^]^

In the PEAKS analysis, the nonmodified peptide (data not shown), as well as peptides with a PTM of either +137.06674 Da (Figure 3C) or + 155.06948 Da PTM (Figure S4, Supporting Information) were identified. The PTMs are located at the Arg residue according to the MS/MS spectra, suggesting that both MG‐H and MG‐DH or CEA‐functionalized peptides are formed during the glycation process, respectively. This finding is further supported by the observation that the modified peptides eluted differently. The intensity of the molecular ion of the MG‐H peptide is three orders of a magnitude higher than that of MG‐DH or CEA‐functionalized peptide, confirming that MG‐H was the major PTM formed under these conditions. This corroborates the results of Schmidt et al.^[^ ^37^ ^]^

**Figure 4:**
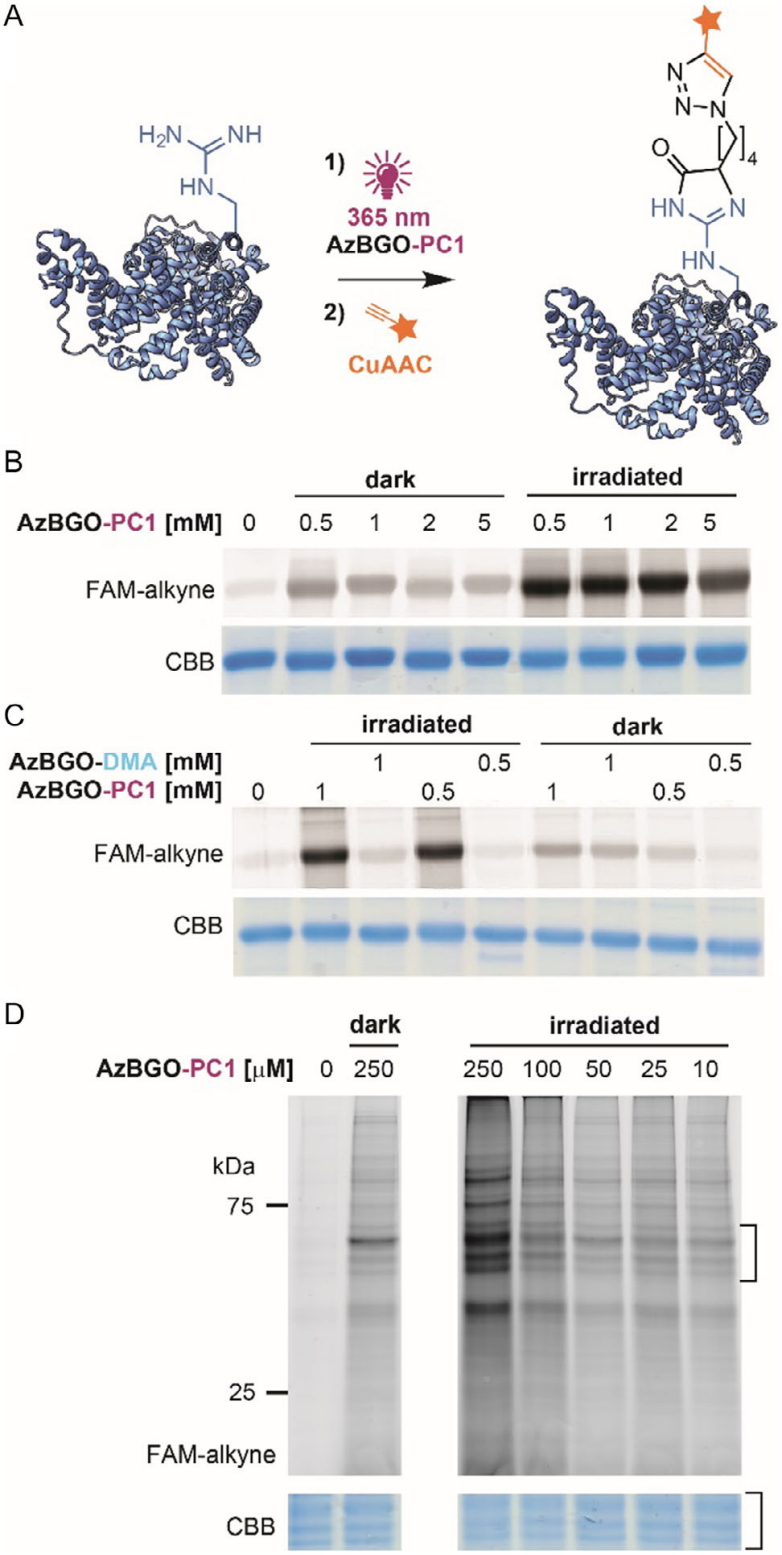
Application of AzBGO‐PC1 on BSA and A549 cell lysates. A) Schematic representation of the reaction workflow, where the AzBGO‐PC1 is applied directly to the protein and AzBGO is released upon irradiation in situ. B) In‐gel fluorescence read‐out of the BSA labeling with AzBGO‐PC1 after incubating in the dark or exposure to 365 nm light. The probe‐labeled proteins were visualized by CuAAC with 5‐FAM‐alkyne. C) Comparison of the probes AzBGO‐PC1 and chemically prepared AzBGO demonstrates the efficiency of photouncaging. D) Labeling of A549 cell lysate with the AzBGO‐PC1. SDS‐PAGE analysis showed that labeling of proteins becomes notable at 250 μM of AzBGO‐PC1. The labeled proteins were visualized by CuAAC with 5‐FAM‐alkyne. For uncropped gels, see Figure S5, Supporting Information.

Next, AzBGO‐PC1 was tested on whole proteins. Varying concentrations of AzBGO‐PC1 were added to BSA and the solution was left to incubate either under irradiation with UV light (365 nm) or in the dark for 15 min (**Figure** **4**). The uncaging‐glycation process was visualized via postlabeling functionalization of the glycated protein with a CuAAC reaction with 5‐FAM‐alkyne. Exposing the samples to UV light liberates the active glycating agent (AzBGO) and therefore glycation of BSA should be more pronounced in the samples that were irradiated compared to those that were incubated in the dark. As is apparent from the in‐gel fluorescence scan, the fluorescence intensity is indeed considerably stronger in the UV‐treated samples, again suggesting that the active alkylglyoxal probe—AzBGO—is liberated upon irradiation (Figure 4B). The fluorescence intensity already reached a plateau at the lowest concentration used. MGO has been reported to be a UVA‐photosensitizer,^[^ ^39^ ^]^ and therefore control experiments with AzBGO were performed (Figure 4C). The in‐gel fluorescence scan revealed that, in contrast to AzBGO‐PC1, the labeling of the proteins by AzBGO was not affected by UV light, thereby assuring that the observed labeling was induced by uncaging of AzBGO‐PC1 and not by a photochemical reaction between the AzBGO and the protein. Noticeable background labeling is observed in all samples that were not exposed to UV light, either resulting from proteins that react with the ketone functionality in AzBGO‐PC1, or from side‐reactions that occur during the click reaction.^[^ ^36^ ^]^ The background labeling can be reduced by removing the excess of probe before the click reaction (Figure S6, Supporting Information) and by changing the click handle.

Having shown that AzBGO‐PC1 labels whole proteins, we applied it on lysates of human A549 cells and *Escherichia coli* cells. The resulting solutions were incubated in the dark, or while irradiating with daylight or with a UV lamp. The protein‐probe adducts were visualized via a CuAAC reaction with 5‐FAM‐alkyne (Figure 4D). The results of the in‐gel fluorescence scan revealed that AzBGO‐PC1 labels numerous proteins in A549 cell lysate. The most intense signals were observed when the protein sample was incubated with 250 μM of AzBGO‐PC1 while being exposed to 365 nm wavelength UV light. Again, residual labeling was observed in dark conditions, either resulting from a background reaction with the keto group in the nonactivated probe or from side‐reactions with the reporter group in the CuAAC step.^[^ ^36^ ^]^ Yet the results suggest certain selectivity between the samples irradiated with 365 nm UV light and control conditions (Figure 4D). The signal‐to‐background labeling can be improved by removing the excess of the probe prior to the click reaction (Figure S6, Supporting Information) and by optimizing the click conditions (Figure S7, Supporting Information).

**Figure 5:**
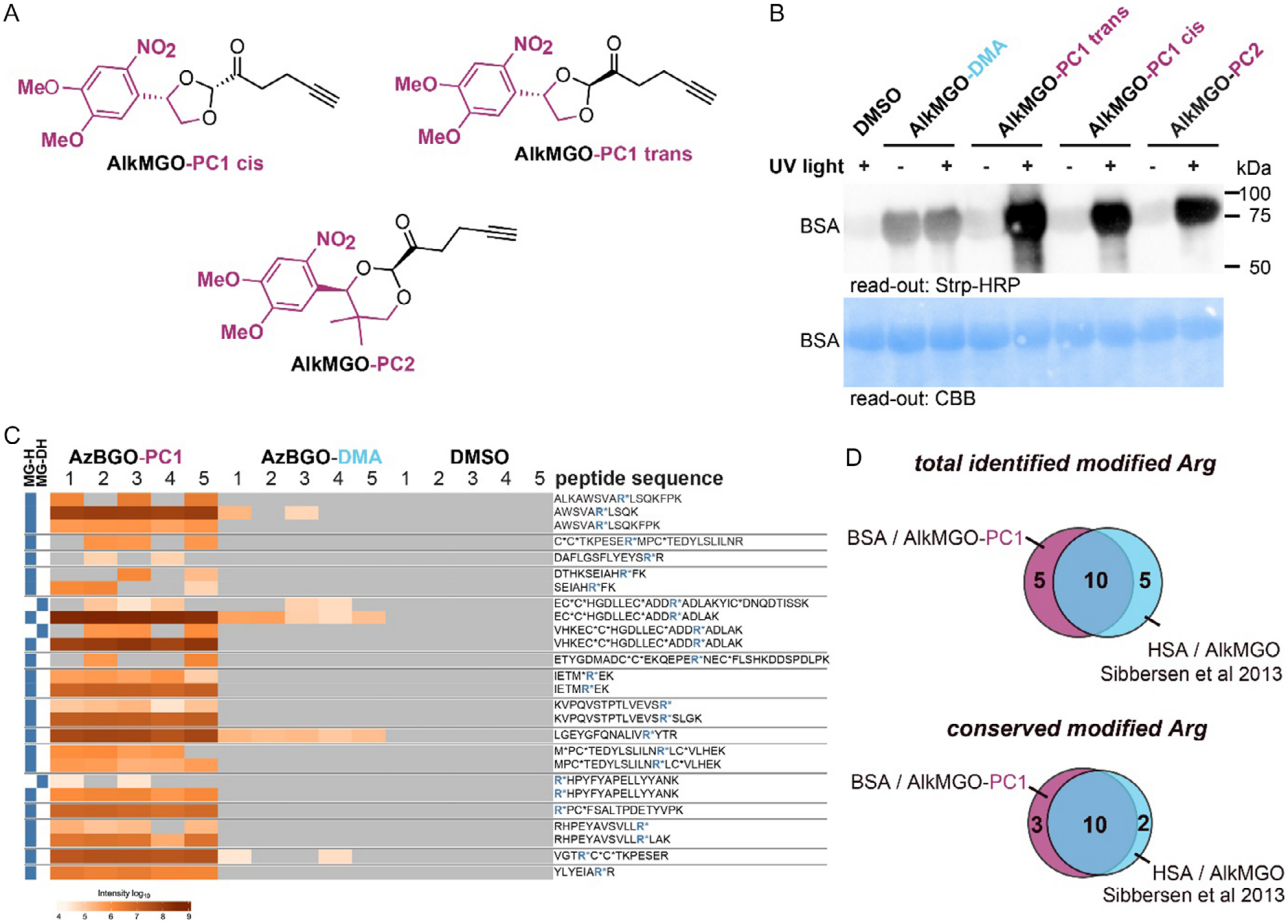
A) Structures of the photocaged‐AlkMGO derivatives. B) Western blot read‐out of the glycation of BSA with photocaged AlkMGO derivatives. Chemical‐activated AlkMGO was prepared by hydrolyzing AlkMGO‐DMA with 5% H_2_SO_4_ (H_2_O/DMSO, 1/1) at 100 °C for 30 min prior to the labeling step. The crude probe mixture was neutralized with potassium hydroxide to pH 7. BSA (1 mg mL^−1^, 90 μL) in PBS (pH 7.4) was incubated with the probe for 1 h. During this period, the samples were either irradiated with UV light (365 nm) or kept in the dark. The incubation with the probe was followed by a CuAAC reaction with biotin‐azide overnight at RT. After separation of the proteins on the SDS‐PAGE, the proteins were transferred to a PVDF membrane and the labeled proteins were visualized with Strp‐HRP and ECL+. For uncropped images, see Figure S7 C, Supporting Information. C) Heat map of the modified peptides identified in BSA that was labeled with AlkMGO‐PC1 trans (250 μM), AlkMGO‐DMA (250 μM, chemically activated prior to labeling) or DMSO for 1 h. D) Venn diagram of the comparison of the total modified residues and the conserved modified residues identified in this study compared to those identified in HSA by Sibbersen et al.

The positive results with AzBGO‐PC1 prompted us to synthesize three other derivatives AlkMGO‐PC1 (both the *trans* and *cis* derivative) and AlkMGO‐PC2 (see **Figure** **5**A), as it gives the option to use azide‐functionalized reporter groups, which might be useful for other experimental settings. AlkMGO‐PC2 contains a novel photocage inspired on the photocage reported by Bode and co‐workers.^[^ ^40^ ^]^ Moreover, these derivatives allowed us to establish how the photocaged probes behave in comparison with the previously reported probe AlkMGO. The uncaging efficiency of the AlkMGO‐PC1 derivatives is similar to AzBGO‐PC1. Uncaging of AlkMGO‐PC2 is slower and the UV–vis spectra revealed anomalous behavior. The absorption at 365 nm first increases before it decreases over time, suggesting that the uncaging mechanism for PC2 is different from PC1 (Figure S1, Supporting Information). Labeling experiments revealed that the *cis* and *trans* isomers of AlkMGO‐PC1, as well as AlkMGO‐PC2, showed labeling patterns that are similar to those obtained with AzBGO‐PC1 (Figure S6, Supporting Information). A direct comparison between the photocaged AlkMGOs and the chemically activated AlkMGO revealed a large difference in activity (Figure 5B, Figure S7, Supporting Information). Prominent labeling of BSA is observed when the samples are labeled with 250 μM of the photocaged AlkMGO, while chemically activated AlkMGO only gave weak labeling under the same conditions. Similar differences were observed for AzBGO‐PC1 and AzBGO‐DMA (Figure 4C). The reported hydrolysis conditions for the dimethyl acetal (100 mM in 5% aqueous H_2_SO_4_ at 100 °C for 30 min) led in our hands to accumulation of organic material on the walls of the Eppendorf tube. Accumulation of material could be suppressed by performing the acetal hydrolysis in a one‐to‐one mixture of DMSO and water, but only led to a marginal improvement of the labeling by the chemically activated AlkMGO (data not shown).

To assure that the photocaged AlkMGOs target the same residues as the chemically activated AlkMGO, we labeled BSA with AlkMGO‐PC1 or AlkMGO‐DMA for 1 h and identified the modified residues by mass spectrometry. In the samples that were treated with AlkMGO‐PC1, 15 out of the potential 23 arginine residues in the mature protein underwent glycation by the probe. The MG‐H levels highly varied (Figure 5C). Arginines 241, 280, 433, and 459 (full protein sequence numbering) were found to be most abundantly modified (Figure 5C, Figure S7, Supporting Information). These specific residues were also identified in the samples that were treated with the chemically activated AlkMGO, albeit not in all samples and with ion intensities that were orders of magnitudes lower than those for AlkMGO‐PC1. These results indicate that chemically activated AlkMGO and the photocaged AlkMGOs target the same residues in BSA, but that AlkMGO‐PC1 labels more efficient.

To further validate that the photocaged probes behave similar to AlkMGO, we compared our proteomic data with those reported for human serum albumin (HSA). BSA and HSA share 76% sequence identity and are highly similar in structure (Figure S8, Supporting Information). Glycation of HSA by AlkMGO and MGO has been studied by Sibbersen et al.^[^ ^15^ ^]^ Reacting HSA with AlkMGO or MGO overnight led to glycation of 15 arginine residues. There is a strong overlap between the datasets (Figure 5D), especially when comparing the conserved modified arginine residues (Figure 5D). In total, 15 of the identified modified residues are conserved and out of these residues 10 were identified in both studies. Three arginine residues were identified to be uniquely labeled in BSA with AlkMGO‐PC1 and the remaining two were uniquely identified in the study by Sibbersen et al. These findings further confirm that the photocaged probes react similar to the chemically activated probes, as well as MGO, but that they have the benefit of being more potent.

Next, we employed an activity‐based protein profiling multidimensional protein identification technology (ABPP‐MuDPIT)^[^ ^41^ ^]^ workflow to identify which proteins in human A549 cell lysate and *E. coli* cell lysate reacted with AzBGO‐PC1 and to determine the modification sites within these proteins. The samples were incubated with the photocaged probe AzBGO‐PC1 both with and without irradiation. As negative control, we used DMSO‐treated samples and as positive control the chemically activated probe AzBGO‐DMA. We removed the excess of the labeling reagents by protein precipitation, after which the glycated proteins were functionalized with a commercially available diazo‐biotin alkyne through a CuAAC reaction. Western blot analysis showed that the tag did not affect the labeling outcome (Figure S9, Supporting Information). The diazo linker can be cleaved with a sodium dithionite solution, which should facilitate the elution of the modified peptides from the beads. The excess of the click reagent was removed by a second protein precipitation. Enrichment of the biotinylated proteins with Neutravidin‐agarose pulldown was followed by on‐bead digestion with trypsin. The resulting peptides were eluted with 5% formic acid (FA) and measured on LC–MS/MS. The data of this first sample set were used to identify the proteins that reacted with the probe. The material that remained on the bead after the digestion should be enriched for the probe‐modified peptides. With the intention to identify the glycation site, we eluted these biotinylated peptides with sodium dithionite and measured this second sample set on LC–MS/MS as well.

**Figure 6:**
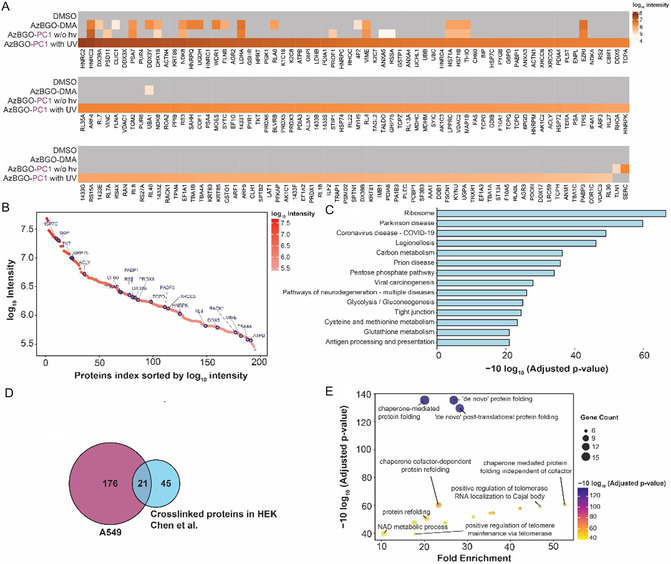
A) Heat map of proteins enriched from A549 cell lysate in the probe labeled samples over the DMSO control. Each condition was measured in triplicates and the average is presented in the heat map. B) Representation of the enriched proteins in A549 cell lysate sorted by the log_10_ intensity. C) Bar‐diagram of KEGG pathway analysis of the proteins identified in the AzBGO treated samples that were irradiated with UV light. D) Venn diagram of the comparison of the proteins enriched in this study compared to the proteins enriched by Chen et al.[18] E) Bubble chart of the ORA of proteins identified in the AzBGO treated samples. The proteins are clustered based on the biological process. The circle size shows the number of proteins in the enriched pathways.

**Figure 7:**
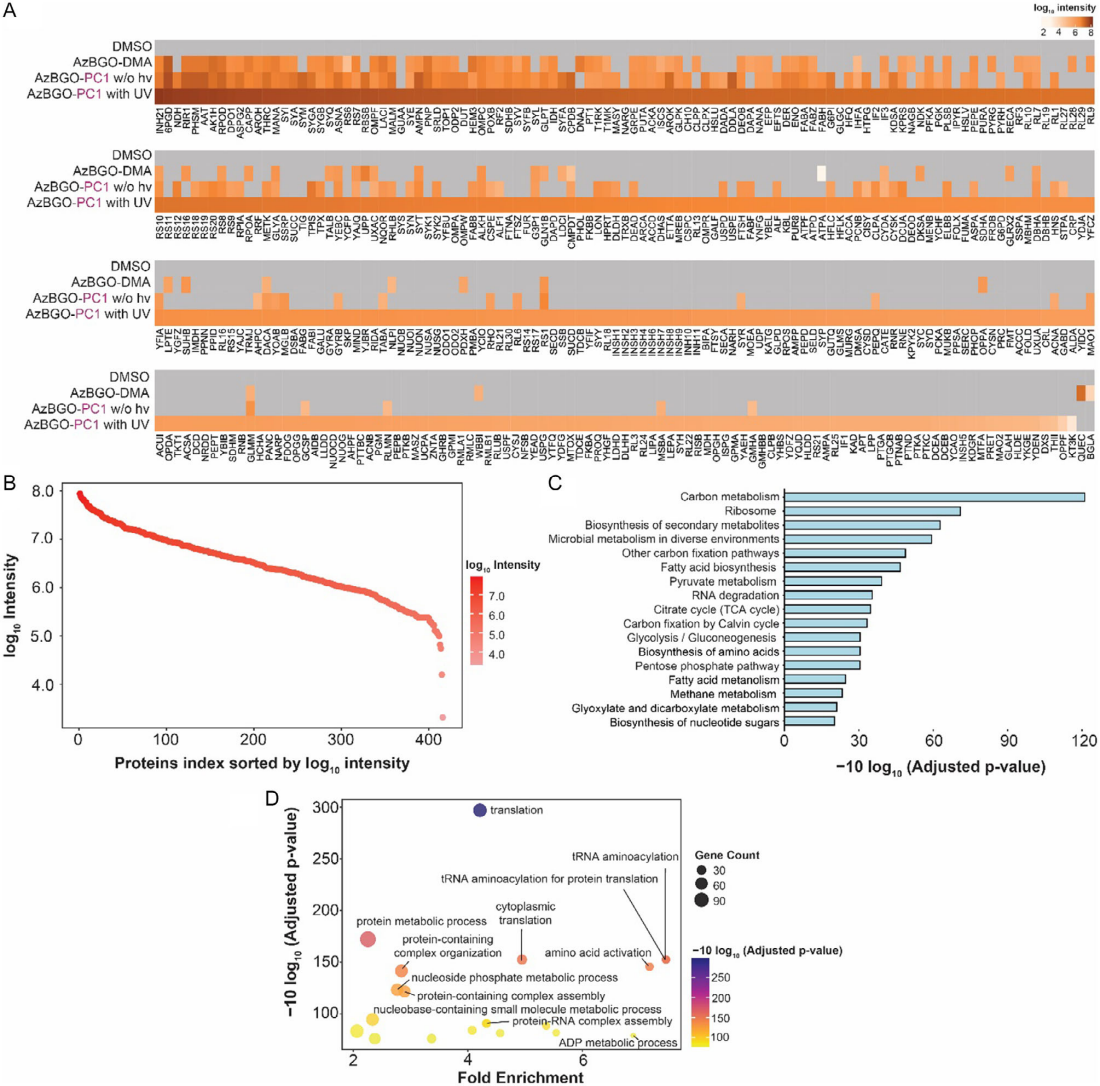
A) Heat map of proteins enriched from *E. coli* cell lysate in the probe labeled samples over the DMSO control. Each condition was measured in triplicates and the average is presented in the heat map. B) Representation of the enriched proteins in *E. coli* cell lysate sorted by the log_10_ intensity. C) Bar‐diagram of KEGG pathway analysis of the proteins identified in the AzBGO treated samples that were irradiated with UV light. D) Bubble chart of the ORA of proteins identified in the AzBGO treated samples. The proteins are clustered based on the biological process. The circle size shows the number of proteins in the enriched pathways.

The raw data of both sample sets were analyzed in the PEAKS search with MG‐H, MG‐DH, CEA, and CEL as expected modifications and with a custom‐made R script. A large number of proteins (>200 proteins in A549 cell lysate; >450 proteins in *E. coli* lysate) were identified in datasets that resulted from the on‐bead digestion. Of the identified proteins, the majority were uniquely identified in the samples that were incubated with AzBGO‐PC1 under irradiation with UV light (171 proteins in A549 cell lysate; 238 proteins in *E. coli* lysate). The number of identified proteins was significantly lower in the samples that were incubated with the chemically activated probe AzBGO or with AzBGO‐PC1 in absence of light. Heat maps were generated of the identified, enriched proteins to visualize these differences (**Figure** **6**A,B and **Figure** **7**A,B). These heatmaps show that the chemoproteomic data align well with the data obtained in the model studies on BSA, as well as the SDS‐PAGE labeling experiments on cell lysates. Compared to samples that were treated with the chemically activated probe AzBGO‐DMA, the number of proteins and the log_10_ intensities are considerably higher in the samples treated with the photocaged probe. Furthermore, the proteomics data from the nonirradiated, probe‐treated samples underline the importance of selecting appropriate controls in the proteomics experiments. As observed in the SDS‐PAGE, various proteins are labeled in the absence of photoactivation. These proteins react either with the ketone in the nonactivated probe or undergo side‐reactions with the reporter group used in the CuAAC step.^[^ ^36^ ^]^ Even though the latter may, at least in part, be suppressed by optimizing the click procedure and the click handle in the probe, the nonirradiated control samples are essential to discriminate between probe‐specific labeling and side reactions.

To validate that treating cell lysates with AzBGO yields results similar to MGO treatment, we performed a KEGG pathway analysis (Figure 6C) and compared the identified pathways to the components that were found to be enriched in the MGO‐induced crosslinking study by Chen et al.^[^ ^18^ ^]^ Even though their study was performed in different cell type (HEK), we did find that components of similar pathways were enriched when labeling A549 cell lysates. For example, both studies showed that ribosomal components, and components that are involved in Parkinson's disease, carbon metabolism and glycolysis/gluconeogenesis were significantly enriched for both MGO and AzBGO. Analysis of the data on a protein level reveals 21 out of the 66 unique proteins identified in the in situ cross‐linking studies by Chen et al. were found to be labeled by AzBGO‐PC1 in A549 lysate (Figure 6D).^[^ ^18^ ^]^

The proteins that reacted with AzBGO‐PC1 in A549 lysate included glycolytic enzymes, such as glucose‐6‐phosphate isomerase, fructose‐bisphosphate aldolase, glyceraldehyde‐3‐phosphate dehydrogenase and aldo‐keto reductase, various (oligo)nucleotide binding proteins, heat‐shock proteins, actins, and disulfide isomerases. These findings align with already existing literature.^[^ ^9^ ^,^ ^20^ ^,^ ^23^ ^]^

To investigate the correlation between the glycated proteins and the potential regulatory role in A549 cells, the biologically relevant pathways were analyzed by over‐representation analysis (ORA) using a custom‐made R script. This method measures the enrichment of a set of functionally related proteins relative to what can be expected to be present in the set of protein captured by the chemoproteomics workflow by chance. With this approach biologically relevant pathways with a specific function according to gene ontologies can be identified for the enriched protein. The ORA of the proteins uniquely retrieved in the samples that were incubated with photoactivated AzBGO‐PC1 showed a high enrichment of biological pathways engaged in the regulation of telomerase and in the Cajal bodies (Figure 6E, Figure S10, Supporting Information). Cajal bodies are subnuclear domains found in proliferative cells such as tumor cells and embryotic cells.^[^ ^42^ ^]^ They are involved in the biogenesis of enzyme telomerase as well as the transportation of telomerase to telomeres.^[^ ^43^ ^]^ The shortening of the telomeres, which protect the genome, is known to be associated with aging and the enzyme telomerase slows down and prevents this process. The dysfunctioning of regulatory processes on the Cajal body and telomeres can lead to excessive telomere shortening, which can no longer defend the cell effectively and hence cause the disease development. Moreover, the shortening of telomeres was in previous studies linked to oxidative stress which supports these results.^[^ ^44^ ^]^ A significant number of genes was found also in translation and protein folding (Figure 6E).

The proteins that were uniquely identified in the *E. coli* samples that were treated with light‐activated AzBGO‐PC1 were submitted for KEGG analysis and gene ontology enrichment analysis as well (Figure 7C,D, Figure S11, Supporting Information). The KEGG analysis revealed that carbon metabolism and ribosomal components are highly enriched in *E. coli* lysate that was treated with light‐activated AzBGO‐PC1. According to the ORA, most enriched proteins were associated with metabolic or biosynthetic processes. These findings are in line with the results of the mammalian cells. Inferring from the work of the Moellering lab,^[^ ^8^ ^]^ these results might point potentially toward the direction that glycation may act as a potential feedback loop in bacteria as well, but further studies will be required to validate this hypothesis.

Unfortunately, we could not identify the modified peptides in the dataset resulting from the peptides eluted from the beads after cleavage of the azo‐linker. MGO‐derived PTMs have limited stability. Hemithioacetal formation is highly reversible and the products are not stable under the conditions used to isolate the glycated peptides.^[^ ^23^ ^]^ The half‐life of dihydroxyimidazolidine and hydroimidazolones ranges from hours to days depending on the type of PTM and the pH,^[^ ^22^ ^]^ and low recoveries for this PTMs have been reported after sample processing. These analyses are further complicated by sulfonation of the diazo‐benzene cleavable linker.^[^ ^16^ ^]^ MGO‐derived cross‐linking products are considerably more stable,^[^ ^8^ ^,^ ^9^ ^]^ but the detection of crosslinked peptides is not straightforward and these were therefore excluded from the search. The poor recovery of modified peptides may be attributed to the combination of these behaviors.

## 3 Conclusion

In conclusion, we describe the synthesis and biochemical evaluation of a set of photocaged MGO probes and employ these probes to identify proteins that are susceptible to glycation in A549 and *E. coli* cell lysates. Labeling experiments on purified protein, as well as lysates demonstrate that the light‐activated probes AzBGO‐PC1, AlkMGO‐PC1, and AlkMGO‐PC2 have a higher potency compared to the chemically activated probes. Additional advantages are the higher stability and longer shelf life. Future studies will reveal if these photocaged variants of AzBGO and AlkMGO open the possibility to release MGO derivatives into the biological sample in situ in a controlled manner. The photocaged probes labeled numerous proteins in A549 cell lysate and *E. coli* cell lysate. Chemoproteomic analysis of the proteins that were susceptible to the AzBGO‐PC1 labeling and comparison to early studies further confirm that the subset of proteins that is labeled by the photocaged probes is similar to those that react with MGO. This was further supported by pathways analysis. The identified biological, cellular, and molecular pathways are comparable to those reported in earlier studies.

## 4 Experimental Section

4.1

4.1.1

##### General Biochemical Procedures: Cell Culture Conditions

The A549 cell line was grown in T75 culture flasks in Ham's F‐12K medium supplemented with 10% fetal bovine serum (FBS), 2 mM L‐glutamine, NaHCO_3_ (1500 mg L^−1^), or DMEM medium supplemented with 10% FBS, 4 mM L‐glutamine, glucose (4500 mg L^−1^), 1 mM sodium pyruvate, NaHCO_3_ (1500 mg L^−1^) in an incubator at 37 °C, and 5% CO_2_ humidified air. At about 70–90% confluency cells were detached from the flask by Trypsin/EDTA treatment, pelleted, and either allowed to continue to grow in 10 mL complete cell culture medium per T75 flask or washed 2 times with PBS and stored at −80 °C until they were lysed. Cells were passaged every 2–3 days by resuspending in a cell culture medium until appropriate confluence.

##### SDS‐PAGE and Western Blot Analysis

Laemmli‐type SDS‐PAGE was performed according to standard literature procedures. SDS‐PAGE gels were prepared using acrylamide‐bis ready‐to‐use solution 40% (37.5:1) (Merck Millipore) and separated on a Mini‐PROTEAN Tetra cell (Bio‐Rad). Fluorescence scanning of SDS‐PAGE gels was performed on a typhoon 9500 FLA model (GE Healthcare) using the Alexa Fluor 488 setting (green laser excitation at 473 nm and emission filter at 520 nm) for FAM alkyne labeled proteins. Coomassie staining was carried out with ReadyBlue Protein Gel Stain (purchased from Sigma–Aldrich) or with CBB G250 staining according to the manufacturer's protocol (Roti‐Blue, Carl Roth).

For western blot analysis, proteins were transferred to a nitrocellulose membrane or PVDF (GE Healthcare) after separation by gel electrophoresis using a Mini Trans‐Blot system for wet blotting (Bio‐Rad). The transfer was performed according to the manufacturer's protocol using a Tris‐Glycine buffer with 20% ethanol. Biotinylated proteins were either stained using Streptavidin‐DyLight 650 (Invitrogen: 84547) and visualized on a typhoon 9500 FLA model (GE Healthcare) using the CY5 setting (red laser excitation at 651 nm and emission filter at 670 nm) or stained using Streptavidin‐HRP and visualized on a Bio‐Rad ChemiDoc XRS imager. A pull‐down experiment was performed using NeutrAvidin‐agarose beads (Sigma–Aldrich).

##### Probes, Reagents, and Material: Preparation of a Stock Solution Glyoxal Probes and Chemical Activation

The dimethyl acetal protected probes (AzMGO‐DMA and AzBGO‐DMA) were hydrolyzed prior to the labeling by heating the probes in 5% aqueous sulfuric acid at 100 °C for 30 min. AlkMGO‐DMA was hydrolyzed prior to the labeling by heating the probe in 5% sulfuric acid in DMSO/H_2_O (1/1) at 100 °C for 30 min. The probe samples were cooled to room temperature after which the samples were neutralized with 1 M KOH. The final concentration of the probe was adjusted to 20 mM. The probe solutions were directly applied to the lysate solutions. The probe stock solutions were stored at −20 °C for a maximum of 1 week.

##### Preparation of the Photocleavable Probe Stocks Solutions

The photocaged probe was dissolved in DMSO in black nontransparent Eppendorf tube. The concentration of the stock solutions was 50, 20, and 2.5 mM and the stocks were stored at −20 °C.

##### Stock Solution of Other Reagents

Stock solutions of FAM‐alkyne (Lumiprobe), biotin‐alkyne, diazo biotin‐alkyne (SigmaAldrich, MFCD28505544), and azido‐PEG3‐biotin (Sigma–Aldrich, MFCD20134145) were prepared in DMSO and stored at 20 °C. Stock solutions of THPTA and TABTA ligands and CuSO_4_^.^·5H_2_O were prepared in deionized water and stored at −20 °C. The stock solution of sodium ascorbate was prepared fresh in water before the experiment. Sample buffer (SB) 4× contains 200 mM Tris‐HCl (pH 6.8), 400 mM dithiothreitol (DTT), 8% sodium dodecyl sulfate (SDS), 0.4% bromophenol blue and 40% glycerol. Stock solutions of DTT and of iodoacetamide for pull‐down were prepared in DMSO in a dark nontransparent Eppendorf tube and both were stored at −20 °C.

##### Buffers and Others

The blotting solution of 1:12,500 Strep‐DyLight 650 used in western blot was prepared by diluting 2 µL of purchased Strep‐DyLight 650 in ≈25 mL of 1% BSA in TBS‐T. The blotting solution of 1:12,500 Strep‐HRP used in western blotting was prepared by diluting 2 µL of purchased Strep‐HRP in ≈25 mL of 0.5% BSA in TBS‐T.

##### Labeling of the Synthetic Peptide

The stock solution of **AzBGO‐PC1** was prepared (0.76 mg) in DMSO (100 µL) with the final concentration of 20 mM. The synthetic peptide with the sequence FGERAFK (0.170 mg) was added to a solution of **AzBGO‐PC1** (10 eq. resp. to peptide). The peptide and the reagent mixture was irradiated with 365 nm lamp for 15 min, and then left to incubate at 100 °C for 1 h and then at 60 °C for overnight. The sample was then measured on LC–MS/MS using Orbitrap Velos system and the acquired data were analyzed with XCalibur and PEAKSXPro Studio.

##### SDS‐PAGE Labeling of Bovine Serum Albumin: Labeling of BSA with the MGO Probes (Figure 2)

AzBGO (1 µL of a 10× stock solution) was added to BSA (1 mg mL^−1^) in PBS (9 µL, pH 7.4). The final probe concentration in the labeling mixture was 0.5, 1, and 2 mM and the mixture was incubated for the indicated time (2, 4, 6, 24 h) at 37 °C. Then, the protein solution was denatured by adding SDS (1% w/v final concentration) and heating at 100 °C for 5 min. Click reaction mix (5‐FAM‐alkyne (1 µL, 10 mM), CuSO_4_ (1 µL, 20 mM), THPTA (1 µL, 20 mM), NaAsc (1 µL, 10 mM)) was added to the protein solution and mixture was incubated for 2 h. The reaction was subsequently quenched by the addition of SDS‐PAGE sample buffer and loaded onto a 12% SDS‐PAGE gel. The gel was left to run at 150 V for 1 h and 20 min. The in‐gel fluorescence was scanned on a Typhoon FLA 9500 using the Cy5 setting and afterward stained in Coomassie Brilliant Blue.

##### Labeling of BSA with AzBGO‐PC1 (Figure 4B,C)

AzBGO‐PC1 (1 µL of a 10× stock solution) was added to BSA (1 mg mL^−1^) in PBS (9 µL, pH 7.4). The final probe concentration in the labeling mixture was 0.5, 1, and 2 mM. The samples were then either incubated in the dark for 2 h at RT, or were exposed to UV light of 365 nm wavelength. The light‐treated samples were irradiated for 15 min. The protein solution was then denatured by the addition of SDS (1% final concentration) and heated at 100 °C for 5 min. Click reaction mix (5‐FAM‐alkyne (1 µL, 10 mM), CuSO_4_ (1 µL, 20 mM), THPTA (1 µL, 20 mM), NaAsc (1 µL, 10 mM)) was added to the protein solution and the mixture was incubated for 2 h. The reaction was subsequently quenched by the addition of SDS‐PAGE sample buffer and loaded onto a 12% SDS‐PAGE gel. The gel was left to run at 150 V for 1 h and 20 min. The in‐gel fluorescence was scanned on a Typhoon FLA 9500 using the Cy2 setting and afterward stained in Coomassie Brilliant Blue.

##### Labeling of BSA with AlkMGO Derivatives (Figure 5)

AlkMGO‐derivative (10 µL of a 2.5 mM stock, 250 μM final concentration) was added to BSA (1 mg mL^−1^) in PBS (90 µL, pH 7.4). The samples were then either incubated in the dark for 1 h at RT, or were exposed to UV light of 365 nm wavelength for 1 h. The excess probe was removed by extraction with diethyl ether (200 µL, twice). The protein solution was then denatured by the addition of SDS (1% final concentration) and heated at 100 °C for 5 min. An analytical sample was taken (10 µL). Biotin‐PEG3‐azide (1 µL of a 20 mM stock), CuSO_4_ / THPTA (1 µL of a 20 mM stock), and NaAsc (1 µL of a 20 mM stock) were added to this sample and the mixture was incubated overnight. The reaction was subsequently quenched by the addition of SDS‐PAGE sample buffer and loaded onto a 12.5% SDS‐PAGE gel. The proteins were transferred to a PVDF membrane with a Mini‐Trans blot system for wet blotting (Bio‐Rad), according the manufacturers protocol. The membrane was blocked with BSA (0.5% in TBS‐T) for 1 h and probed with Strp‐HRP (1:12,500, 0.5% in TBS‐T). The membranes were washed with TBS‐T (3x) and TBS. The biotinylated proteins were visualized with Clarity Western ECL Plus substrate on a ChemiDoc XRS (Bio‐Rad) according to the manufacturer's protocol. Afterward, the blots were stained with ReadyBlue Protein Gel Stain (purchased from Sigma–Aldrich).

##### Labeling of BSA for MS Analysis

AlkMGO‐derivative (10 µL of a 2.5 mM stock, 250 μM final concentration) was added to BSA (1 mg mL^−1^) in PBS (90 µL, pH 7.4). The samples were then either incubated in the dark for 1 h at RT, or were exposed to UV light of 365 nm wavelength for 1 h. The samples were subjected to a standard proteomics workflow.

##### In Vitro Glycation on Cell Lysates: Preparation of A549 Cell Lysate

A549 cells were lysed using a freshly prepared NP40 lysis buffer [0.5% NP40, Tris HCl (10 mM), NaCl (150 mM), MgCl_2_ (5 mM), pH 7.4] during 10 min over ice. The A549 lysates were then centrifuged for 10 min at 10,000 rpm and after the collection of the supernatant, the protein concentration was assessed by Bradford protein assay.

##### A549 Labeling (Figure 4C)

A549 cell lysate (18 µL, 0.7 mg mL^−^^1^ in PBS, pH 7.4, containing 0.5% (v/v) NP 40) was incubated with the indicated concentration of AzBGO‐PC1 (the final concentration 250, 100, 50, 25, 10 µM). Then, the samples were exposed to 365 nm UV light for 15 min. As controls, samples containing DMSO or probe (250 µM) incubated in the dark were included. The labeling mixture was subsequently denatured by the addition of SDS (2 µL of a 10× stock, 1% final w/v) and heated at 100 °C for 5 min. Click reaction mix (5‐FAM‐alkyne (2 µL, 5 mM), CuSO_4_ (2 µL, 20 mM), THPTA (2 µL, 20 mM), and NaAsc (2 µL, 10 mM)) was added to the protein solution and the mixture was incubated at RT for 2 h. The reaction was subsequently quenched by the addition of SDS‐PAGE sample buffer and loaded onto a 12% SDS‐PAGE gel. The gel was left to run at 150 V for 1 h and 20 min. The in‐gel fluorescence was scanned on a Typhoon FLA 9500 using the Cy2 setting and afterward stained in Coomassie Brilliant Blue.

##### Pulldown Experiment with A549 Lysate

The A549 cell lysate (90 µL, 2 mg mL^−1^) and AzBGO‐PC1 (10 µL, 2.5 mM) were irradiated with UV light (365 nm) for 15 min. Subsequently, the samples were subjected to MeOH:CHCl_3_ protein precipitation. In the following order, methanol (400 µL), chloroform (300 µL), and water (300 µL) were added. The solution was then centrifuged (9000 × *g*, 30 min), the upper layer was carefully removed, and MeOH (300 µL) was added. Followed by centrifugation (9000 × *g*, 30 min), the liquid layer was discarded and the pellet of the proteins was air dried. Subsequently, the pellet was suspended in PBS (17 µL) containing SDS (1%) and heated to 100 °C for 5 min to dissolve the proteins. Subsequently, the click reaction reagents were added: biotin‐alkyne (1 µL, 8 mM), CuSO_4_ (0.5 µL, 20 mM), TBTA (0.5 µL, 20 mM), and NaAsc (1 µL, 10 mM). The reaction was incubated in the dark at RT for 2 h. Subsequently, the reagents were removed by another MeOH:CHCl_3_ protein precipitation and the protein pellet was dissolved in PBS (200 µL, with 0.1% SDS). The solution was sonicated and heated at 60 °C for 5 min. Then prewashed neutravidin (50 µL) beads were added and the mixture was left to rotate at RT for 2 h. The beads were centrifuged for 2 min. at 1400 × *g*, and 95% of the supernatant was removed. The beads were transferred into the bio spin column and washed consecutively with 1% SDS in PBS (3 × 100 µL), 6 M urea in H_2_O (3 × 100 µL) and PBS buffer pH 7.4 (5 × 100 µL). Next, the beads were transferred into low‐adhesion Eppendorf tubes and centrifuged for 2 min at 1400 × *g,* after which the supernatant was removed and discarded. The beads were resuspended in 200 µL 6 M urea. Afterward, DTT (10 µL, 200 mM) was added to the beads, and the suspension was heated at 65 °C for 15 min. Then, IAM (10 µL, 500 mM) was added and the beads were left to rotate for 30 min in the dark. The samples were then centrifuged for 2 min at 1400 × *g.* The supernatant was removed and the beads were resuspended in 2 M urea in PBS (200 µL). CaCl_2_ (2 µL, 100 mM) and trypsin (4 µL, 0.5 mg mL^−1^) were added and the mixture was left to incubate overnight at 37 °C. After the trypsin digestion, the beads were again transferred into a biospin column. The tryptic peptides were eluted with 5% FA in PBS (300 µL), freeze‐dried, and redissolved in a loading solution (2% CH_3_CN, 98% H_2_0, 0.1% TFA). The labeled peptides were eluted with 50% CH_3_CN/0.1% TFA and heated at 65 °C. After removal of the solvent, the samples were dissolved in loading solution (2% CH_3_CN, 98% H_2_0, 0.1% TFA) and analyzed by MS.

##### LC–MS/MS Analysis

The samples were analyzed on a nano‐LC–MS/MS consisting of an Ultimate 3000 LC system (Thermo Fisher Scientific, USA) interfaced with a Q Exactive Plus mass spectrometer (Thermo Fisher Scientific). Peptide mixtures were loaded onto a 5 mm × 300 μm i.d. C18 PepMAP100 trapping column with water with 0.1% formic acid at 20 μL min^−1^. After loading and washing for 3 min, peptides were eluted onto a 15 cm × 75 μm i.d. C18 PepMAP100 nanocolumn (Thermo Fisher Scientific). A mobile phase gradient at a flow rate of 300 nL min^−1^ and with a total run time 120 min was used: 2–45% of solvent B in 92 min; 45–85% B in 1 min; 85% B during 6 min, and back to 2% B in 0.1 min. Solvent A was 100:0 water/acetonitrile (v/v) with 0.1% formic acid, and solvent B was 0:100 water/acetonitrile (v/v) with 0.1% formic acid. In the nanospray source, a stainless‐steel emitter (Thermo Fisher Scientific) was used at a spray voltage of 2 kV with no sheath or auxiliary gas flow. The ion transfer tube temperature was 250 °C. Spectra were acquired in data‐dependent mode with a survey scan at *m*/*z* 375–1600 at a resolution of 70 000 followed by MS/MS fragmentation of the top 10 precursor ions at a resolution of 17 500. Singly charged ions were excluded from MS/MS experiments and fragmented precursor ions were dynamically excluded for 20 s.

##### Proteomics Identification and Statistical Analysis

Raw files were analyzed using PEAKS Studio Xpro (Bioinformatics solution Inc, Waterloo, Canada) was used for peptide and protein identification with false discovery rate (FDR) < 1% at peptide‐spectrum‐match (PSM), peptide and protein level and using decoy approach for FDR calculation.^[^ ^38^ ^]^ PSM is a general term used in bottom‐up proteomics and expresses the match between a fragment ion spectrum (MS/MS) and the (most probable) peptide sequence derived from the protein sequence database used in database search (PEAKS). FDR calculation was performed for each analyzed sample. For database search, human SwissProt canonical sequences downloaded on May 19, 2022 were used with the following settings: precursor error tolerance 10 ppm; precursor mass search type: monoisotopic; fragment mass error tolerance: 0.02 Da; cleavage enzyme: trypsin with 3 missed cleavages and usage of only tryptic peptides. Carbamidomethylation was used for fixed modification, and methionine oxidation was used as a variable modification for the identification of proteins enriched on the neutravidin column. The fraction containing the covalently bound peptides to neutravidin beads included additional variable modifications, with 6 maximal variable PTMs per peptide. Quantified proteins were exported in tsv format and were used for further statistical analysis.

CRAN R (version 4.1.2) with RStudio (version 2023.02.0 build 386) was used for post‐statistical analysis with ggplot2 (version 3.4.2) for visualization, ComplexHeatmap (version 2.9.3) for heatmap creation, and clusterProfiler (version 4.0.5) for ORA analysis as main packages. The data processing (PEAKS and R analysis) was performed on a PC equipped with Intel Core i7 8700 K processing, 64 GB DDR3 RAM and 2TB sata SSD.

## Conflict of Interest

The authors declare no conflict of interest.

## Supporting information

Supplementary Material

## Data Availability

Proteomics data is available via ProteomeXchange. Project accession: PXD061990; Project DOI: 10.6019/PXD061990. NMR spectra are available in the supporting information.
