# Supplementary material for: Identification of Methylglyoxal Reactive Proteins with Photocaged Glycating Agents
Source: Chembiochem. 2025 Sep 29;26(21):e202500275. doi: 10.1002/cbic.202500275 (PMC12596920; doi:10.1002/cbic.202500275)

Supplementary information of:

Identification of methylglyoxal reactive proteins with photocaged glycating agents

S. Sokoliova[a], I. R. Sardaru[a], F. P. Warguła[a], J. H. Hermans[b], H. P. Permentier[b], P. L. Horvatovich[b]*, M. D. Witte[a]*

[a] Stratingh Institute for Chemistry, University of Groningen, 9747 AG Groningen, The Netherlands

[b] Department of Analytical Biochemistry, Groningen Research Institute of Pharmacy, University of Groningen; Groningen, the Netherlands

Synthesis of the probes

The synthesis of the dimethyl acetal (DMA) protected versions of AzMGO and AlkMGO commenced from methylglyoxal 1,1-dimethyl acetal **1** (Scheme S1). Bromination of **1** yielded bromomethylglyoxal **2** (Scheme S1).[1] However, substitution of the bromide with sodium azide gave a complex mixture. The limited stability of the precursor and the product made us decide to not investigate this probe further. To prepare the butynylglyoxal probe (AlkMGO), methylglyoxal 1,1-dimethyl acetal **1** was reacted with cyclohexylamine (Scheme S1) to form *N*-cyclohexyl-1,1-dimethoxypropan-2-imine **3**.[2] The imine intermediate was used without further purification in a subsequent alkylation reaction. Cyclohexylimine dimethyl acetal **3** was treated with LDA, after which the enamine salt was reacted with propargyl bromide.[2] Upon acidic work up, the imine intermediate hydrolyzed giving butynylglyoxal dimethyl acetal (AlkMGO-DMA) in excellent yield over two steps (Scheme S1).

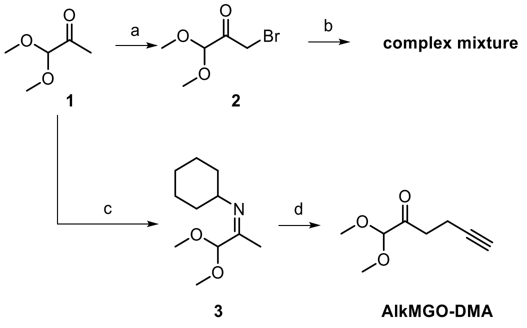

Scheme S1. Synthesis of AzMGO-DMA and AlkMGO-DMA. Reagents and conditions: a) Br_2, ACN/MeOH (47%); b) NaN_3, DMF (33%); c) cyclohexylamine, $CaCl_2$, Et_2O, 45℃, 16 h (76%); d) LDA, THF 1h, propargyl bromide -50 ℃, then RT 48h (85%)

Azidobutylglyoxal dimethyl acetal (AzBGO-DMA) was synthesized from commercially available 6-bromo-1-hexene **4** in five steps. Dihydroxylation of 6-bromo-1-hexene **4** with AD-mix gave the resulting in diol **5** in 94% yield.[3] Substitution of the bromide with sodium azide gave known azido diol **6**.[4] The primary alcohol group in **6** was then selectively oxidized and afterwards immediately reacted with trimethyl orthoformate to form dimethyl acetal **7**. The last step was the oxidation of the remaining alcohol group with Dess-Martin periodinane resulting in final probe AzBGO-DMA in yield 46%.

Formatted: Font color: Auto
Formatted: Font color: Auto
Formatted: Font color: Auto
Formatted: Font color: Auto
Formatted: Font color: Auto
Formatted: Font color: Auto
Formatted: Font color: Auto
Formatted: Font color: Auto

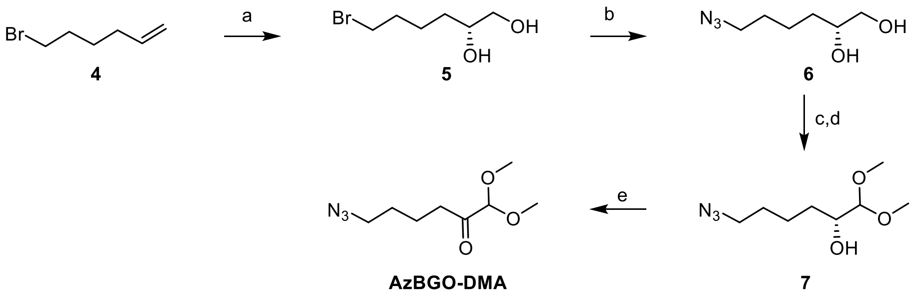

Scheme S2. Synthesis of azidobutylglyoxal probe AzBGO-DMA. a) AD-mix-alpha, DCM/H_2O/*t*-BuOH (94%); b) NaN_3, DMF (96%); c) TEMPO, TCICA, EtOAc (46%); d) trimethyl orthoformate, *p*-TsOH, MeOH (80%); e) Dess-Martin periodinane, $NaHCO_3$, DCM (46%).

Synthesis of the photocages. The nitrobenzene photocage **PC1** was prepared from 6-nitroveratraldehyde **8** in two steps (Scheme S3) according to the reported literature procedure. Wittig olefination yielded in alkene **9**.[6] A subsequent dihydroxylation reaction using AD-mix-alpha gave diol **PC1**. The synthesis of nitrobenzene diol **PC2** commenced with a pyrrolidine-catalyzed aldol reaction between 6-nitroveratraldehyde and isobutyraldehyde, which gave the β-hydroxy aldehyde **11** in 60%. Unreacted 6-nitroveratraldehyde was still present in the reaction mixture. Attempts to remove the starting material were unsuccessful, since the starting material co-eluted with the product. Addition of an additional amount of isobutyraldehyde, along with catalysts improved the conversion, but came at the expense of the formation of self-condensation side products. It was therefore decided to reduce the aldehyde with sodium borohydride and to purify the photocage in the final stage. Reduction with $NaBH_4$ went uneventful and yielded the product **PC2** in 44% yield over two steps.

Formatted: Font color: Auto

Formatted: Font color: Auto

Formatted: Font color: Auto

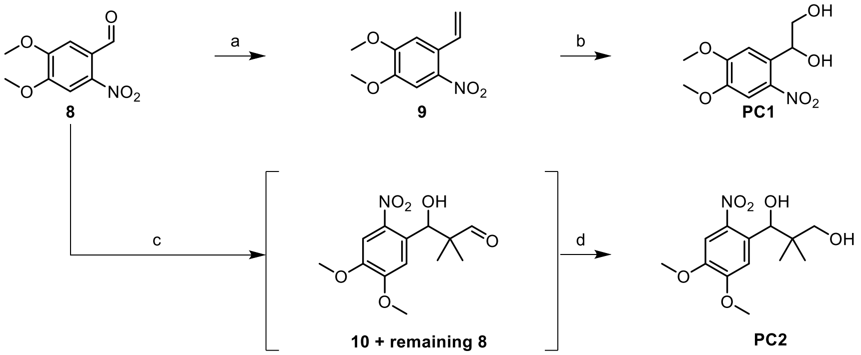

Scheme S3. a) $MePPh_3Br$, NaHMDS, THF (72%); b) AD-mix-alpha, $DCM/H_2O/t\text{-}BuOH$ (86%); c) pyrrolidine (0.05 equiv), AcOH (0.25 equiv), isobutyraldehyde (1 equiv), DMSO, RT (60%); d) $NaBH_4$, MeOH (44% over two steps)

Synthesis of photocaged probes.

We initially attempted to convert AzBGO-DMA and AlkMGO-DMA into the corresponding photocaged probes by performing a transacetalization reaction with the photocages PC1 and PC2. However, the ketone group in AzBGO-DMA and AlkMGO-DMA reacted as well, according to the NMR analysis. To circumvent this side reaction, we performed the transacetalization on the corresponding alcohols **7** and **11**. Alcohol **7** was an intermediate of the synthesis of AzBGO-DMA and alcohol **11** was readily obtained by reducing the keto-functionality in AlkMGO with sodium borohydride. Transacetalization of the **7** and **11** provided the desired photocaged products **12-14**. The last reaction in the preparation of **AzBGO-PC1**, **AlkMGO-PC1** and **AlkMGO-PC2** was oxidation of the alcohol with Dess-Martin periodinane, giving final product. AlkMGO-PC2 could also be prepared by direct transacetalization of AlkMGO-DMA, but the forcing conditions used in these reactions did lead to isomerization of the alkyne.

Formatted: Font color: Auto

Formatted: Font color: Auto

Formatted: Font color: Auto

Formatted: Font color: Auto

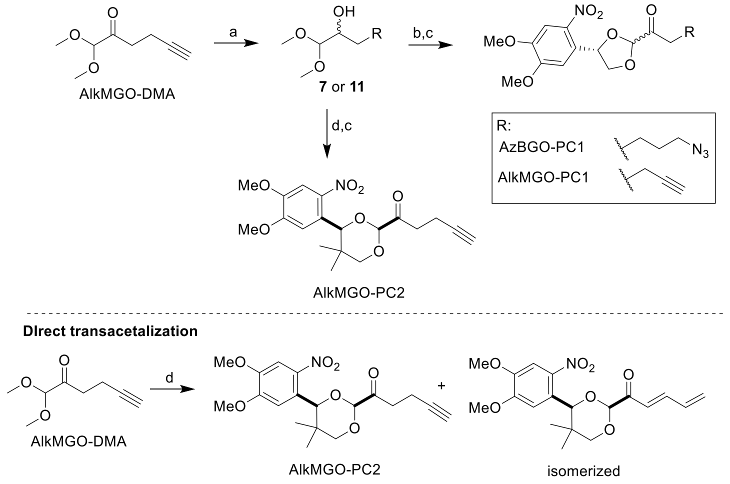

Scheme 4. a) $NaBH_4$, MeOH (83%); b) CSA, DCM (67% for transacetalization on **7**, 58% for transacetalization on **11** with PC1); c) Dess-Martin periodinane, $NaHCO_3$, DCM (62% for oxidation of **12**, 93% for oxidation of **13** and 83% for oxidation of **14**).

Formatted: Font color: Auto

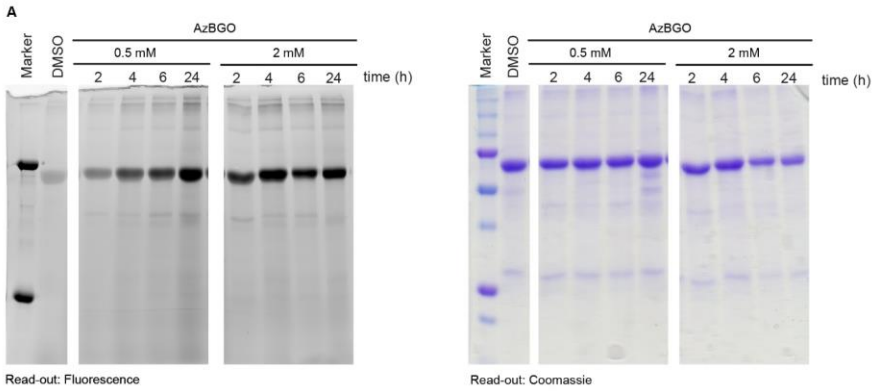

Figure S1. Uncropped images of the gels depicted in figure 2

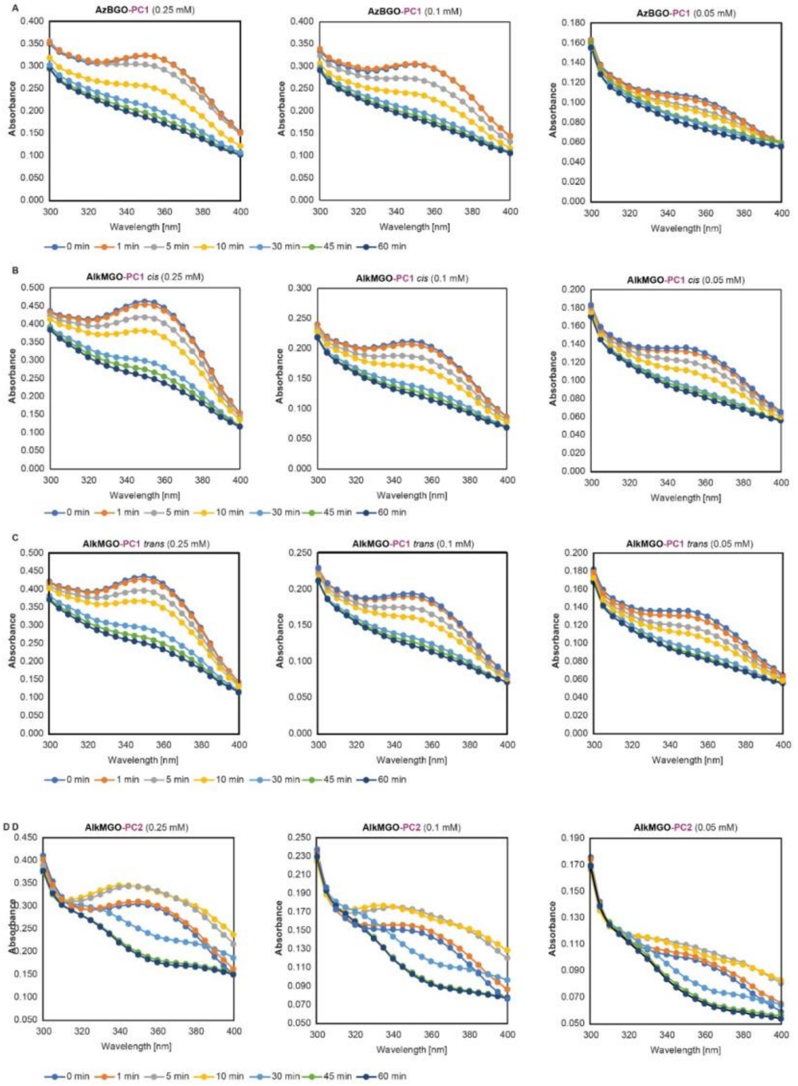

Figure S2. Uncaging of the AzBGO-PC1 (A), AlkMGO-PC1 cis (B), AlkMGO-PC1 trans (C) and AlkMGO-PC2 (D) monitored with UV-vis over one hour. A solution (100 µL) of the indicated probe concentration in HEPES containing 10% DMSO was placed in a 96-well plate. The plate was irradiated with a handheld UV light (365 nm). After the indicated irradiation time, the absorbance was measured with a plate reader.

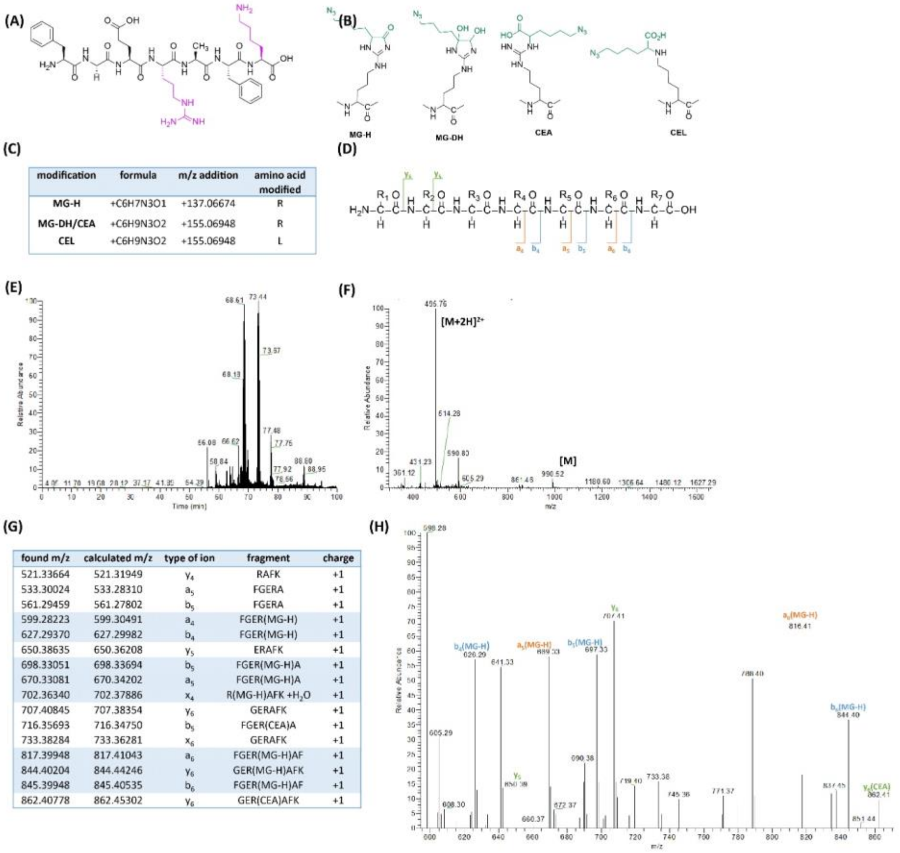

modification	formula	m/z addition	amino acid modified
**MG-H**	+C6H7N3O1	+137.06674	R
**MG-DH/CEA**	+C6H9N3O2	+155.06948	R
**CEL**	+C6H9N3O2	+155.06948	L

found m/z	calculated m/z	type of ion	fragment	charge
521.33664	521.31949	y_4	RAFK	+1
533.30024	533.28310	a_5	FGERA	+1
561.29459	561.27802	b_5	FGERA	+1
599.28223	599.30491	a_4	FGER(MG-H)	+1
627.29370	627.29982	b_4	FGER(MG-H)	+1
650.38635	650.36208	y_5	ERAFK	+1
698.33051	698.33694	b_5	FGER(MG-H)A	+1
670.33081	670.34202	a_5	FGER(MG-H)A	+1
702.36340	702.37886	x_4	R(MG-H)AFK +H_2O	+1
707.40845	707.38354	y_6	GERAFK	+1
716.35693	716.34750	b_5	FGER(CEA)A	+1
733.38284	733.36281	x_6	GERAFK	+1
817.39948	817.41043	a_6	FGER(MG-H)AF	+1
844.40204	844.44246	y_6	GER(MG-H)AFK	+1
845.39948	845.40535	b_6	FGER(MG-H)AF	+1
862.40778	862.45302	y_6	GER(CEA)AFK	+1

Figure S3. Modification of the model peptide. (A) structure of the model peptide. (B) Structures of MG-H, MG-DH, CEA. (C) Molecular mass of the added PTMS. (D) b and y ions. (E) Chromatogram of the modification reaction. Total ion counts are depicted. (F) MS1 spectrum of the modified peptide. (G) Table with the expected molecular mass of the fragments. (H) MS/MS spectrum of the modified peptide.

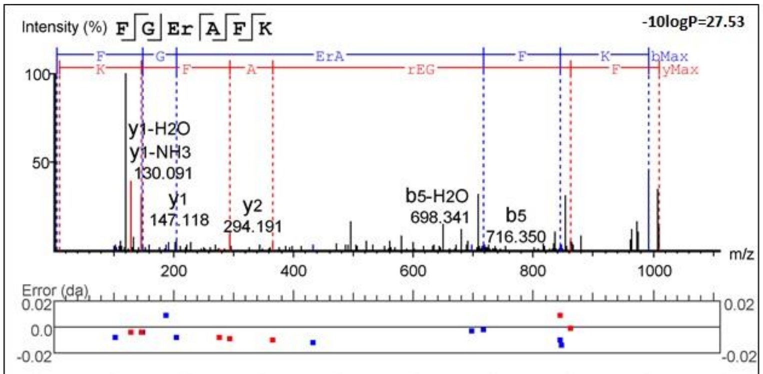

#	b	b-H2O	b-NH3	b (2+)	Seq	y	y-H2O	y-NH3	y (2+)	#
1	148.081	130.066	131.049	74.538	F					7
2	205.106	187.077	188.071	103.057	G	862.454	844.442	845.416	431.726	6
3	334.140	316.130	317.113	167.570	E	805.431	787.421	788.404	403.216	5
4	645.311	627.300	628.284	323.155	R(+155.07)	676.389	658.378	659.362	338.694	4
5	716.350	698.341	699.321	358.674	A	365.229	347.208	348.191	183.109	3
6	863.416	845.416	846.404	432.220	F	294.191	276.171	277.163	147.591	2
7					K	147.118	129.107	130.091	74.056	1

Figure S4. The fragment spectrum of FGERAFK model peptide with CEA modification at arginine residue providing +155.06948 Da shift as results of database search using PEAKS Xpro software.

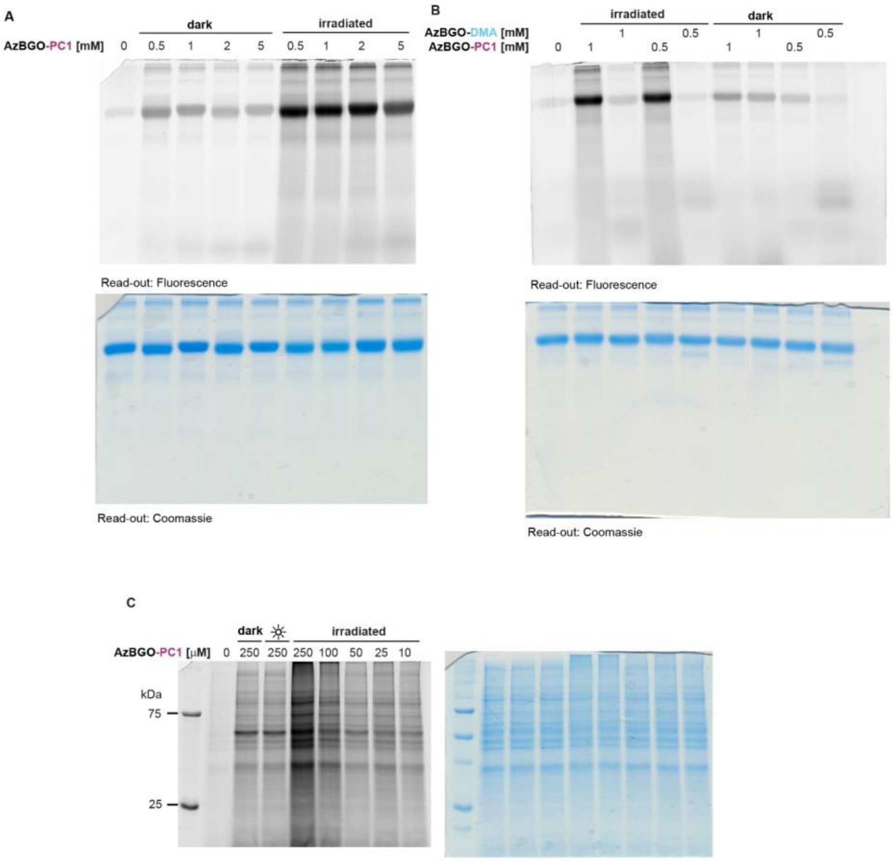

Figure S5. Uncropped gel and coomassie brilliant blue stain of the gel depicted in Figure 4.

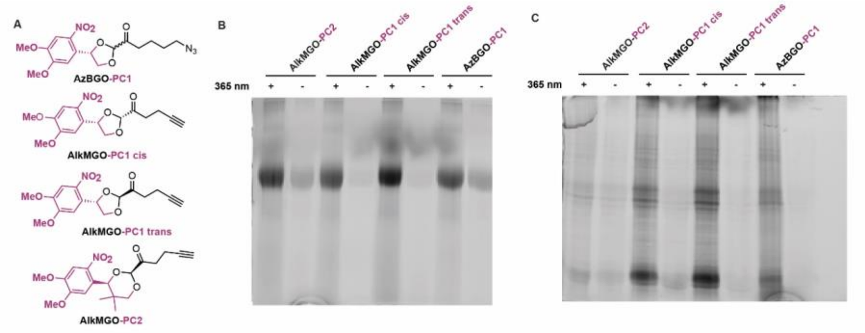

Figure S6. (A) Labeling of bovine serum albumin with the photocaged glycating agents **AlkMGO-PC1 cis**, **AlkMGO-PC1 trans**, **AlkMGO-PC2** and **AzBGO-PC1**. A solution of BSA (1 mg/ml) in PBS (pH 7.4) was incubated with the probes (0.2 mM) for one hour either while being exposed to 365 nm light or being kept in the dark. (B) Labeling of *E. coli* lysate with the photocaged glycating agents **AlkMGO-PC1 cis**, **AlkMGO-PC1 trans**, **AlkMGO-PC2** and **AzBGO-PC1**. A solution of *E. coli* lysate (2 mg/ml) in PBS (pH 7.4) was incubated with the probes (0.25 mM) for one hour either while being exposed to 365 nm light or being kept in the dark. The proteins were precipitated to remove the probe excess. Read-out: The probe-labeled proteins were conjugated to Cy5-azide by CuAAC and the proteins were visualized by scanning the in-gel fluorescence read-out on a Typhoon FLA9500.

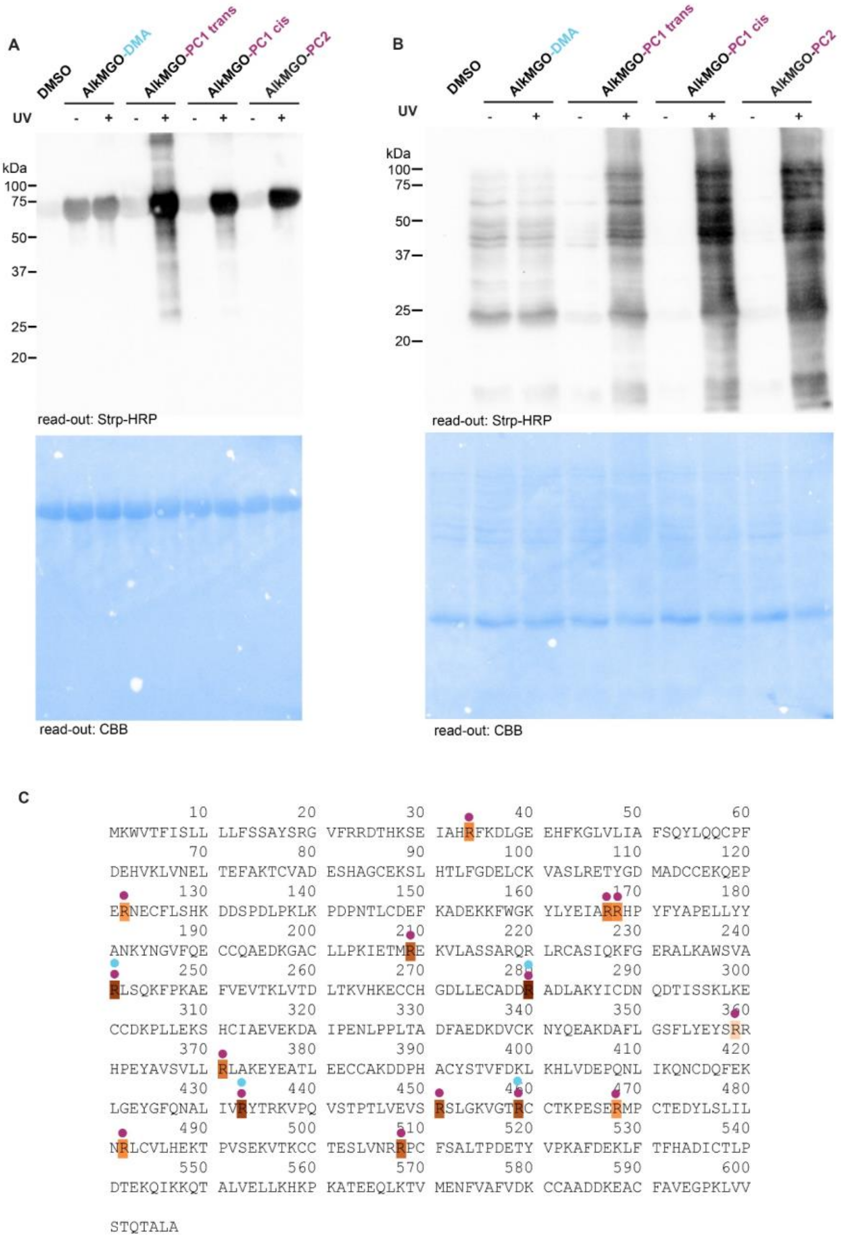

Figure S7. Labeling of bovine serum albumin with the photocaged glycating agents **AlkMGO-PC1 cis**, **AlkMGO-PC1 trans**, **AlkMGO-PC2** and the control probe **AlkMGO-DMA**. (A) A solution of BSA (1

mg/mL, 90 µL) in PBS (pH 7.4) was incubated with the probes (10 µL of a 2.5 mM stock, final concentration: 250 µM) for one hour either while being exposed to 365 nm light or being kept in the dark. (B) Labeling of *E. coli* lysate with the photocaged glycating agents **AlkMGO-PC1 cis**, **AlkMGO-PC1 trans**, **AlkMGO-PC2** and the control probe **AlkMGO-DMA**. A solution of *E. coli* lysate (2 mg/ml, 90 µL) in PBS (pH 7.4) was incubated with the probes (10 µL of a 2.5 mM stock, final concentration: 250 µM) for one hour either while being exposed to 365 nm light or being kept in the dark. Excess probe was removed by an extraction with diethyl ether. Read-out: The probe-labeled proteins were conjugated to biotin-PEG3-azide by CuAAC, the proteins separated on an 12.5% SDS PAGE and transferred to a PVDF membrane. The membranes were probed with Strp-HRP and the labeled proteins were visualized with ECL+ on a BioRad imager. (C) Sequence of BSA with the modified arginine residues highlighted. The color coding corresponds to the heat-map of **AlkMGO-PC1** in Figure 5C. Residues that were identified in the **AlkMGO-PC1** treated samples are indicated with the pink circles. Residues that were identified in the **AlkMGO-DMA** treated samples are indicated with the sky blue circles.

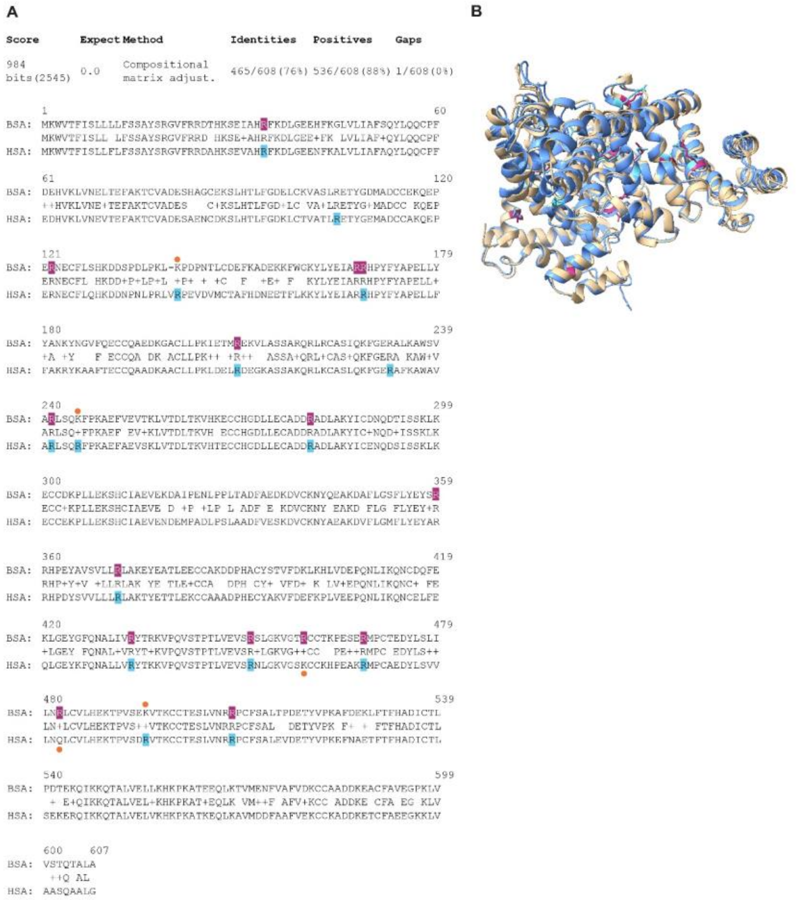

Figure S8. Comparison of the glycation of BSA by **AlkMGO-PC1** and the reported glycation of HSA by **AlkMGO**. (A) Sequence alignment of BSA and HSA. Labeled residues are highlighted with pink (BSA, **AlkMGO-PC1**) and sky blue (HSA, **AlkMGO-DMA**, chemically activated). Residues that are not conserved are labeled with an orange circle. (B) Structural alignment of BSA and HSA with the modified residues color-coded in the same manner as described for (A).

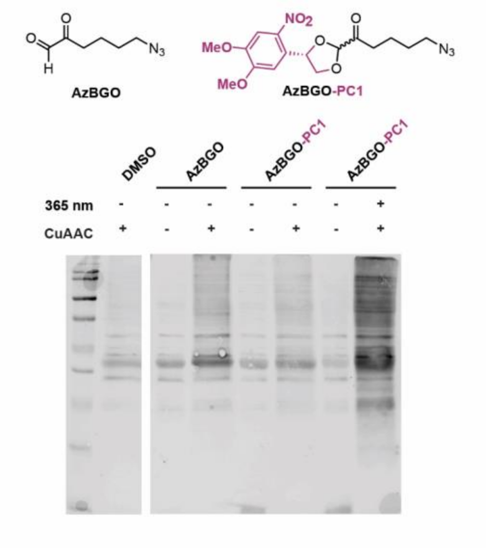

Figure S9. Western blot of E. coli cell lysate labeled with AzBGO-PC1.

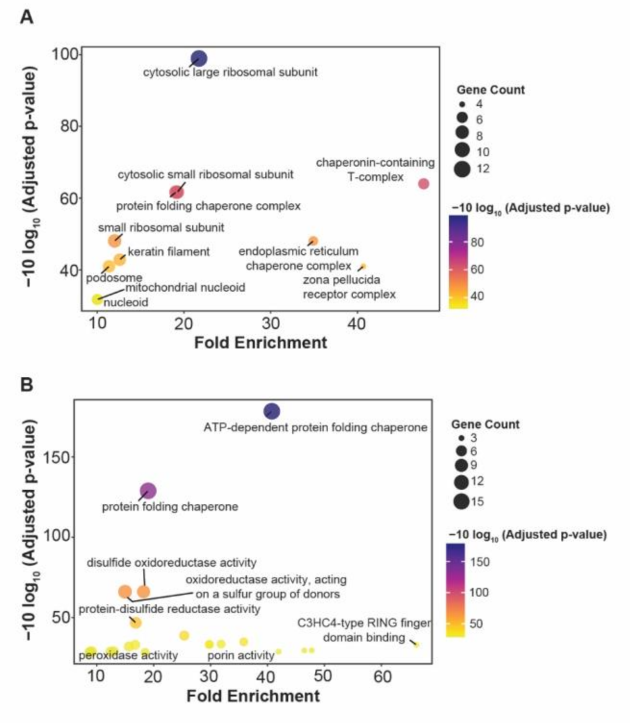

Figure S10. Bubble chart of the ORA of proteins identified in A549 cell lysate that was treated with AzBGO. The proteins are clustered based on the cellular component (A) and the molecular function (B). The circle size shows the number of proteins in the enriched pathways.

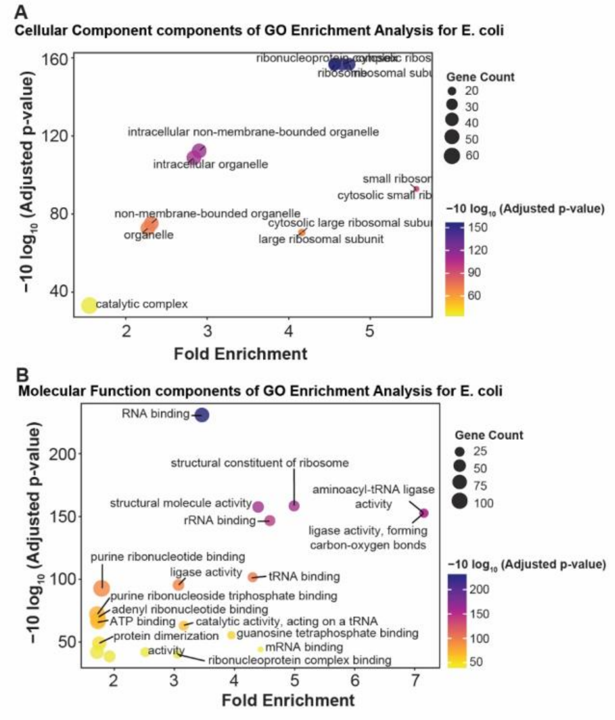

Figure S11. Bubble chart of the ORA of proteins identified in *E. coli* cell lysate that was treated with AzBGO. The proteins are clustered based on the cellular component (A) and the molecular function (B). The circle size shows the number of proteins in the enriched pathways.

Experimental section

General procedures

^{1}H and ^{13}C NMR were recorded on a Varian AMX400 spectrometer or on a Bruker Avance NEO 600, using chloroform-*d*, DMSO-d_6, deuterium oxide or methanol-d_4 as solvent. Chemical shift values are reported in ppm with the solvent resonance as the internal standard (chloroform-*d*: δ 7.26 for ^{1}H, δ 77.16 for ^{13}C; methanol-d_4: δ 3.31 for ^{1}H, δ 49.00 for ^{13}C; DMSO-d_6: δ2.50 for ^{1}H δ 39.52 for ^{13}C; D_2O: δ 4.79 for ^{1}H). Data are reported as follows: chemical shifts (δ), multiplicity (s = singlet, d = doublet, dd = double doublet, ddd = double double doublet, t = triplet, q = quartet, p = quintet, m = multiplet), coupling constants J (Hz), and integration. High resolution mass measurements were performed using a ThermoScientific LTQ OrbitrapXL spectrometer using methanol as eluent.

3-bromo-1,1-dimethoxypropan-2-one 2

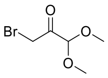

Methylglyoxal-1,1-dimethyl acetal (1 g, 8.5 mmol) was dissolved in 5% (v/v) methanol/acetonitrile (4 mL) and cooled to 0°-5° C. A solution of bromine (0.43 mL, 8.5 mmol) in acetonitrile (1 mL) was prepared and added to the methylglyoxal-1,1-dimethyl acetal solution in portions. The resulting mixture was left to stir overnight. The mixture was filtered over celite and the filtrate was concentrated under reduced pressure using a bath temperature of 35° C. The crude product was disolved in Et_2O and the mixture was washed with a sat. aqueous solution of $NaHCO_3$ and water. The organic layer was dried (over $MgSO_4$, filtered and concentrated *in vacuo*. The product was purified by automated flash chromatography column (linear gradient: 0% to 20% EtOAc in pentane in 5 min, then isocratic elution was performed in 20% EtOAc in pentane) to give the product **2** yield in 32% (532 mg). ^{1}H NMR (400 MHz, Chloroform-*d*) δ 4.72 (s, 1H), 4.20 (s, 1H), 3.45 (s, 6H). ^{13}C NMR (101 MHz, Chloroform-*d*) δ 196.3, 102.7, 55.1, 31.5. HRMS (ESI+) *m/z* calculated for $[M+Na]^+$ 218.9633, found 218.9629.[1] *Note: purification of bromoketone is quite challenging due to its limited stability, especially at higher temperatures. This results in some impurities in NMR spectra of the product and further products prepared from **2**.*

Formatted: Font color: Auto

Formatted: Font color: Auto

***N*-cyclohexyl-1,1-dimethoxypropan-2-imine 3**

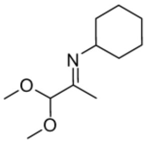

Adapted from the procedure reported by Patel et al.[5] Methylglyoxal 1,1-dimethyl acetal (2.5 mL, 20.65 mmol, 1 eq.) was added to a mixture of cyclohexylamine (2.6 mL, 22.72 mmol, 1.1 eq.) and calcium chloride (120 mg) in diethyl ether (15 mL). The resulting reaction mixture was stirred at 45 °C for 16 h. The reaction was monitored by TLC (40% ethyl acetate in pentane). After the mixture was cooled to room temperature, the solution was filtered and the white solids were washed with diethyl ether. The solvent was evaporated to give compound **3** as a clear orange oil (3.11 g, 15.6 mmol, 76% yield): ^{1}H NMR (400 MHz, Chloroform-d) δ 4.42 (s, 1H), 3.39 (s, 6H), 3.37-3.39 (m, 1H) 1.82 (s, 3H), 1.79 (dt, *J* = 12.7, 3.5 Hz, 2H), 1.70 – 1.57 (m, 3H), 1.52 – 1.39 (m, 2H), 1.39 – 1.15 (m, 3H). Spectral data were in accordance with literature.[2]

1,1-dimethoxyhex-5-yn-2-one (AlkMGO-DMA)

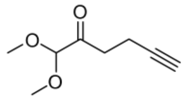

Adapted from the procedure reported by Cuvigny et al.[2] To a dry flask under nitrogen was added 1 M LDA in THF (17.2 mL, 17.2 mmol, 1.1 eq.). The flask was cooled down to -60 °C and the imine **3** (3.11 g, 15.6 mmol, 1 eq.) dissolved in dry THF (15 mL) was added dropwise. The mixture was allowed to stir for 1 h at -60 °C, then 80% propargyl bromide in toluene (1.8 mL, 16.38 mmol, 1.05 eq.) was added dropwise at -50°C. The solution was allowed to warm up to room temperature and left to stir for 48h. The reaction mixture was hydrolyzed with 2 M HCl (60 mL) and extracted with ethyl acetate. The aqueous layer was back extracted with ethyl acetate. The combined organic layers were washed with brine, dried over $MgSO_4$ and filtered. The solvent was removed *in vacuo*. The product was purified by column chromatography (0-20% ethyl acetate in pentane). The AlkMGO-DMA was obtained as a yellow oil (2.08 g, 13,3 mmol, 85% yield). ^{1}H NMR (400 MHz, Chloroform-d) δ 4.46 (s, 1H), 3.40 (s, 6H), 2.81 (t, J = 7.23 Hz, 2H), 2.44 (dt, J = 7.23, 2.6 Hz, 2H), 1.92 (t, J = 2.6 Hz, 1H). ^{13}C NMR (101 MHz, Chloroform-d) δ 203.6, 103.9, 83.0, 68.7, 54.8, 36.4, 12.3.

6-bromohexane-1,2-diol 5

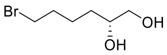

Substrate **4** (1 g, 6.13 mmol) was added to a solution of AD-mix-β (8.6 g) in *tert*-BuOH:DCM:water (1:1:1 v/v/v) and the mixture was left to stir at room temperature overnight. Subsequently the reaction was diluted with EtOAc and left to stir for an additional hour. Afterwards the organic layer was washed with water and brine, dried over $MgSO_4$ and filtered. The mixture was concentrated *in vacuo*. The obtain product **5** was as a brownish oil with 94% yield (1.14 g). ^{1}H NMR (400 MHz, Chloroform-*d*) δ 3.73 (td, *J* = 6.7, 2.9 Hz, 1H), 3.67 (dd, *J* = 10.9, 3.0 Hz, 1H), 3.48 – 3.44 (m,

Formatted: Font color: Auto

Formatted: Font color: Auto

Formatted: Font color: Auto

1H), 3.42 (t, *J* = 6.7 Hz, 2H), 2.11 (s, 1H), 1.89 (dqd, *J* = 8.3, 6.6, 2.0 Hz, 2H), 1.67 – 1.56 (m, 2H), 1.57 – 1.49 (m, 1H), 1.51 – 1.43 (m, 2H). ^{13}C NMR (101 MHz, Chloroform-*d*) 72.0, 66.7, 33.6, 32.6, 32.1, 24.2. Spectral data were in accordance with literature.[3]

Formatted: Font color: Auto

6-azidohexane-1,2-diol 6

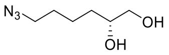

Substrate **5** (666 mg, 3.4 mmol) and sodium azide (220 mg, 3.4 mmol) were dissolved in DMF (17 mL) and stirred for overnight. The reaction mixture was poured onto ice-water and stirred for 30 min, extracted with ether and washed with brine. The organic layer was dried over $MgSO_4$, filtered and concentrated *in vacuo.* The product **6** was obtained as yellowish oil in 90% yield (484 mg). ^{1}H NMR (400 MHz, Chloroform-*d*) δ 3.77 – 3.63 (m, 2H), 3.49 – 3.41 (m, 1H), 3.29 (t, *J* = 6.7 Hz, 2H), 1.69 – 1.57 (m, 3H), 1.52 – 1.42 (m, 6H). ^{13}C NMR (101 MHz, Chloroform-*d*) 71.93, 66.73, 51.30, 32.56, 28.83, 22.77. Spectral data were in accordance with literature. [4]

Formatted: Font color: Auto

Formatted: Font color: Auto

Formatted: Font color: Auto

6-azido-2-hydroxyhexanal-1,1-dimethyl acetal 7

Formatted: Font color: Auto

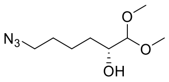

TEMPO (4 mg, 0.025 mmol) was added to a solution of substrate **6** (394 mg, 2.48 mmol) and trichloroisocyanuric acid (575 mg, 2.48 mmol) in ethyl acetate (7 mL) at 0 - 5 °C. The reaction mixture was left to stir at 5 °C for 15 minutes and then filtered through celite bed. Filtrate was washed with a mixture of saturated solution of $NaHCO_3$ and $Na_2S_2O_3$ (1:1). The aqueous layer was back extracted with ethyl acetate three times. The combined organic layers were dried over $MgSO_4$, filtered and evaporated under vacuum at 35 °C. The crude product was obtained in yield 175 mg (46%) and used directly in next reaction. ^{1}H NMR (400 MHz, Chloroform-*d*) δ 9.70 (s, 1H), 3.45 (t, *J* = 6.7 Hz, 1H), 3.37 – 3.29 (m, 12H), 1.72 – 1.63 (m, 3H), 1.63 – 1.55 (m, 2H).

Formatted: Font color: Auto

To a stirred suspension of azido-hydroxyhexanal (175 mg, 1.11 mmol), trimethyl orthoformate (0.183 mL, 1.67 mmol) and MS 3Å in MeOH (2.2 mL), *p*-toluenesulfonic acid (2 mg, 0.01 mmol) was slowly added and stirred at room temperature for 2 h. After the reaction was completed, Et_2O and sat. aq. $NaHCO_3$ were added. The organic layer was separated and the aqueous layer was extracted with Et_2O three times. The combined organic layers were washed with brine, and dried over anhydrous $MgSO_4$. The solvent was removed under reduced pressure to give azidohexanol dimethyl acetal **7** as a colorless oil (180 mg, 80% yield). ^{1}H NMR (400 MHz, Chloroform-*d*) δ 4.12 (d, *J* = 6.1 Hz, 1H), 3.59 (t, *J* = 7.7 Hz, 1H), 3.46 (s, 3H), 3.43 (s, 3H), 3.30 (td, *J* = 7.5, 6.7, 4.6 Hz, 3H), 1.63 (td, *J* = 6.7, 2.5 Hz, 4H), 1.49 – 1.41 (m, 2H). ^{13}C NMR(101 MHz, Chloroform-*d*) δ 106.9, 70.9, 55.2, 55.1, 51.4, 31.2, 28.9, 22.7. HRMS (ESI-) *m/z* calculated for the aldehyde $[C_6H_{10}N_3O_2]^-$ 156.07675, found 156.07780.

Formatted: Font color: Auto

Formatted: Font color: Auto

Formatted: Font color: Auto

6-azido-1,1-dimethoxyhexan-2-one (AzBGO-DMA)

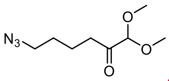

Azidohexanol dimethyl acetal **7** (50 mg, 0.25 mmol), Dess-martin periodinane (104 mg, 0.25 mmol), $NaHCO_3$ (41.3 mg, 0.5 mmol) were disolved in dry DCM (2 mL), cooled to 0 °C and left to stir for 3 hours under N_2. The reaction was controlled via TLC (mobile phase: 10% EtOAc/pentane eluent). The reaction was left to stir overnight. The crude prouct was diluted with Et_2O and then a mixture 1:1:1 of sat. aq. $Na_2S_2O_3$, sat. aq. $NaHCO_3$, water was slowly added to the solution and left to vigorously stir for 1 hour. Then organic and water layers were separated and water was extracted with Et_2O twice. The combined organic layers were dried over $MgSO_4$, filtered and concentrated *in vacuo*. The crude product was purified by automated flash chromatography column (linear gradient: 0% to 10% EtOAc in pentane in 5 min., then isocratic elution was performed with 10% EtOAc in pentane) yielding in AzBGO-dimethyl acetal in 46% yield (22.5 mg). ^{1}H NMR (400 MHz, Chloroform-*d*) δ 4.44 (s, 1H), 3.42 (s, 6H), 3.28 (t, *J* = 6.6 Hz, 2H), 2.60 (t, *J* = 7.0 Hz, 2H), 1.70 – 1.59 (m, 6H). ^{13}C NMR (101 MHz, Chloroform-*d*) δ 205.1, 104.3, 54.9, 51.2, 36.4, 28.3, 20.1. The HRMS could not be found. Fragments of probes were identified. HRMS (ESI-) *m/z* calculated for the pyruvate methyl ester $[C_7H_{10}N_3O_3]^-$ 184.07276, found 184.07256; *m/z* calculated for pyruvate $[C_6H_8N_3O_3]^-$ 170.05711, found 170.05731; *m/z* calculated for AzBGO $[C_6H_8N_3O_2]^-$ 154.06110, found 154.06230.

1,2-dimethoxy-4-nitro-5-vinylbenzene 9

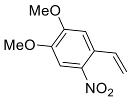

Adapted from the procedure reported by Marinzi et al.[6] To a solution of methyltriphenylphosphonium bromide (8.5 g, 24 mmol) in THF (17 mL) was added sodium bis(trimethylsilyl)amid (2 M in THF, 8.9 mL, 18 mmol) and the mixture was left to stir at 0 °C under N_2 atmosphere for 30 minutes. Subsequently, a solution of 4,5-dimethoxy-2-nitrobenzaldehyde **8** (2.5 g, 12 mmol) in THF (24 mL) was added. The reaction mixture was left to stir at 0 °C for 1h and then over night at room temperature. The next day, the reaction mixture was diluted with DCM and the organic layer was washed with water. The aqueous layer was back extracted three times with DCM. The organic layers were combined, dried over $MgSO_4$ and filtered. The solvent was reduced *in vacuo*. The crude product was absorbed on celite and purified by automated flash column chromatography (linear gradient: 0 to 10% EtOAc in pentane in 15 min.) resulting in final product **9** as a yellow solid (1.78 g, 72 % yield). ^{1}H NMR (400 MHz, Chloroform-*d*) δ 7.59 (s, 1H), 7.31 (dd, *J* = 17.3, 10.9 Hz, 1H), 6.97 (s, 1H), 5.64 (d, *J* = 17.3 Hz, 1H), 5.44 (d, *J* = 10.9 Hz, 1H), 4.00 (s, 3H), 3.95 (s, 3H). ^{13}C NMR (101 MHz,

Formatted: Font color: Auto

Formatted: Font color: Auto

Formatted: Font color: Auto

Formatted: Font color: Auto

Formatted: Font color: Auto

Chloroform-*d*) δ 153.1, 148.3, 133.5, 133.5, 128.6, 117.4, 109.6, 107.4, 56.3. Spectral data were in accordance with literature.[6]

1-(4,5-dimethoxy-2-nitrophenyl)ethane-1,2-diol (PC1)

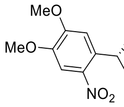

To a solution of AD-mix-α (3.85 g) in *tert*-BuOH:DCM:water (1:1:1 v/v/v) substrate **9** (0.57 g, 2.73 mmol) was added and the mixture was left to stir at room temperature overnight. Subsequently the reaction was diluted with EtOAc and left to stir for an additional hour. Afterwards the organic layer was washed with water and brine, dried over $MgSO_4$ and filtered. The mixture was concentrated *in vacuo*, absorbed on celite and purified by automated flash column chromatography (linear gradient: 0 to 40% EtOAc in pentane in 20 min.). The photocage **PC1** was obtained as a yellowish solid (0.57 g, 86% yield). ^{1}H NMR (400 MHz, Chloroform-*d*) δ 7.63 (s, 1H), 7.35 (s, 1H), 4.05 (dd, *J* = 10.5, 2.7 Hz, 1H), 4.00 (s, 3H), 3.95 (s, 3H), 3.59 (dd, *J* = 10.9, 7.1 Hz, 2H) ^{13}C NMR (101 MHz, Chloroform-*d*) δ 142.2, 126.6, 119.9, 117.8, 112.6, 109.8, 107.2, 56.4, 51.4. Spectral data were in accordance with literature.[6]

1-(4,5-dimethoxy-2-nitrophenyl)-2,2-dimethylpropane-1,3-diol PC2

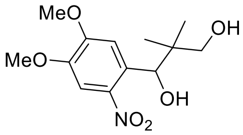

Adapted from the procedure reported by Mase et al.[7] 6-nitroveratralaldehyde **8** (1 g, 4.74 mmol, 1 eq.) was dissolved in anhydrous DMSO (5 mL) and isobutyraldehyde (1.2 mL, 5.67 mmol, 1.2 eq.) was added. To the mixture 2.3 M acetic acid in DMSO (0.5 mL, 1.16 mmol, 0.24 eq.) and 0.48 M pyrrolidine in DMSO (0.5 mL, 0.24 mmol, 0.05 eq.) were added. After stirring for 2 h at room temperature, the reaction was checked by TLC (40% ethyl acetate in pentane) and additional isobutyraldehyde (0.21 mL, 2.37 mmol, 0.5 eq.) was added to the mixture. The solution was extracted 2 times with ethyl acetate. The combined organic layers were washed with brine, dried over $MgSO_4$ and filtered. The solvent was removed *in vacuo*. The product was purified by column chromatography (0-40% ethyl acetate in pentane). An inseparable mixture (0.99 g) of the aldol product **10** and 6-nitroveratralaldehyde **8** was isolated in 1:0.3 ratio (the corrected yield for **10** was 2.86 mmol, 0.81 g, 60% yield), ^{1}H NMR (400 MHz, chloroform-d) δ 9.63 (s, 1H), 7.53 (s, 1H), 7.20 (s, 1H), 6.07 (s, 1H), 3.98

Formatted: Font color: Auto
Formatted: Font color: Auto
Formatted: Font color: Auto
Formatted: Font color: Auto
Formatted: Font color: Auto
Formatted: Font color: Auto

(s, 3H), 3.95 (s, 3H), 1.08 (s, 3H), 1.00 (s, 3H). Therefore, this mixture was subjected to the reduction. The mixture was dissolved in methanol (15 mL). Sodium borohydride (0.28 g, 7.4 mmol, 2.75 eq.) was added slowly under an ice bath. The reaction was left to stir for 1 hour and quenched with 2 M HCl (8 mL). Methanol was removed *in vacuo*. The solution was extracted two times with ethyl acetate. The combined organic layers were washed with brine, dried over $MgSO_4$ and filtered. The solvent was removed *in vacuo*. The product was purified by column chromatography (0-40% ethyl acetate in pentane). The photocage **PC2** was obtained as a yellow viscous oil (0.59 g, 2.07 mmol, 44% over two steps). ^{1}H NMR (400 MHz, Chloroform-d) δ 7.43 (s, 1H), 7.25 (s, 1H), 5.86 (s, 1H), 3.96 (s, 3H), 3.92 (s, 3H), 3.57 (s, 2H), 0.83 (s, 3H), 0.77 (s, 3H). ^{13}C NMR (101 MHz, Chloroform-d) δ 152.3, 147.7, 141.3, 131.0, 111.0, 107.4, 74.0, 72.6, 56.2, 40.3, 21.9, 18.6. HRMS (ESI+) *m/z* calculated for $[C_{13}H_{19}NO_6Na]^+$ 308.1105, found 308.1107

1,1-dimethoxyhex-5-yn-2-ol 11

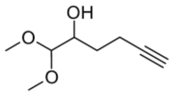

AlkMGO-DMA (0.62 g, 3.977 mmol, 1 eq.) was dissolved in methanol (10 mL). Sodium borohydride (0.15 g, 3.97 mmol, 1 eq.) was added slowly under an ice bath. The reaction was left to stir for 1 hour and quenched with 2 M HCl (5 mL). Methanol was removed *in vacuo*. The solution was extracted twice with ethyl acetate. The combined organic layers were washed with brine, dried over $MgSO_4$ and filtered. The solvent was removed *in vacuo*. Compound **11** was obtained as a clear liquid (0.52 g, 3.3 mmol, 83% yield). ^{1}H NMR (400 MHz, Chloroform-d) δ 4.16 (d, J = 6.0 Hz, 1H), 3.75 (ddd, J = 9.3, 6.0, 3.0 Hz, 1H), 3.46 (s, 3H), 3.43 (s, 3H), 2.41 – 2.34 (m, 2H), 1.96 (t, J = 2.6 Hz, 1H), 1.89 – 1.78 (m, 1H), 1.68-1.58 (m, 1H). Spectral data were in accordance with literature.[8]

Formatted: Font color: Auto

Formatted: Font color: Auto

5-azido-1-(4-(4,5-dimethoxy-2-nitrophenyl)-1,3-dioxolan-2-yl)pentan-1-one AzBGO-PC1

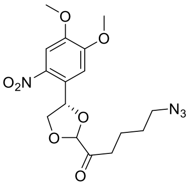

Formatted: Font color: Auto

Dimethyl acetal **7** (120 mg, 0.5 mmol) was dissolved in DCM (2 mL) and camphorsulfonic acid (86 mg, 0.4 mmol) was added. Subsequently, **PC1** was added to the mixture and the reaction was left to stir in dark over night at room temperature. The next day, the reaction was quenched with a sat. aq. solution of $NaHCO_3$ and the mixture was extracted with DCM. The organic layer was dried over $MgSO_4$ and filtered. The solvent was removed *in vacuo* and the crude product

was purified by automated flash column chromatography (linear gradient: 0 to 30% EtOAc in pentane in 15 min. and then isocratic elution in 30% EtOAc in pentane) to give the alcohol intermediate **12** as a mixture of diastereomers isomers in 67 % yield (126 mg). ^{1}H NMR (400 MHz, Chloroform-*d*) δ 7.74 (s, 1H), 7.70 (s, 1H), 5.71 (dd, *J* = 7.8, 4.1 Hz, 1H), 4.98 (dd, *J* = 5.4, 2.5 Hz, 1H), 4.55 (ddd, *J* = 8.7, 7.7, 1.0 Hz, 1H), 4.00 (s, 3H), 3.95 (s, 6H), 3.90 (dd, *J* = 8.6, 4.4 Hz, 2H), 1.76 – 1.62 (m, 17H). ^{13}C NMR (101 MHz, Chloroform-*d*) δ 175.5, 154.0, 147.9, 133.6, 109.4, 107.8, 105.4, 74.1, 70.1, 56.4, 52.6, 51.2, 33.7, 28.5, 22.1. The substrate **12** (32 mg, 0.08 mmol), Dess-martin periodinane (36 mg, 0.08 mmol) and $NaHCO_3$ (14 mg, 0.17 mmol) were disolved in dry DCM (1.7 mL), cooled to 0 °C and left to stir overnight under N_2. Subsequently, the crude product was dissolved in Et_2O and then a mixture 1:1:1 of sat. aq. $Na_2S_2O_3$, sat. aq. $NaHCO_3$, water was slowly added and left to vigorously stir for 1 hour. Then, the organic layer and the water layer were separated and the water layer was washed with Et_2O twice. The combined organic layers were dried over $MgSO_4$, filtered and the solvent was removed *in vacuo*. The crude product was purified by automated flash column chromatography (linear gradient: 0 to 20% EtOAc in pentane in 15 min. and then isocratic elution was performed in 20% EtOAc in pentane) to give a cis-trans mixture of AzBGO-PC1, as a yellow solid with yield of 62% (20 mg). ^{1}H NMR (400 MHz, Chloroform-*d*) δ 7.76 – 7.64 (m, 2H), 4.80 – 4.49 (m, 1H), 4.00 (dd, *J* = 5.9, 3.3 Hz, 3H), 3.96 (d, *J* = 2.8 Hz, 3H), 3.31 (td, *J* = 6.6, 3.4 Hz, 2H), 2.80 – 2.63 (m, 1H), 1.75 (m, 4H), 1.68 – 1.60 (m, 4H). ^{13}C NMR (101 MHz, Chloroform-*d*) δ 204.0, 148.1, 109.3, 108.2, 107.9, 105.4, 102.9, 102.3, 56.4, 37.7, 31.9, 29.7, 22.7, 20.1, 14.1. HRMS (ESI+) *m/z* calculated for $[C_{16}H_{19}N_4O_7]^-$ 379.1259, found 379.1258

Cis and trans 1-(4-(4,5-dimethoxy-2-nitrophenyl)-1,3-dioxolan-2-yl)pent-4-yn-1-one (AlkMGO-PC1 cis & AlkMGO-PC1 trans)

Adapted from the procedure reported by Thuaud et al.[9] Racemic dimethoxy acetal **11** (0.31 g, 1.63 mmol, 1 eq.) was dissolved in DCM (4 mL) and camphorsulfonic acid (0.3 g, 1,3 mmol, 0.8 eq.) was added. Subsequently, enantiomerical pure **PC1** (0.44g, 1.8 mmol, 1.1 eq.)) was added to the mixture and the reaction was left to stir under nitrogen in the dark over 48 hours at room temperature. Afterwards, the reaction was checked by TLC (mobile phase 40% ethyl acetate in pentane) and ^{1}H NMR. The reaction was quenched with a sat. aq. solution of $NaHCO_3$ and the mixture was extracted twice with ethyl acetate. The combined organic layers were washed with brine, dried over $MgSO_4$ and filtered. The solvent was removed *in vacuo* and the crude product was purified by column chromatography (0-40% ethyl acetate in pentane) to give two isomers of compound **13**. Upper isomer: 190 mg, 0.56 mmol. ^{1}H NMR (400 MHz, chloroform-d) δ 7.70 (s, 1H), 7.66 (s, 1H), 5.68 (dd, *J* = 7.7, 4.1 Hz, 1H), 4.98 (d, *J* = 2.3 Hz, 1H), 4.51 (dt, *J* = 8.3, 1.2 Hz, 1H), 3.97 (s, 3H), 3.92 (s, 3H), 2.47 – 2.40 (m, 2H), 1.97 (t, *J* = 2.7 Hz, 1H), 1.94 – 1.82 (m, 2H). Lower fraction contained trace amounts of the upper

Formatted: Font color: Auto

Formatted: Font color: Auto

Formatted: Font color: Auto

Formatted: Font color: Auto

Formatted: Font color: Auto

Formatted: Font color: Auto

Formatted: Font color: Auto

isomer: 130 mg, 0.39 mmol 1H NMR (400 MHz, chloroform-d) δ 7.63 (d, J = 3.2 Hz, 1H), 7.19 (d, J = 4.8 Hz, 1H), 5.70 (dt, J = 8.8, 6.4 Hz, 1H), 5.14 (dd, J = 6.6, 4.3 Hz, 1H), 4.71 (ddd, J = 8.6, 6.6, 1.9 Hz, 1H), 3.94 (d, J = 2.1 Hz, 3H), 3.90 (s, 3H), 3.87 – 3.76 (m, 1H), 3.61 (dt, J = 8.6, 6.2 Hz, 1H), 2.38 (dtt, J = 12.9, 4.9, 2.5 Hz, 2H), 1.94 (td, J = 2.8, 1.7 Hz, 1H), 1.86 – 1.63 (m, 2H). The combined yield was 58%. The products are at this stage epimeric mixtures at the alcohol. In order to simplify the analysis of these products, the alcohol intermediate was oxidized prior to further analysis. This step converts the products into a single diastereomer.

Oxidation of the upper isomer:

Formatted: Font color: Auto

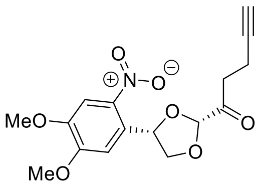

Adapted from the procedure reported by Zheng et al. [7] The cyclic acetal (190 mg, 0.56 mmol, 1 eq.), Dess-martin periodinane (239 mg, 0.56 mmol, 1 eq.) and $NaHCO_3$ (101 mg, 1.20 mmol, 2.1 eq.) were dissolved in dry DCM (12 mL), cooled to 0 °C and left to stir for 48 hours under nitrogen. The reaction was monitored by TLC (40% ethyl acetate in pentane) and ^{1}H NMR. Afterwards, the crude product was dissolved in diethyl ether and then a mixture 1:1:1 of 1M Na_2SO_3, sat. aq. $NaHCO_3$ and water was slowly added and left to vigorously stir for 30 minutes. Then, the organic layer and the water layer were separated, and the water layer was back extracted once. The combined organic layers were washed with brine, dried over $MgSO_4$ and filtered. The solvent was removed in vacuo. The crude product was purified by column chromatography (0-40% ethyl acetate in) to give final **AlkMGO-PC1-cis** as a pale yellow solid in 64% yield (120 mg, 0.36 mmol). ^{1}H NMR (400 MHz, chloroform-d) δ 7.70 (s, 1H), 7.61 (s, 1H), 5.80 (dd, *J* = 7.0, 5.5 Hz, 1H), 5.32 (s, 1H), 4.65 (dd, *J* = 8.7, 7.0 Hz, 1H), 4.03 (s, 3H), 3.95 (s, 3H), 3.84 (dd, *J* = 8.7, 5.5 Hz, 1H), 3.07 – 2.84 (m, 2H), 2.53 (tt, *J* = 7.3, 2.8 Hz, 2H), 1.95 (t, *J* = 2.7 Hz, 1H). ^{13}C NMR (101 MHz, chloroform-d) δ 202.6, 154.2, 148.3, 139.0, 131.6, 109.3, 108.0, 102.4, 82.6, 76.0, 72.7, 69.2, 56.8, 56.5, 37.3, 12.5. HRMS (ESI+) *m/z* calculated for $[C_{16}H_{17}NO_7Na]^+$ 358.0897, found 358.0901

Formatted: Font color: Auto

Formatted: Font color: Auto

Oxidation of the lower isomer:

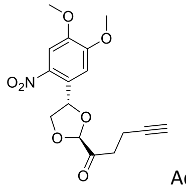

Adapted from the procedure reported by Zheng et al.[10] The lower isomer of cyclic acetal **13** (130 mg, 0.385 mmol, 1 eq.), Dess-martin periodinane (163 mg, 0.385 mmol, 1 eq.) and $NaHCO_3$ (69 mg, 0.82 mmol, 2.1 eq.) were dissolved in dry DCM (4 mL), cooled to 0 °C and left to stir for 48 hours under nitrogen. The reaction was monitored by TLC (40% ethyl acetate in pentane) and ^{1}H NMR. Afterwards, the crude product was dissolved in diethyl ether and then a mixture 1:1:1 of 1 M Na_2SO_3, sat. aq. $NaHCO_3$ and water was slowly added and left to vigorously stir for 30 minutes. Then, the organic layer and the water layer were separated, and the water layer was back extracted once. The combined organic layers were washed with brine, dried over $MgSO_4$ and filtered. The solvent was removed *in vacuo*. The crude product was purified by column chromatography (0-40% ethyl acetate in) to give final **AlkMGO-PC1-trans** as a pale yellow solid in 93% yield (120 mg, 0.36 mmol). ^{1}H NMR (400 MHz, Chloroform-d) δ 7.70 (s, 1H), 7.66 (s, 1H), 5.68 (dd, J = 7.7, 4.1 Hz, 1H), 4.98 (d, J = 2.3 Hz, 1H), 4.51 (dt, J = 8.3, 1.2 Hz, 1H), 3.97 (s, 3H), 3.92 (s, 3H), 2.47 – 2.40 (m, 2H), 1.97 (t, J = 2.7 Hz, 1H), 1.94 – 1.82 (m, 2H). ^{13}C NMR (101 MHz, Chloroform-d) δ 203.3, 154.1, 148.3, 131.2, 109.1, 108.2, 107.9, 102.75, 102.28, 75.7, 56.7, 56.4. HRMS (ESI+) *m/z* calculated for $[C_{16}H_{17}NO_7Na]^+$ 358.0897, found 358.0897.

Formatted: Font color: Auto

Formatted: Font color: Auto

1-(4-(4,5-dimethoxy-2-nitrophenyl)-5,5-dimethyl-1,3-dioxan-2-yl)pent-4-yn-1-one AlkMGO-PC2 (*rac*)

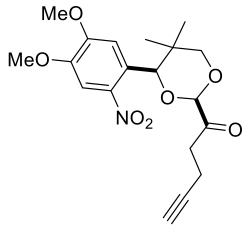

Adapted from the procedure reported by Thuaud et al.[9] Dimethyl acetal **11** (0.057 g, 0.36 mmol, 1 eq.) was dissolved in DCM (2 mL) and camphorsulfonic acid (0.158 g, 0.68 mmol, 1.9 eq.) was added. Subsequently, photocage PC2 (0.123 g, 0.43 mmol, 1.2 eq.) was added to the mixture and the reaction was left to stir under nitrogen in the dark over 48 hours at room temperature. Afterwards, the reaction was checked by TLC (mobile phase 40% ethyl acetate in pentane). The TLC indicated incomplete conversion. The DCM was evaporated and DMF (2 mL) was added to the mixture. The flask was stirred on the rotary evaporator at 200 millibar and 54 °C water

bath for 1h 30 min. After checking the conversion by TLC and ^{1}H NMR, the reaction was quenched with a sat. aq. solution of $NaHCO_3$ and the mixture was extracted twice with ethyl acetate. The combined organic layers were washed with brine, dried over $MgSO_4$ and filtered. The solvent was removed *in vacuo* and the crude product was purified by column chromatography (0-40% ethyl acetate in pentane) to give the title compound **13** as yellow crystals in 57% yield (0.08 g, 0.2 mmol).^{1}H NMR (400 MHz, Chloroform-d) δ 7.49 (s, 1H), 7.10 (s, 1H), 5.73 (d, J = 1.2 Hz, 1H), 4.71 (dd, J = 6.2, 4.6 Hz, 1H), 3.96 (d, J=2.6, 3H), 3.93 (s, 3H), 3.70 (m, 2H), 2,40 (m, 2H), 1.95 (m, 1H), 1.83 (m, 2H), 0.90 (d, J = 1.43 Hz, 3H), 0.62 (s, 3H).

Oxidation of the alcohol intermediate

Adapted from the procedure reported by Zheng et al.[10] Acetal **13** (78 mg, 0.2 mmol, 1 eq.), Dess-martin periodinane (87 mg, 0.2mmol, 1 eq.) and $NaHCO_3$ (37 mg, 0.43 mmol, 2.1 eq.) were dissolved in dry DCM (4 mL), cooled to 0 °C and left to stir for 48 hours under nitrogen. The reaction was monitored by TLC (405 ethyl acetate in pentane) and ^{1}H NMR. Afterwards, the crude product was dissolved in diethyl ether and then a mixture 1:1:1 of 1 M Na_2SO_3, sat. aq. $NaHCO_3$ and water was slowly added and left to vigorously stir for 30 minutes. Then, the organic layer and the water layer were separated, and the water layer was back extracted once. The combined organic layers were washed with brine, dried over $MgSO_4$ and filtered. The solvent was removed *in vacuo*. The crude product was purified by column chromatography (0-40% ethyl acetate in) to give the racemic final compound AlkMGO-PC2 as a yellow solid in 82% yield (63 mg, 0.17 mmol). ^{1}H NMR (400 MHz, Chloroform-d) δ 7.50 (s, 1H), 7.09 (s, 1H), 5.83 (s, 1H), 5.02 (s, 1H), 3.95 (s, 3H), 3.92 (s, 3H), 3.79 (d, *J* = 11.2 Hz, 1H), 3.73 (d, *J* = 11.3 Hz, 1H), (m, 2H), 2.97 (dt, *J* =7.43, 2Hz, 2H), 2.50 (dt, *J* = 7.47, 2.7 Hz, 2H), 1.93 (t, *J* = 2.7, 1H), 0.94 (s, 3H), 0.64 (s, 3H). 13C NMR (101 MHz, Chloroform-d) δ 200.8, 152.5, 148.2, 128.2, 126.4, 111.4, 107.8, 100.7, 80.2, 78.6, 69.0, 56.5, 56.4, 36.8, 36.2, 20.9, 19.6, 12.5. HRMS (ESI+) *m/z* calculated for $[C_{19}H_{23}NO_7Na]^+$ 400.1367, found 400.1371

Formatted: Font color: Auto

Formatted: Font color: Auto

Formatted: Font color: Auto

Uncaging rate measurement

Procedure: Each of the probes dissolved in DMSO with the following concentrations: 2.5 mM, 1.0 mM, and 0.5 mM was diluted 10x with HEPES buffer to yield the final concentrations of: 0.25 mM, 0.1 mM, and 0.05 mM. The samples (100 µL) were irradiated with 365 nm UV light in a 96-well plate. The absorbance in the 300 to 400 nm range was measured with a UV-vis spectrometer (a plate reader). The measurements were executed after 0, 1, 5, 10, 30, 45, and 60 min after the start of irradiation.

General biochemical procedures

Preparation of stock solutions of the probes: The photo-caged probes AzBGO-PC1, AlkMGO-PC1-cis, AlkMGO-PC1-trans, and AlkMGO-PC2 were dissolved in DMSO in Eppendorf tubes to obtain the concentration of 100 mM, 20 mM, 10 mM, 5mM, 2.5 mM, 1 mM, 0.5 mM and stored at - 15°C.

Stock solution of other reagents: All click reagents were bought from commercial vendors. Stock solutions of Cy5-azide (3 mM), Cy5-alkyne (3 mM), $CuSO_4.5H_2O$/THPTA (20 mM) and sodium ascorbate (10 mM) were prepared by dissolving the compounds in water. The stock solution of azide-PEG3-biotin (20 mM) was prepared dissolving the compound in DMSO. All stock solutions were stored at - 15°C.

Protein precipitation adapted from the procedure reported by Shahinuzzaman et al. [11]: Cold methanol and chloroform were used to facilitate precipitation of the protein. The protein sample (100 µL) was pipetted into Eppendorf tubes. Methanol (400 µL) was added and the tubes were vortexed well, then spun briefly to collect the sample. Chloroform (100 µL) was added and the tubes were vortexed well, then spun briefly to collect the sample. Demi water (300 µL) was added and vortexed well. The tubes were centrifuged at 13,000 rpm for 5 minutes. The upper layer was removed without disturbing the interface. Methanol (300 µL) was added and the tubes were vortexed well, then centrifuged at 13,000 rpm for 5 minutes to pellet the proteins. The supernatant was removed, and the pellets were left to air dry.

SDS-PAGE labeling of bovine serum albumin*:*

Labeling of BSA with ***AlkMGO-PC1 cis****,* ***AlkMGO-PC1 trans*** *and* ***AlkMGO-PC2*** *in the presence and absence of UV light (Detection with Cy5, Figure S6B)*: The probes **AlkMGO-PC1 cis**, **AlkMGO-PC1 trans**, **AlkMGO-PC2** and **AzBGO-PC1** with the final concentrations: 0.2 mM (1 µL of 2 mM stock solution) were added to BSA (1 mg/mL) in PBS (9 µL, pH 7.4). The samples were then either exposed to UV light of 365 nm wavelength for 1 h or kept in the dark for the same amount of time. The protein solution was then denatured by the addition of SDS (1 µL, 10% w/v) and heated at 100 °C for 10 min. This was followed by a click reaction with click mix: Cy5-azide or Cy5-alkyne (1 µL of a 3 mM stock), $CuSO_4$/THPTA (1 µL of a 20 mM stock), sodium ascorbate (1 µL of a 10 mM stock) for 2 h. The samples of probe **AzBGO-PC1** were subjected to Click reaction with click mix: Cy5-alkyne 1 µL, 3 mM), $CuSO_4$ / THPTA (1 µL, 20 mM), sodium ascorbate (1 µL, 10 mM) for 2 h in the dark The reaction was quenched with SDS-PAGE sample buffer and loaded on a SDS gel (12%). The gel was scanned on Typhoon scanner FLA 9500 using the Cy5 setting.

SDS-page labeling of *E. coli* lysate

E. coli labeling with AlkMGO derivatives (Figure S6C): E. coli lysate (2 mg/mL) in phosphate buffer (pH 7.4) was divided into 6 Eppendorf tubes in 90 µL and 3 Eppendorf tubes in 18 µl. As a negative control, 2 µL of DMSO was added to the lysate.

10 µL of 2.5 mM stock solution of the probes **AlkMGO-PC1 cis**, **AlkMGO-PC1 trans** or **AlkMGO-PC2** was added into the lysate in 2 Eppendorf tubes each obtaining the final concentration of the reagent 0.25 mM. 2 µl 2.5 mM stock solution of the probe **AzBGO-PC1** were added to 2 Eppendorf tubes. One Eppendorf tube containing each probe was then exposed to UV light of 365 nm wavelength for 1h. Subsequently, samples containing **AlkMGO-PC1 cis**, **AlkMGO-PC1 trans** or **AlkMGO-PC2** were subjected to methanol: chloroform protein precipitation. Afterwards, the dry pellets were resuspended in 100 µl PBS. The protein solution was then denatured by the addition of 10 µL 10% SDS solution (1% final volume) and heated at 100°C for 10 min. 20 µL from each sample was used in the next step. The samples of probes **AlkMGO-PC1 cis**, **AlkMGO-PC1 trans** or **AlkMGO-PC2** were subjected to Click reaction with click mix: Cy5-azide (2 µL, 3 mM), $CuSO_4$/THPTA (2 µL, 20 mM), sodium ascorbate (2 µL, 10 mM) for 2 h in the dark. The samples of probe **AzBGO-PC1** were subjected to Click reaction with click mix: Cy5-alkyne (2 µL, 3 mM), $CuSO_4$/THPTA (2 µL, 20 mM), sodium ascorbate (2 µL, 10 mM) for 2 h in the dark. The reaction was quenched with SDS PAGE sample buffer and loaded to SDS gel (12.5%). The gel was scanned on Typhoon scanner FLA 9500 using the Cy5 setting.

Labeling with probes in the presence or absence of UV light (Detection by western blotting, Figure S7B):

To *E. coli* lysate (2 mg/mL) in phosphate buffer (pH 7.4, 90 µL) were added the probes **AlkMGO-PC1 cis**, **AlkMGO-PC1 trans**, **AlkMGO-PC2** (10 µL of 2.5 mM stock solution) or the chemically activated **AlkMGO-DMA** (10 µL of 2.5 mM stock solution). One Eppendorf tube containing probe-protein solution was then exposed to UV light of 365 nm wavelength for 1 h. The other tube was kept in the dark. Subsequently, the excess was removed by washing the protein solution with diethyl ether (100 µL, twice). The organic layer was removed and discarded. To the remaining aqueous layer was added SDS (10 µL, 10% w/v) and the mixture was heated at 100 °C for 10 min. 10 µL from each sample was used in the next step. The samples were subjected to Click reaction with click mix: biotinPEG3-azide (2 µL, 20 mM), $CuSO_4$/THPTA (2 µL, 20 mM), sodium ascorbate (2 µL, 20 mM) overnight. The reaction was quenched with SDS-PAGE sample buffer and loaded onto a 12.5% SDS-PAGE gel. The proteins were transferred to a PVDF membrane with a Mini-Trans blot system for wet blotting (Bio-Rad) using wet blotting with a Tris/glycine buffer containing 20% EtOH (v/v) and according the manufacturers protocol. The membrane was blocked with BSA (0.5% in TBS-T) for 1 hour, and probed with Strp-HRP (1:12,500, 0.5% in TBS-T). The membranes were washed with TBS-T (3x) and TBS. The biotinylated proteins were visualized with Clarity Western ECL+ substrate on a ChemiDoc XRS (Bio-Rad) according

to the manufacturer's protocol. Afterwards the blots were stained with ReadyBlue Protein Gel Stain (purchased from Sigma-Aldrich).

Western blotting of *E. coli* lysate with AzBGO-PC1

The cell lysate samples were labeled according to the optimized conditions. The samples were loaded on a 12% SDS-PAGE gel and left to run at 150 V for 1 h 20 min. The proteins were transferred onto a nitrocellulose membrane using wet blotting with a Tris/glycine buffer containing 20% EtOH (v/v), according to the manufacturer's protocol. The proteins were transferred at 70 V for 1 h 20 min. The membrane was subsequently blocked with 1% BSA in TBS containing a Tween (0.1%, v/v) buffer for 45 min and then washed with TBS-Tween twice for 10 minutes. The blot was probed with Strp-DyLight650 (1:12,500) in 1% BSA TBS-Tween for 45 minutes in the dark. The membrane was washed twice with TBS containing Tween (0.1%) for 10 minutes, followed by washing with TBS buffer twice for 5 minutes. The membrane was scanned on ad Typhoon scanner FLA 9500 using the Cy5 setting.

References

[1] R. S. Michalak, R. Galante, D. M. Blum, J. Blum, L. Routel, H. Durutlic, C. Guinosso, J. Considine, K. A. M. Kremer, *Process for Preparing Beta-Lactamase Inhibitors*, **2007**, US2007/0149499 A1.

[2] T. Cuvigny, H. Normant, *Synthesis (Stuttg).* **1977**, *1977*, 198–200.

[3] P. Allevi, M. Anastasia, F. Cajone, *Chem. Phys. Lipids* **1999**, *100*, 89–99.

[4] H. Takahata, M. Kubota, S. Takahashi, T. Momose, *Tetrahedron: Asymmetry* **1996**, *7*, 3047–3054.

[5] N. R. Patel, C. C. Nawrat, M. Mclaughlin, Y. Xu, M. A. Huffman, H. Yang, H. Li, A. M. Whittaker, T. Andreani, F. Ois, et al., *Org. Lett* **2020**, *22*, 4659–4664.

[6] C. Marinzi, J. Offer, R. Longhi, P. E. Dawson, *Bioorg. Med. Chem.* **2004**, *12*, 2749–2757.

[7] N. Mase, F. Tanaka, C. F. Barbas, *Org. Lett.* **2003**, *5*, 4369–4372.

[8] C. Sibbersen, A.-M. Schou Oxvig, S. Bisgaard Olesen, C. B. Nielsen, J. J. Galligan, K. A. Jørgensen, J. Palmfeldt, M. Johannsen, *ACS Chem. Biol.* **2018**, *13*, 3294–3305.

[9] F. Thuaud, F. Rohrbacher, A. Zwicky, J. W. Bode, *Helv. Chim. Acta* **2016**, *99*, 868–894.

[10] Q. Zheng, I. Maksimovic, A. Upad, D. Guber, Y. David, *J. Org. Chem.* **2020**, *85*, 1691–1697.

[11] A. D. A. Shahinuzzaman, J. K. Chakrabarty, Z. Fang, D. Smith, A. H. M. Kamal, S. M.

Chowdhury, *J. Sep. Sci.* **2020**, *43*, 2125–2132.

Spectral data

^{1}H NMR of *N*-cyclohexyl-1,1-dimethoxypropan-2-imine 3

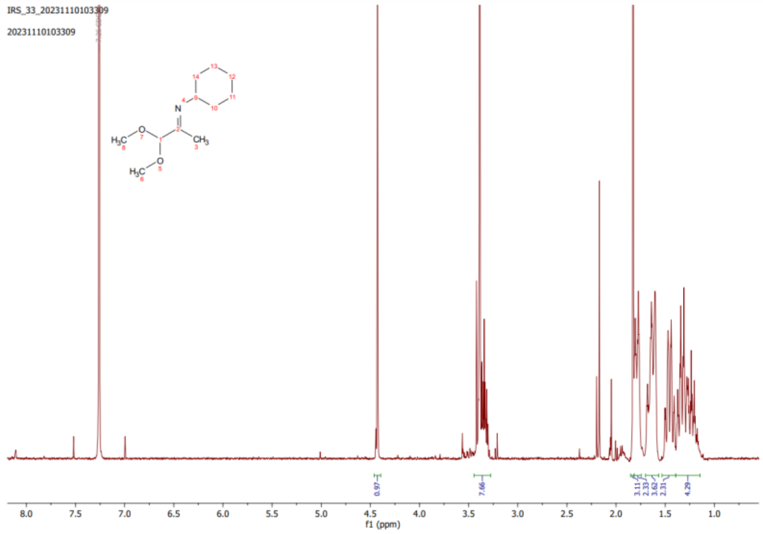

^{1}H NMR of AlkMGO-DMA

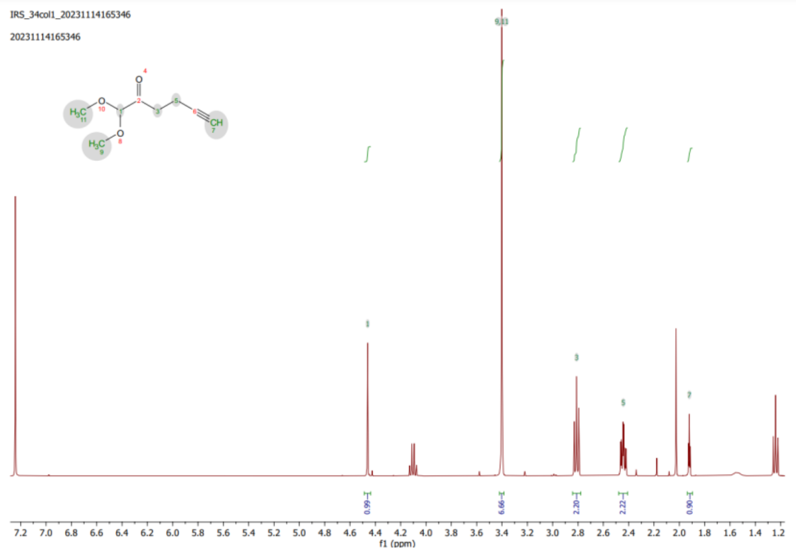

^{13}C NMR of AlkMGO-DMA

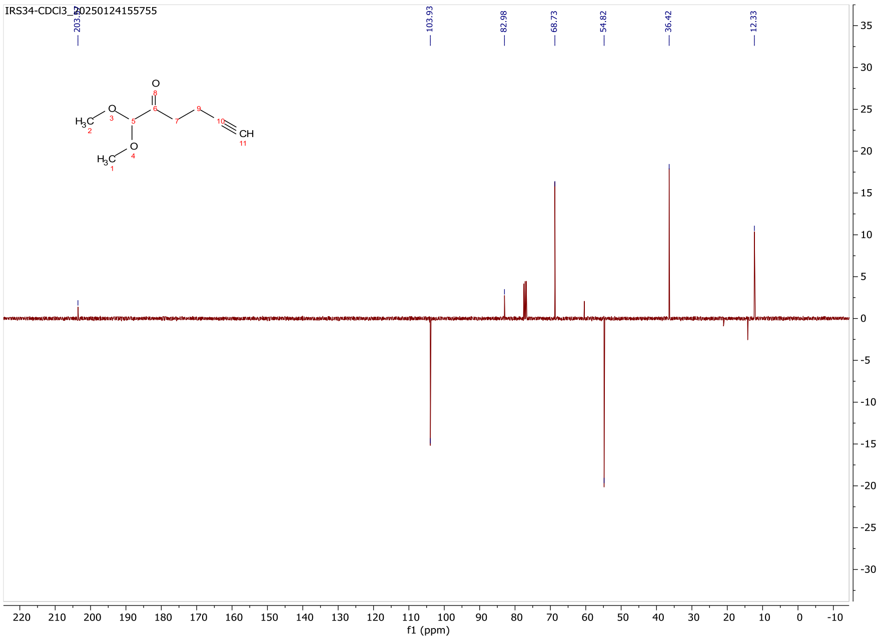

^{1}H NMR of compound 7

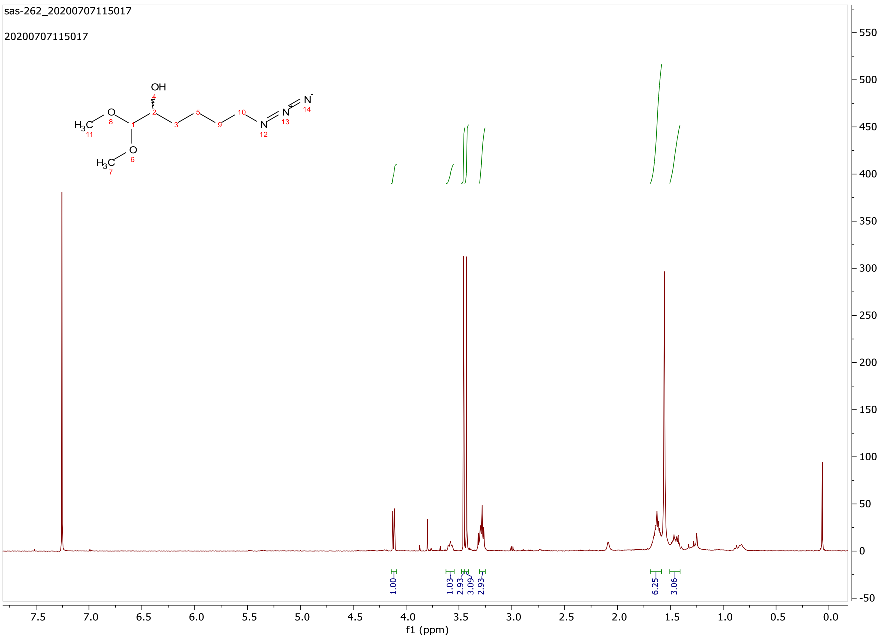

^{13}C NMR of compound 7

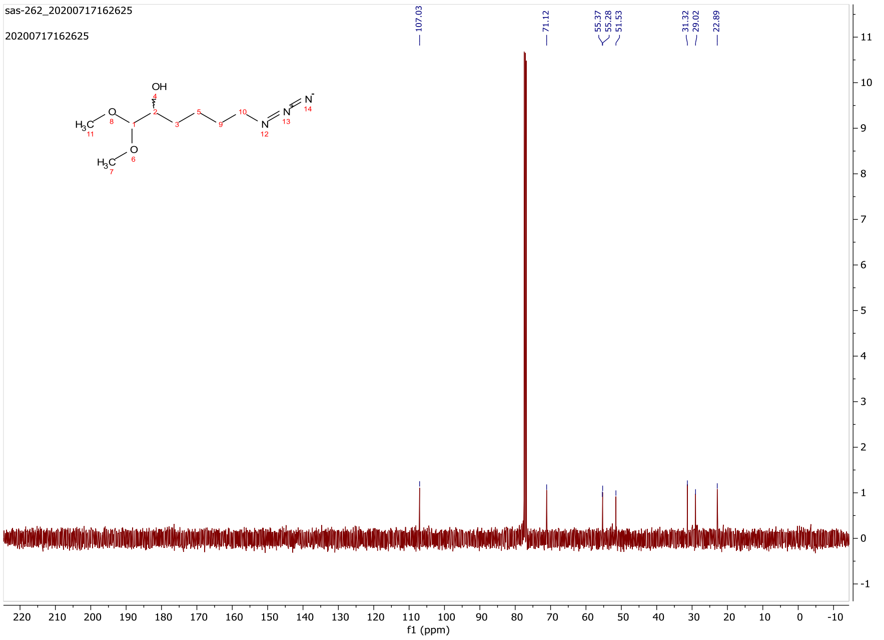

COSY of compound 7

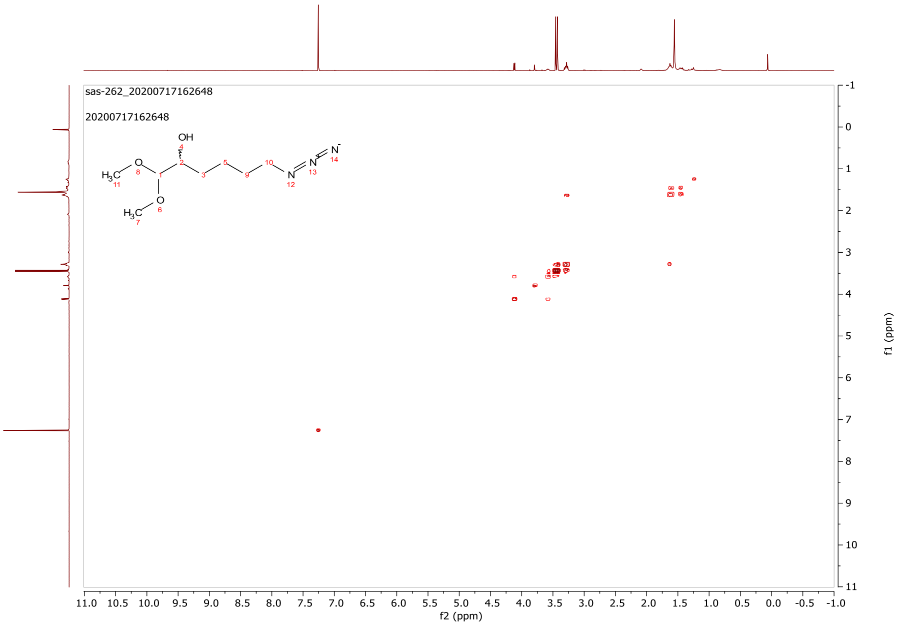

HSQC of compound 7

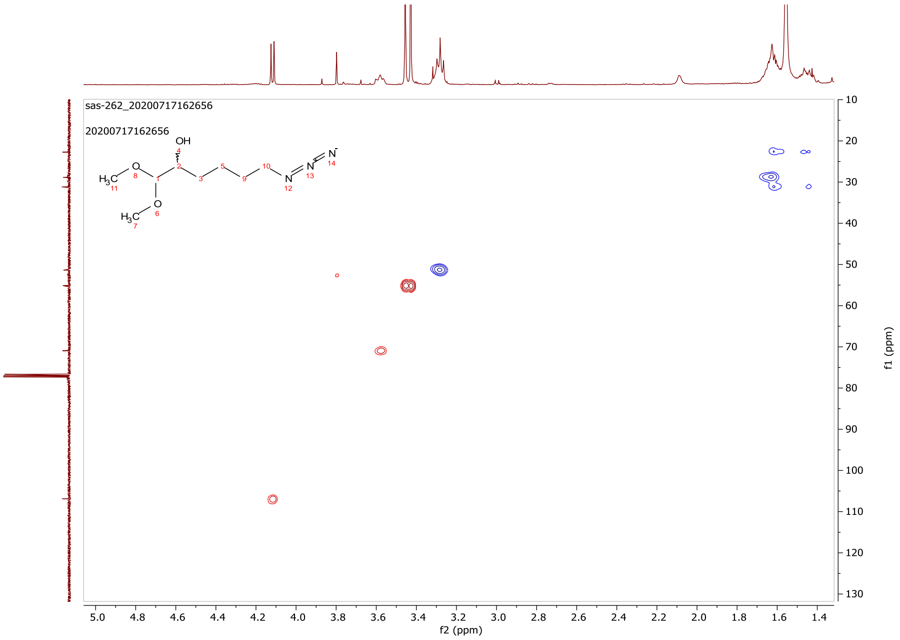

^{1}H NMR of AzBGO-DMA

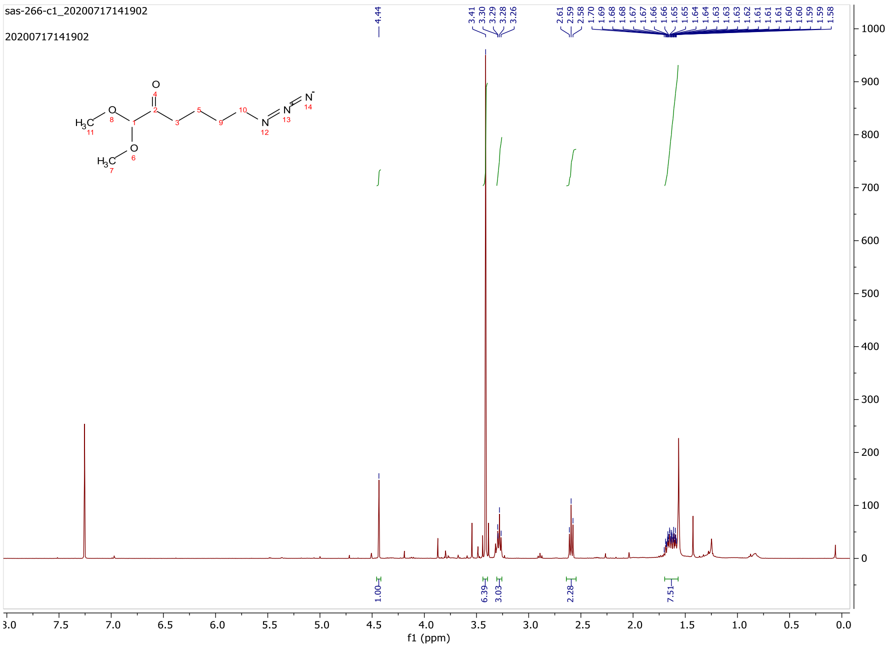

^{13}C NMR of AzBGO-DMA

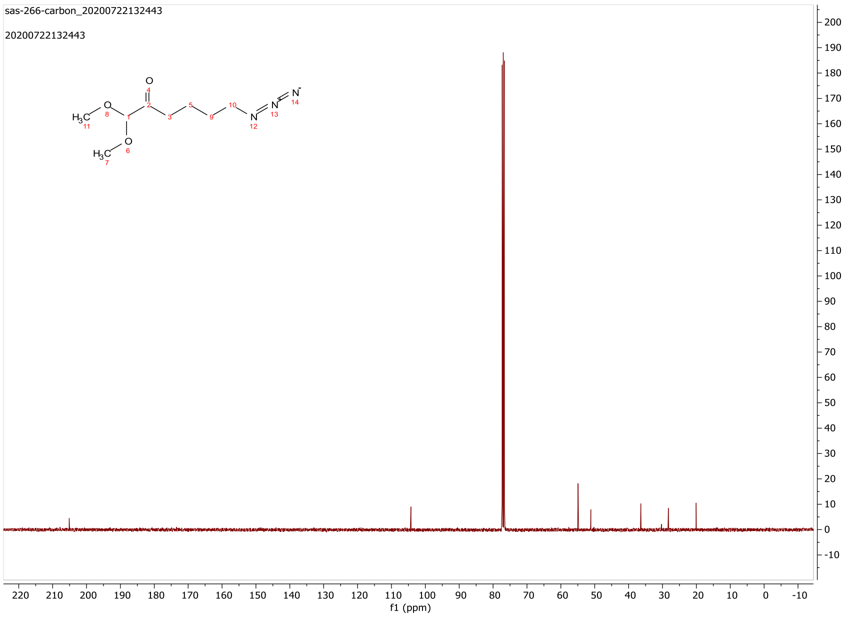

COSY of AzBGO-DMA

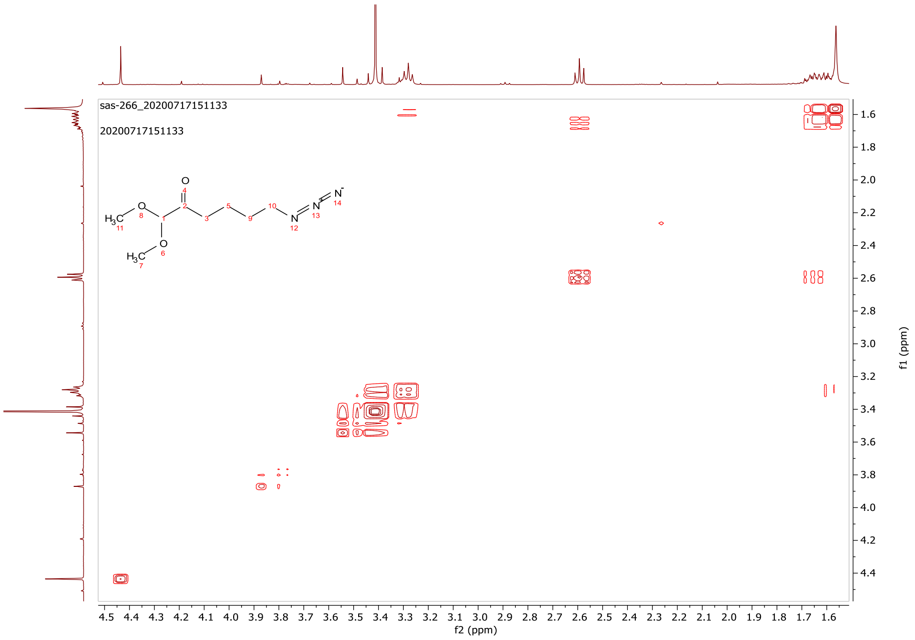

HSQC of AzBGO-DMA

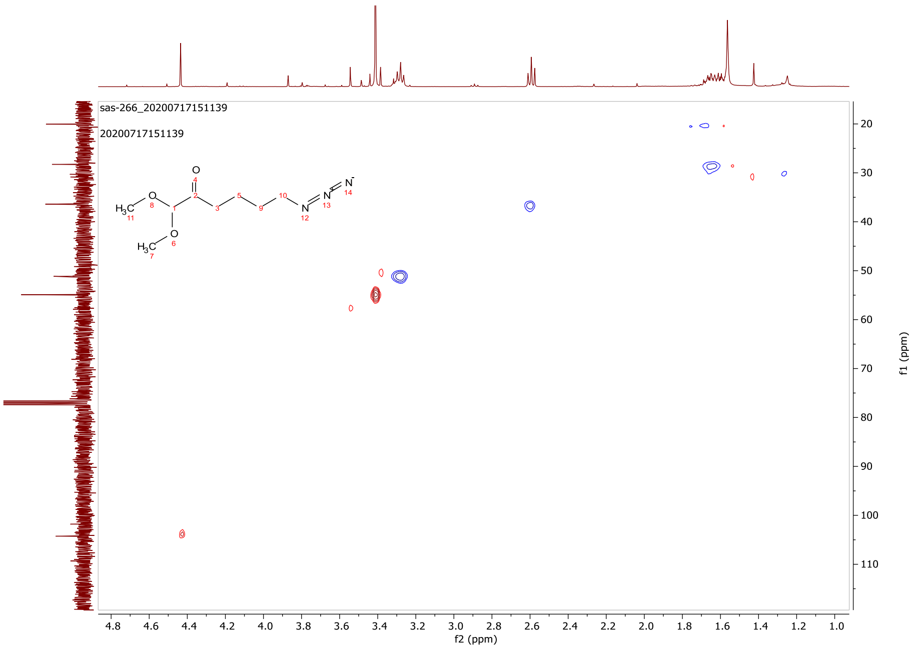

^{1}H NMR of 3-(4,5-dimethoxy-2-nitrophenyl)-3-hydroxy-2,2-dimethylpropanal (intermediate 10)

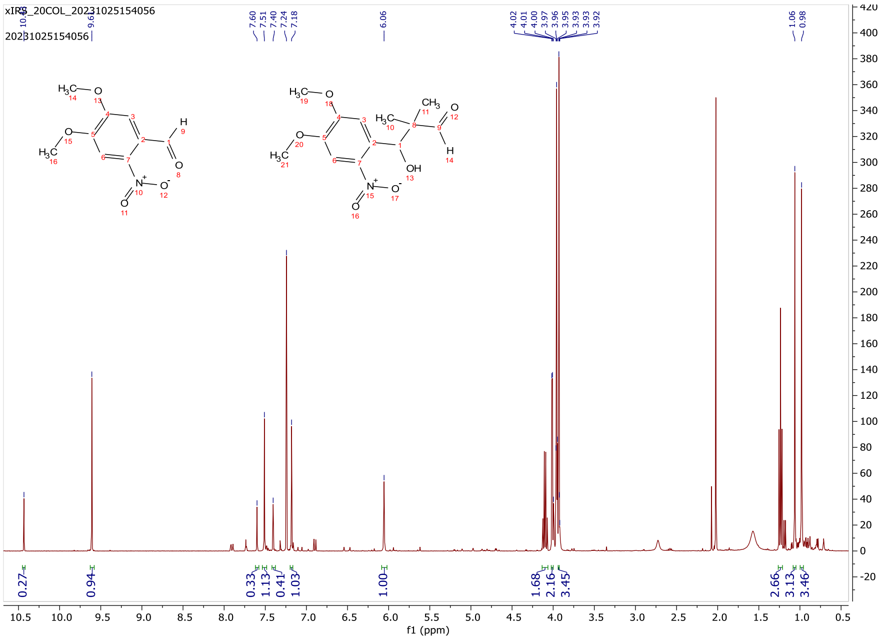

^{1}H NMR of 1-(4,5-dimethoxy-2-nitrophenyl)-2,2-dimethylpropane-1,3-diol (PC2)

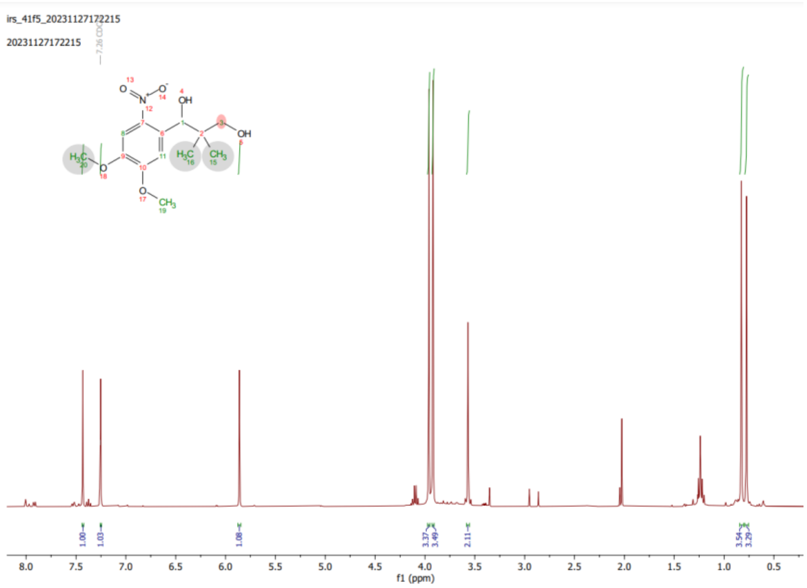

HSQC NMR

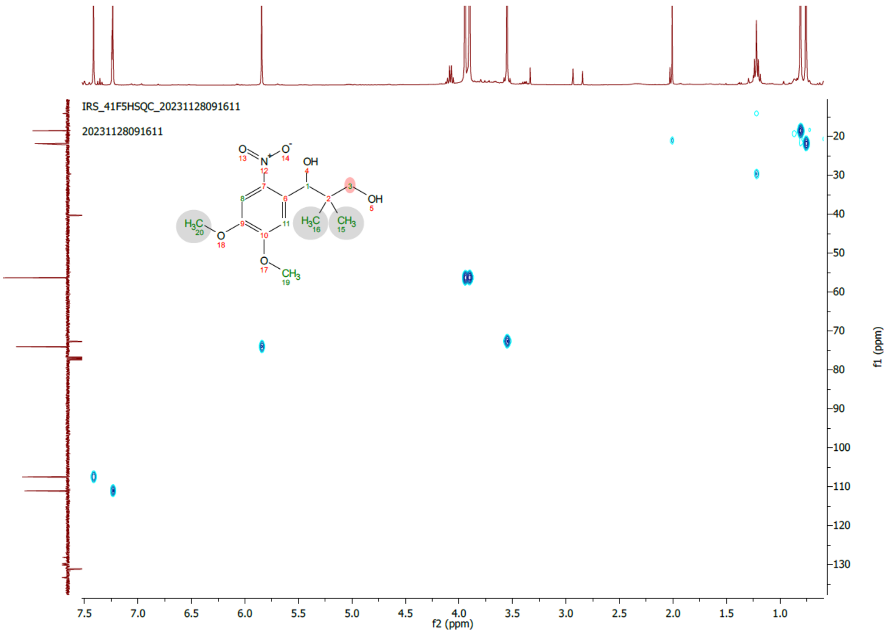

^{13}C NMR

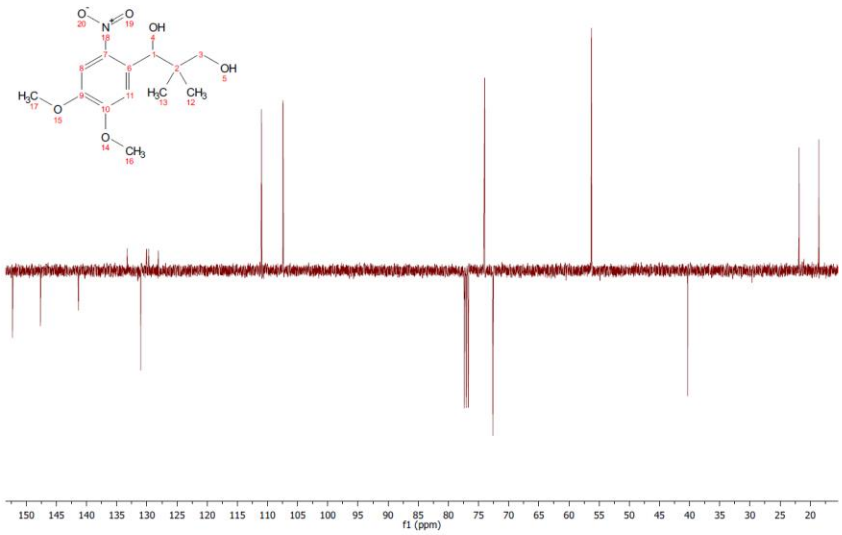

^{1}H-NMR of AzBGO-PC1 alcohol intermediate (first isomer)

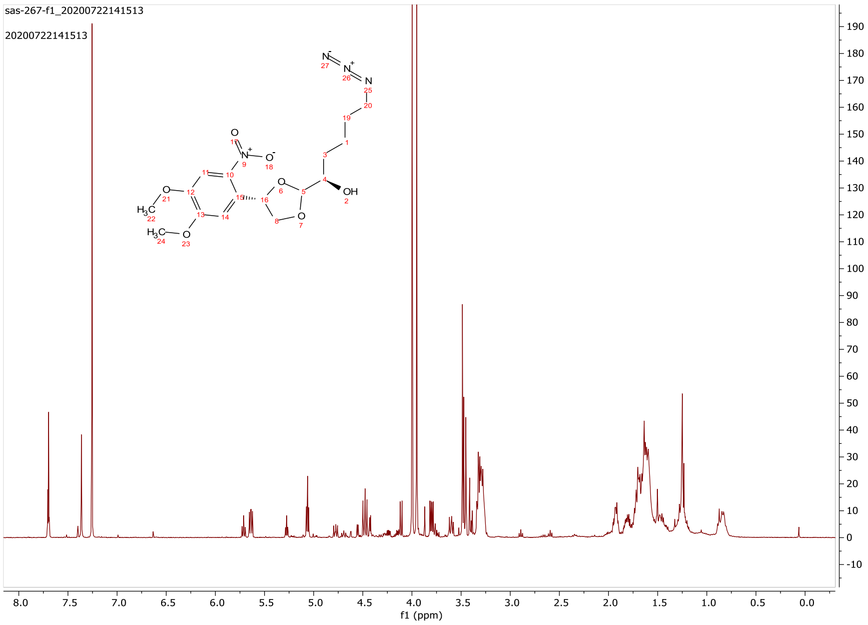

^{13}C-NMR of AzBGO-PC1 alcohol intermediate (first isomer)

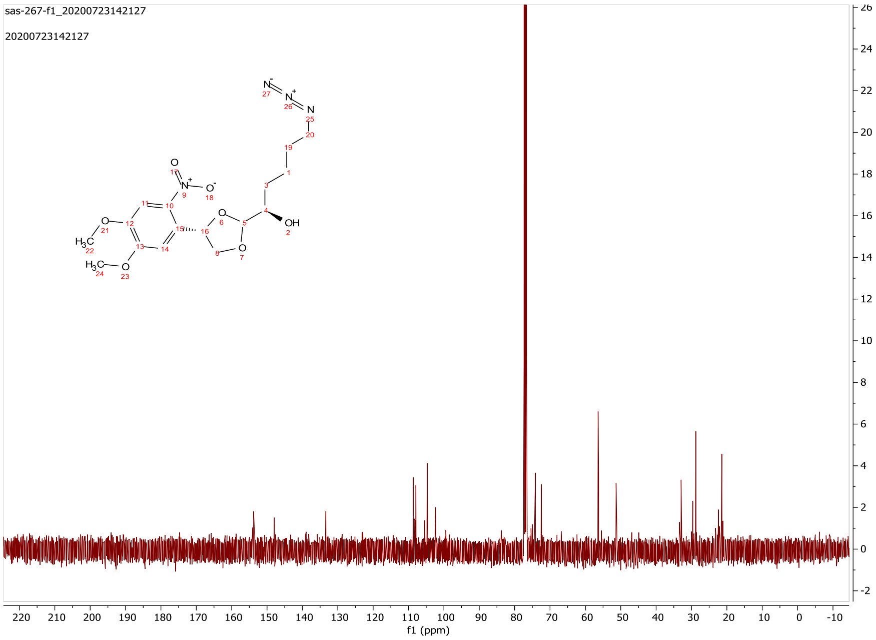

HSQC of AzBGO-PC1 alcohol intermediate (first isomer)

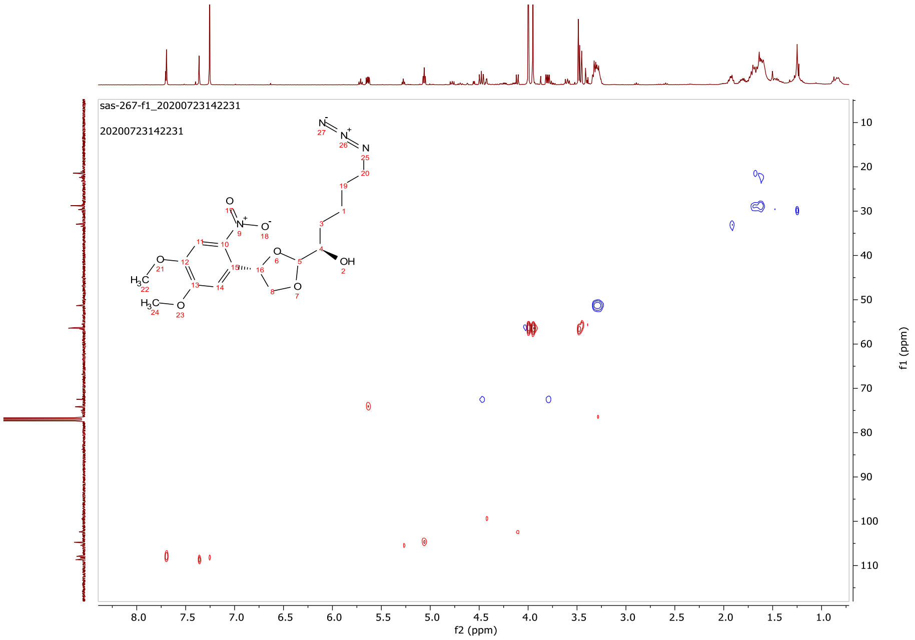

^{1}H-NMR of AzBGO-PC1 alcohol intermediate (second isomer)

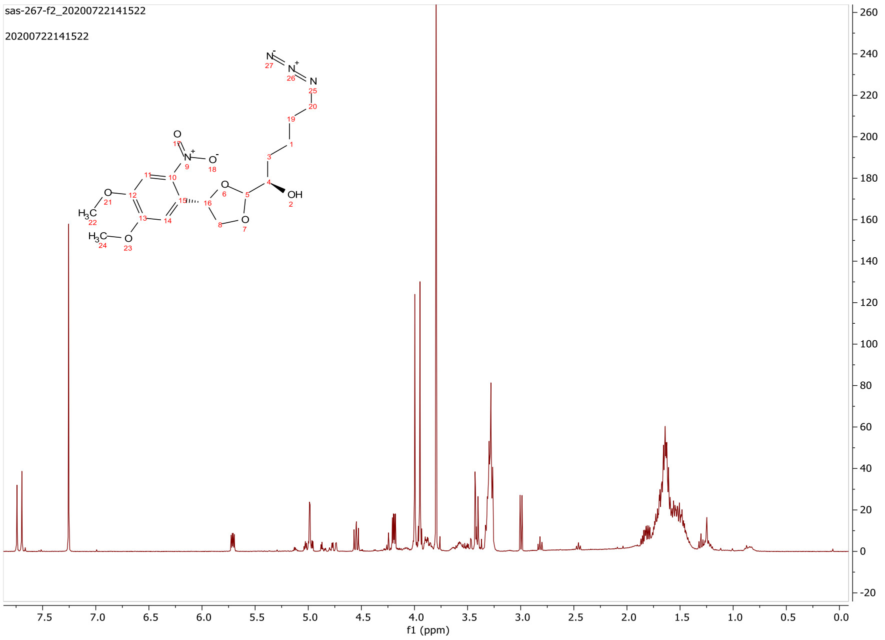

^{13}C-NMR of AzBGO-PC1 alcohol intermediate (second isomer)

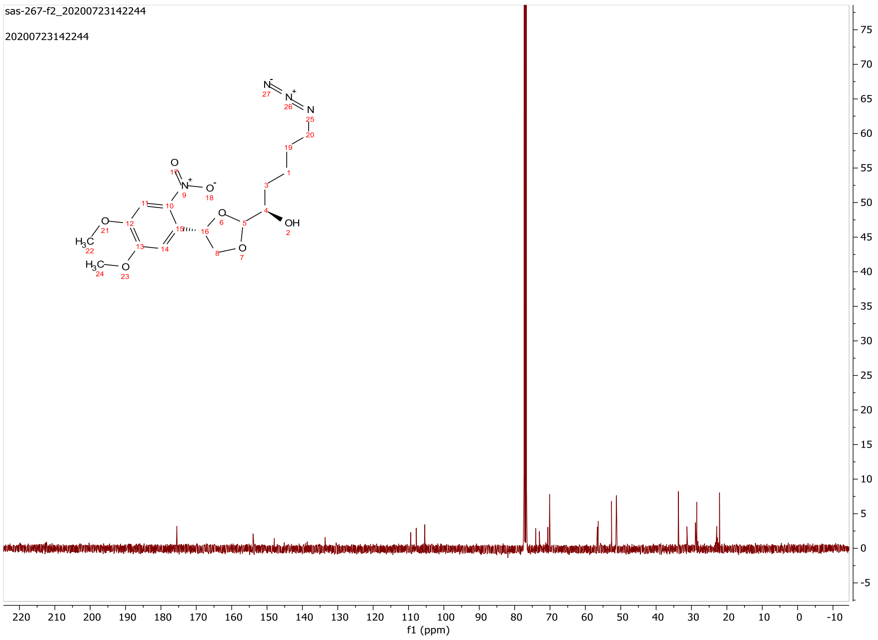

HSQC of AzBGO-PC1 alcohol intermediate (second isomer)

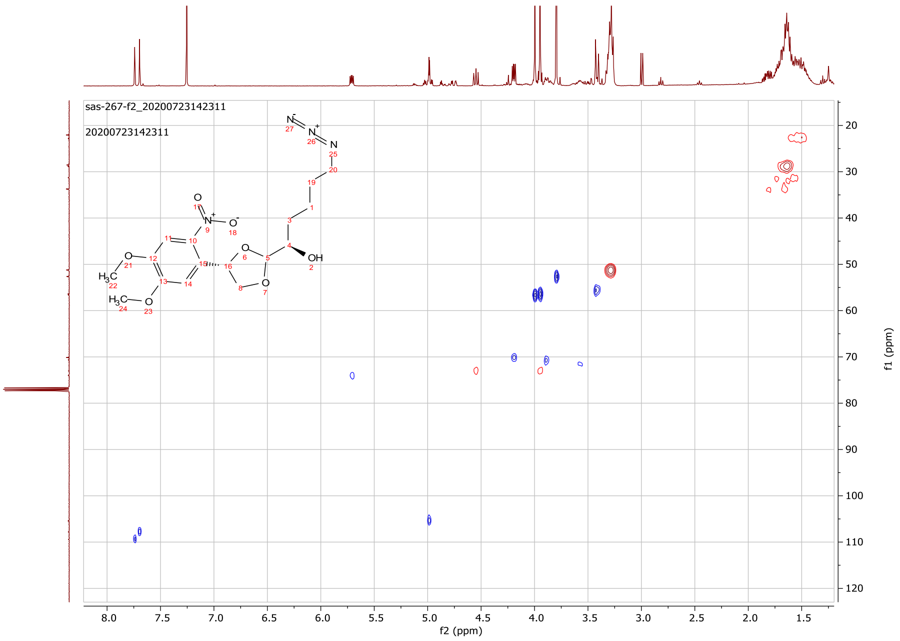

^1H NMR of AzBGO-PC1

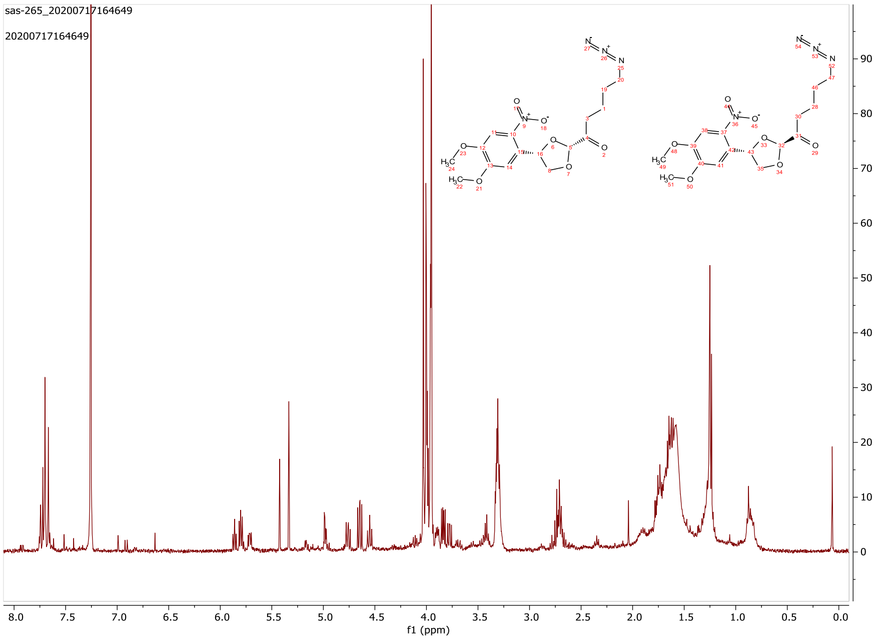

^{13}C NMR of AzBGO-PC1

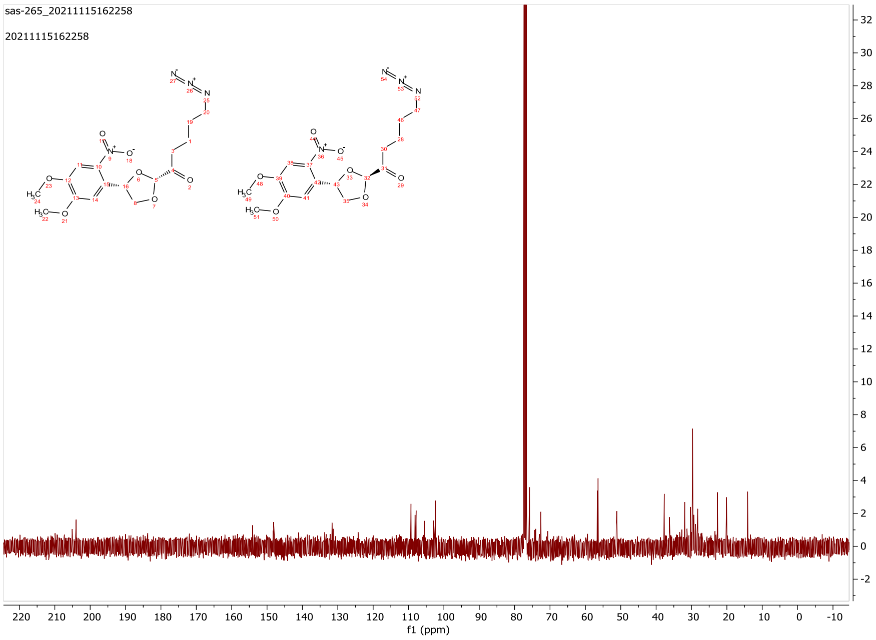

^{1}H NMR of 1,1-dimethoxyhex-5-yn-2-ol 11

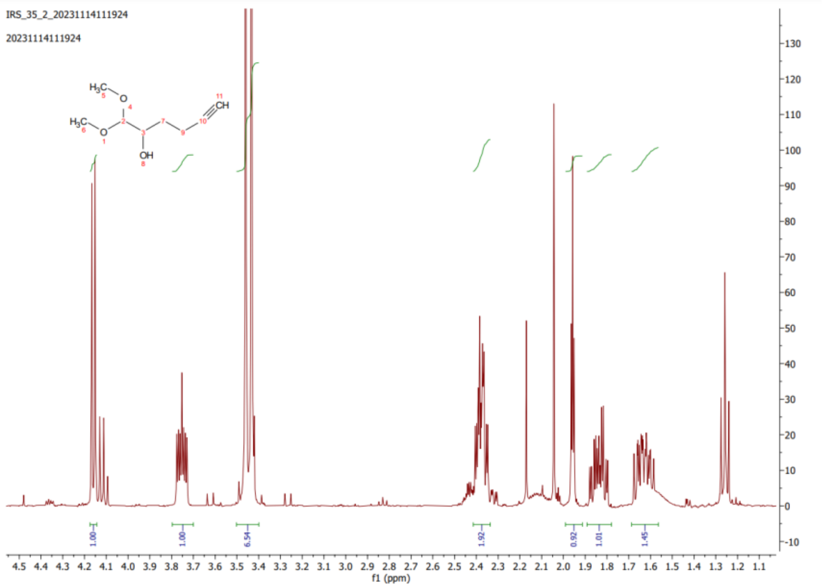

^{1}H NMR of the upper fraction the alcohol intermediate of AlkMGO-PC1

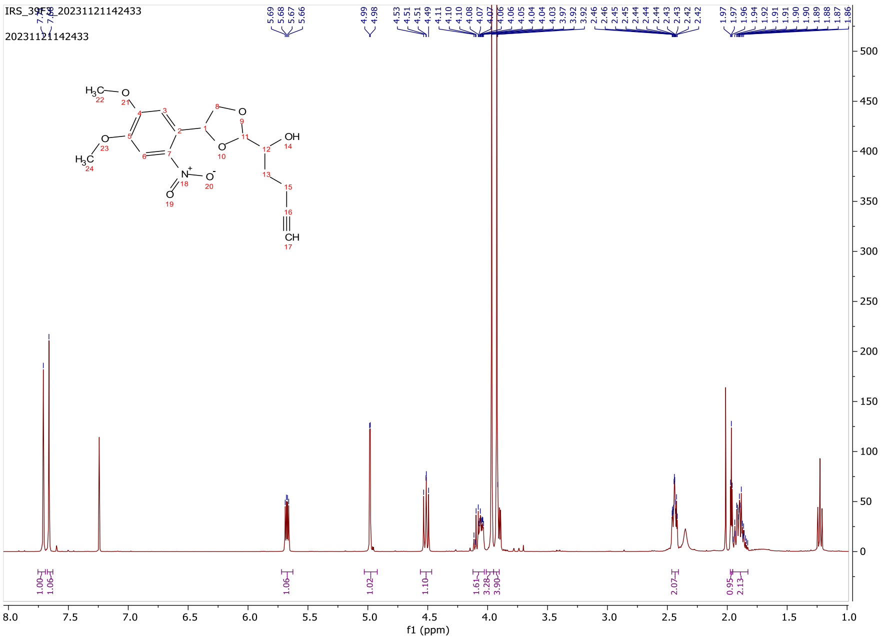

^{1}H NMR of the lower fraction the alcohol intermediate

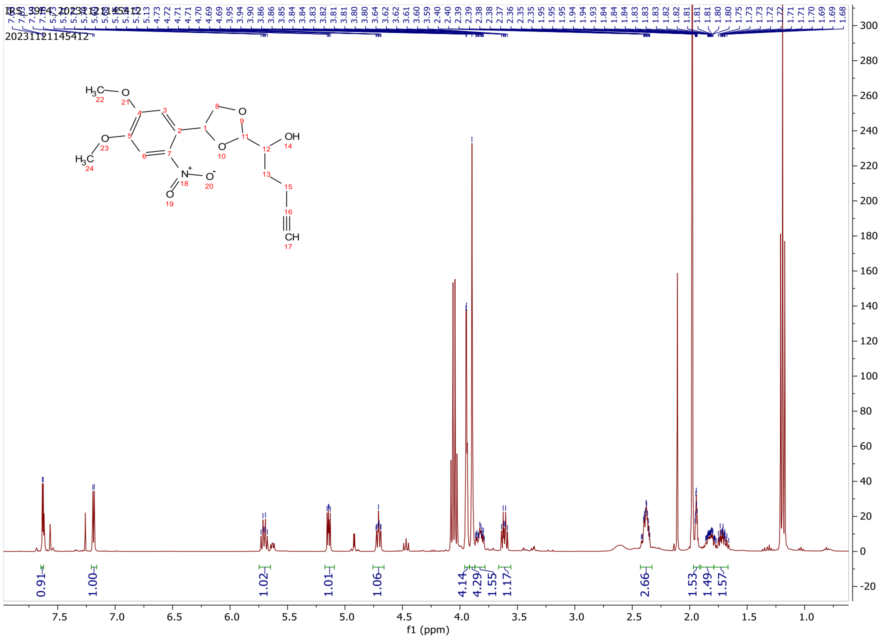

^{1}H NMR of AlkMGO-PC1 cis (obtained after oxidizing the upper fraction)

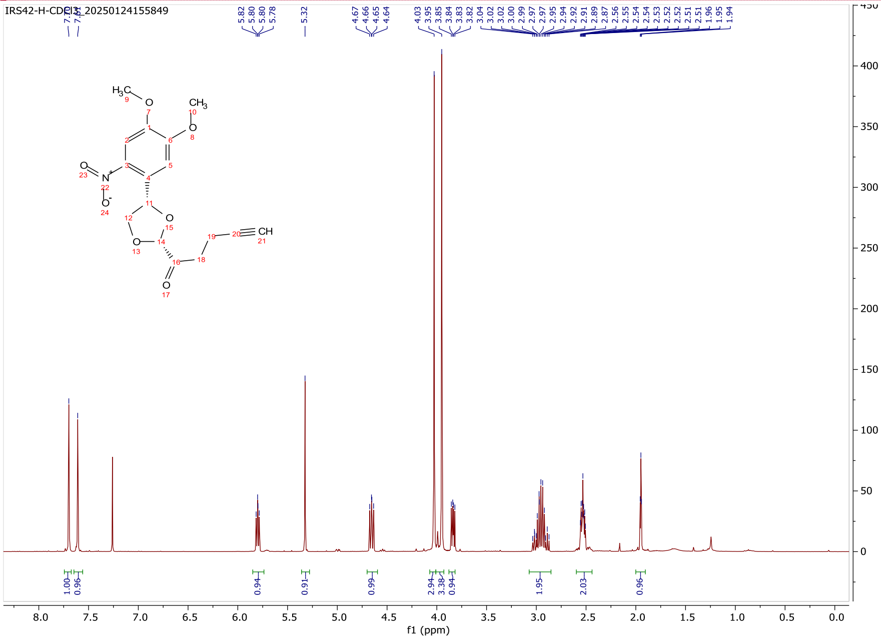

13C-NMR of AlkMGO-PC1 cis

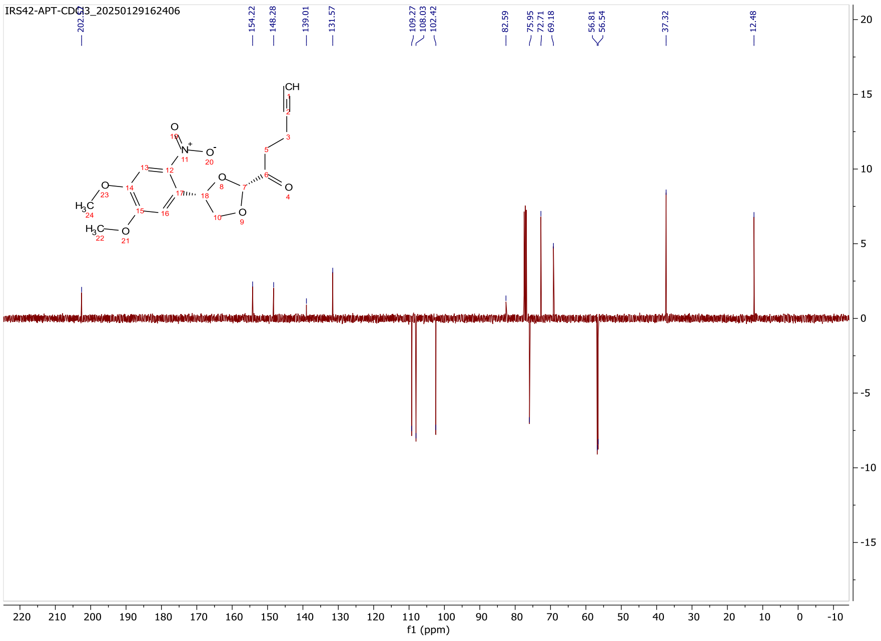

Formatted: Font color: Auto

Formatted: Font color: Auto

HSQC of AlkMGO-PC1 cis

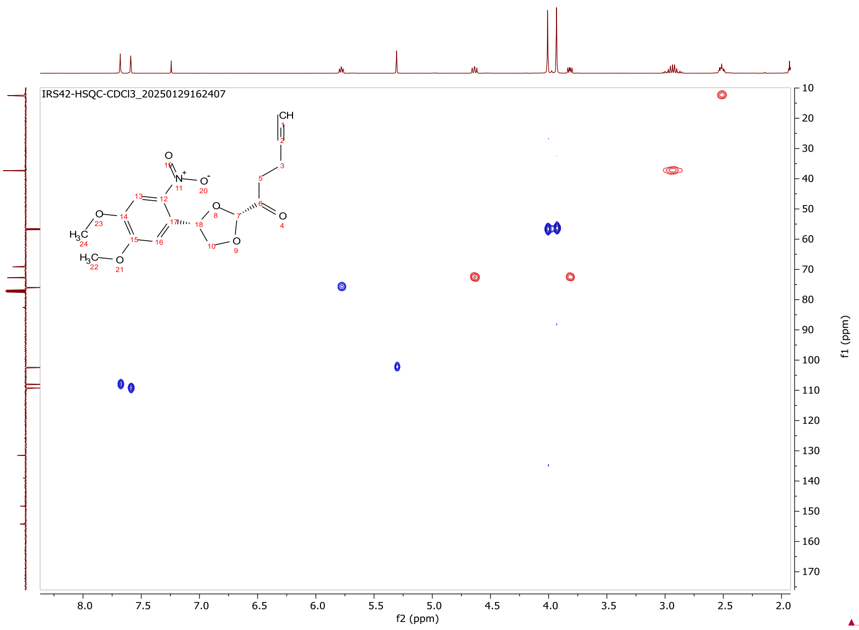

2D-NOESY of AlkMGO-PC1 cis

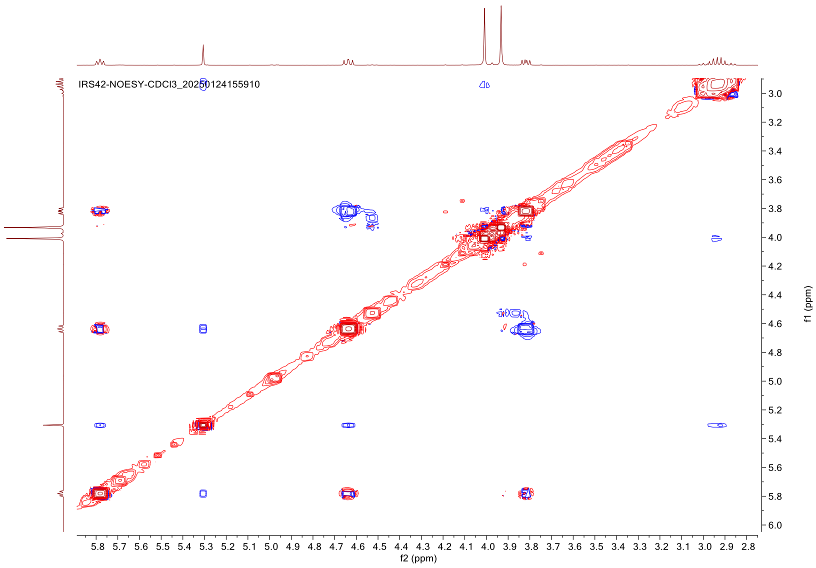

Coupling is observed between the acetal and the benzylic CH as well as the homobenzylic CH of the photocage.

Formatted: Font color: Auto

Formatted: Font color: Auto

Formatted: Font color: Auto

^{1}H NMR AlkMGO-PC1 trans (obtained after oxidizing the lower fraction)

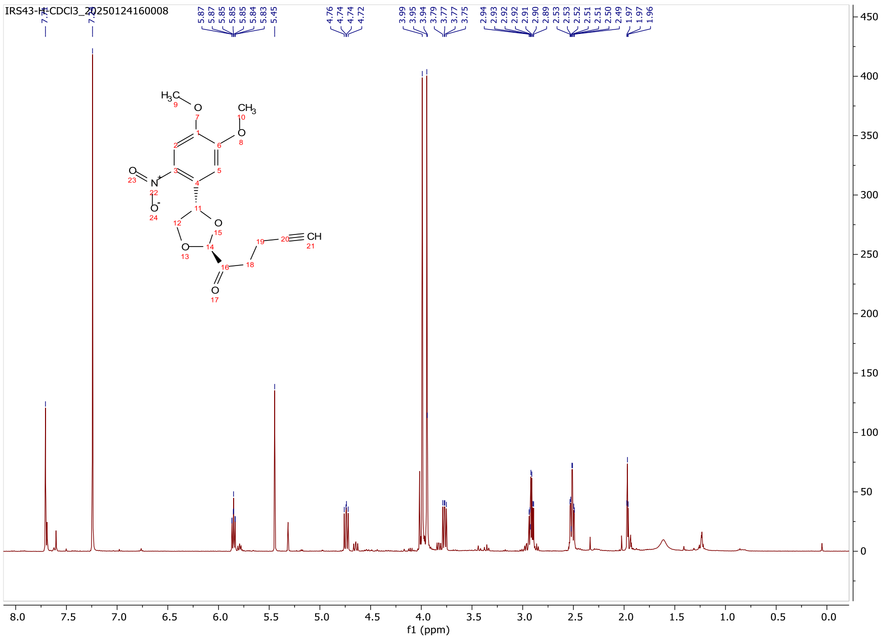

^{13}C NMR AlkMGO-PC1 trans (obtained after oxidizing the lower fraction)

Formatted: Font color: Auto

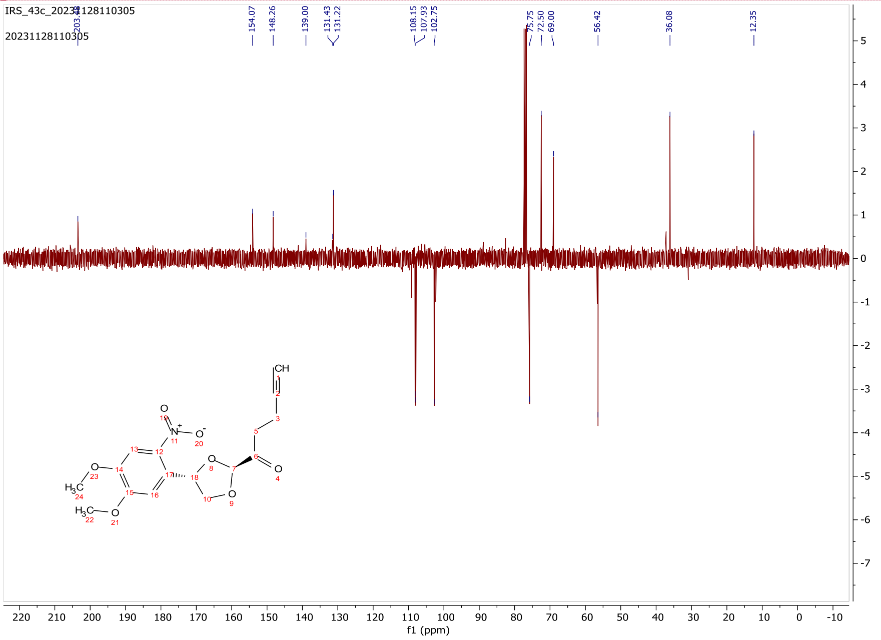

HSQC NMR

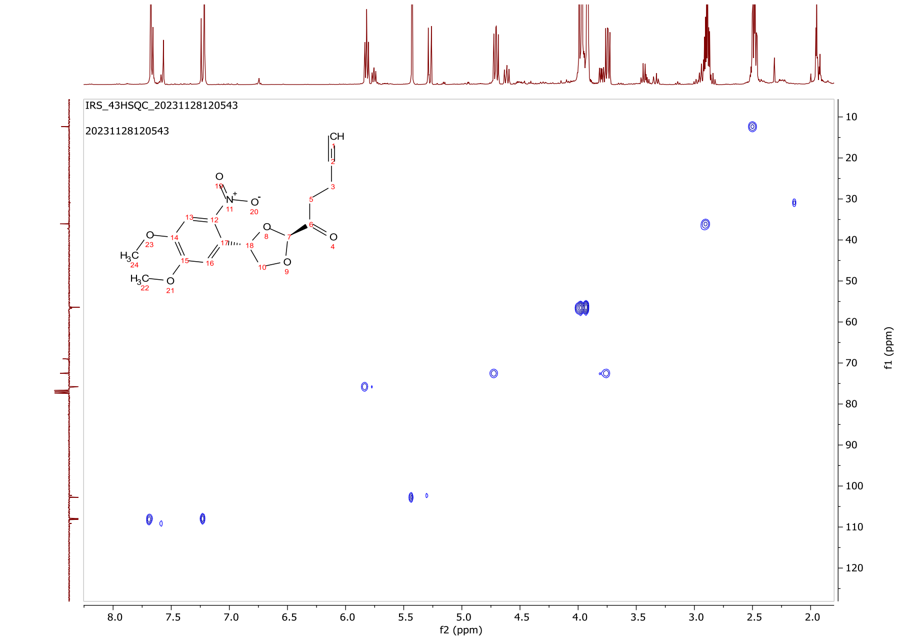

2D-NOESY of AlkMGO-PC1 trans

Formatted: Font color: Auto

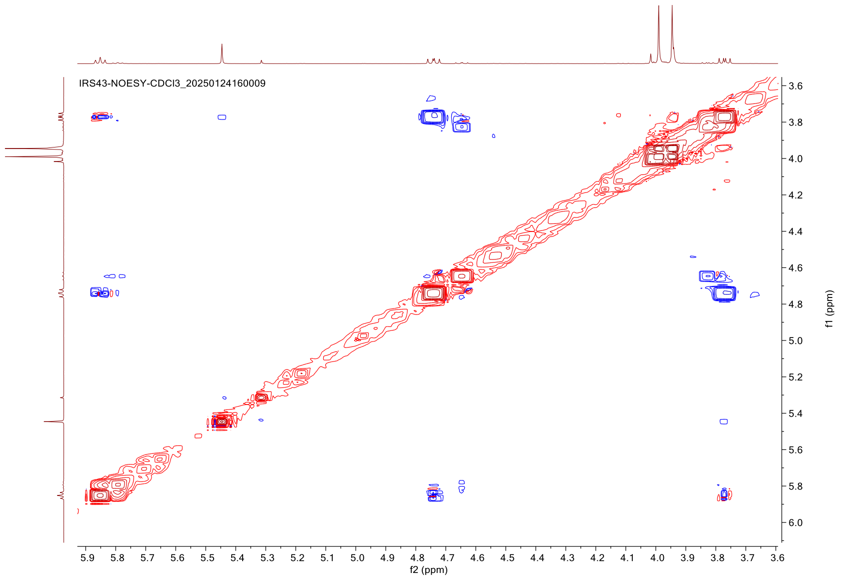

No coupling is observed between the acetal and the benzylic CH of the photocage.

Formatted: Font color: Auto

^{1}H NMR of AlkMGO-PC2 prepared via direct transacetalization

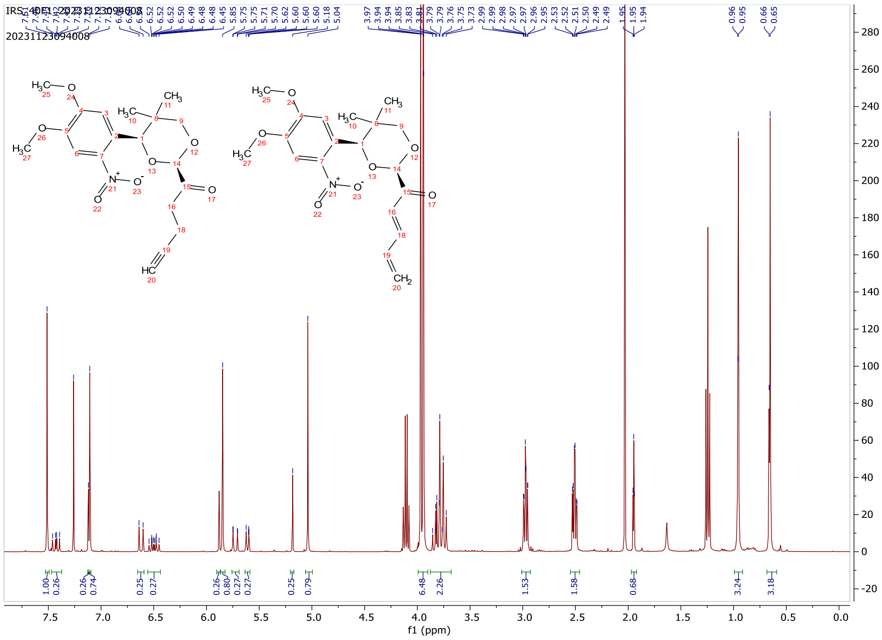

^{13}C NMR of AlkMGO-PC2 prepared via direct transacetalization

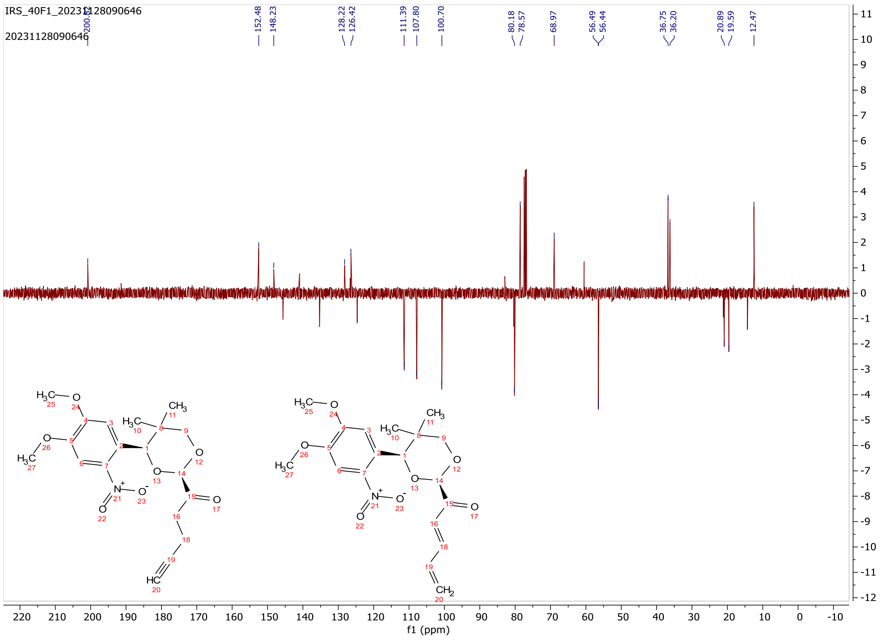

AlkMGO-PC2 prepared via alcohol intermediate

^{1}H NMR of AlkMGO-PC2 of the alcohol intermediate

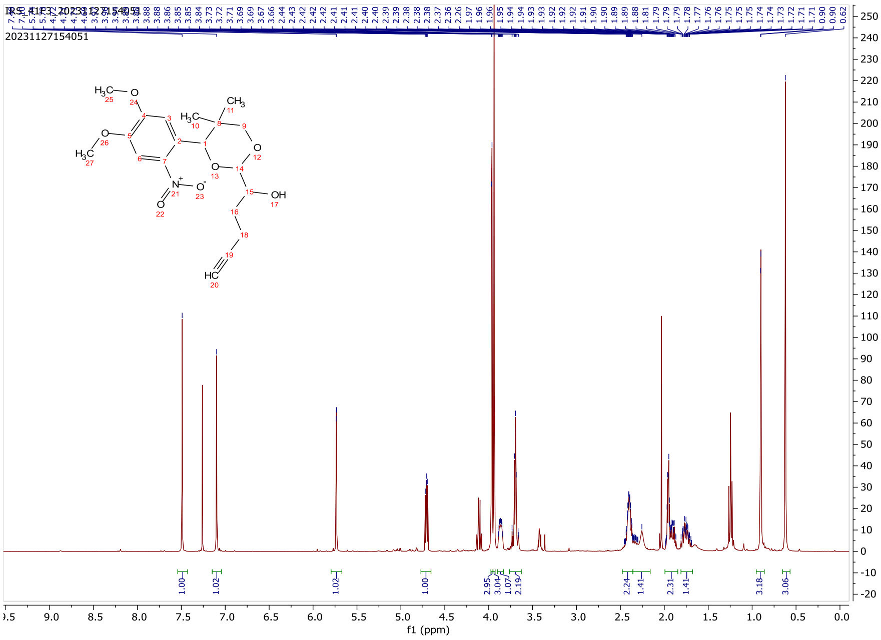

^{1}H NMR of AlkMGO-PC2 obtained after oxidation

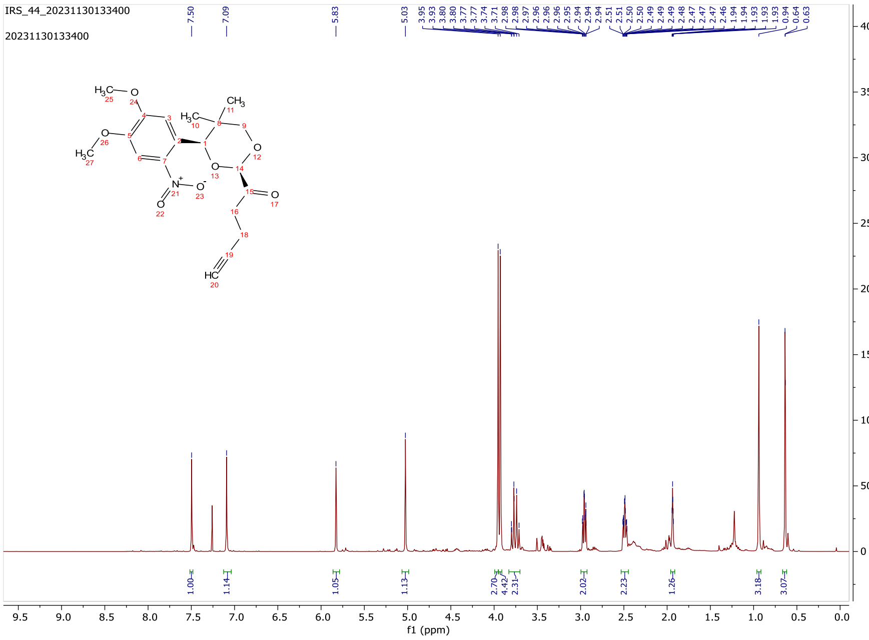

^{13}C NMR of AlkMGO-PC2 obtained after oxidation

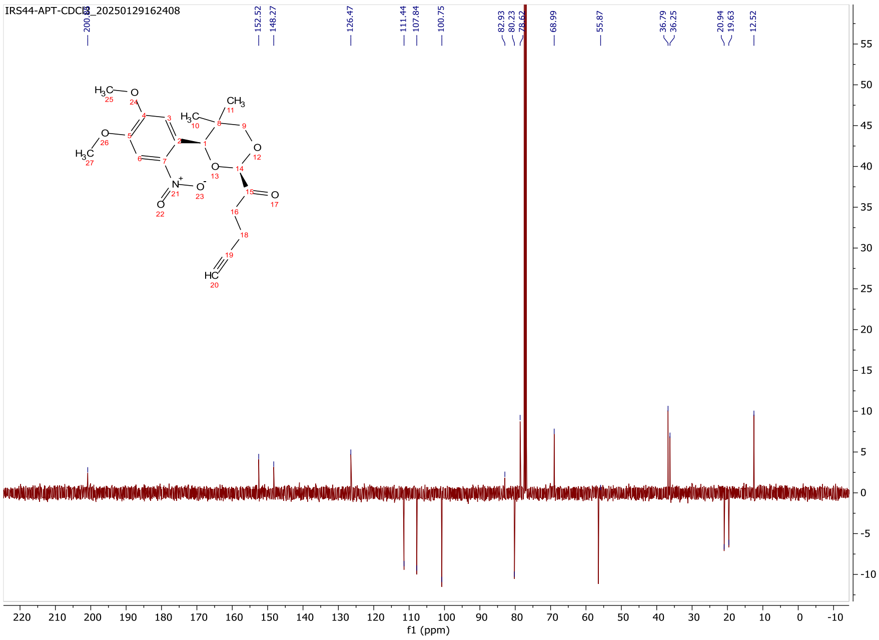

HSQC of AlkMGO-PC2 obtained after oxidation

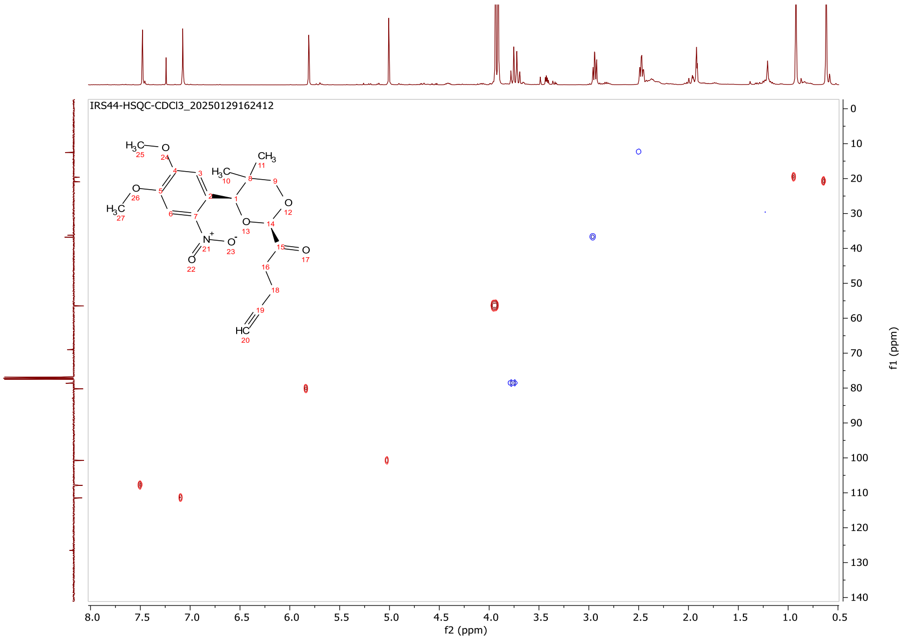

1D-NOESY of AlkMGO-PC2 (excitation of the acetal CH reveals a coupling with the benzylic CH)

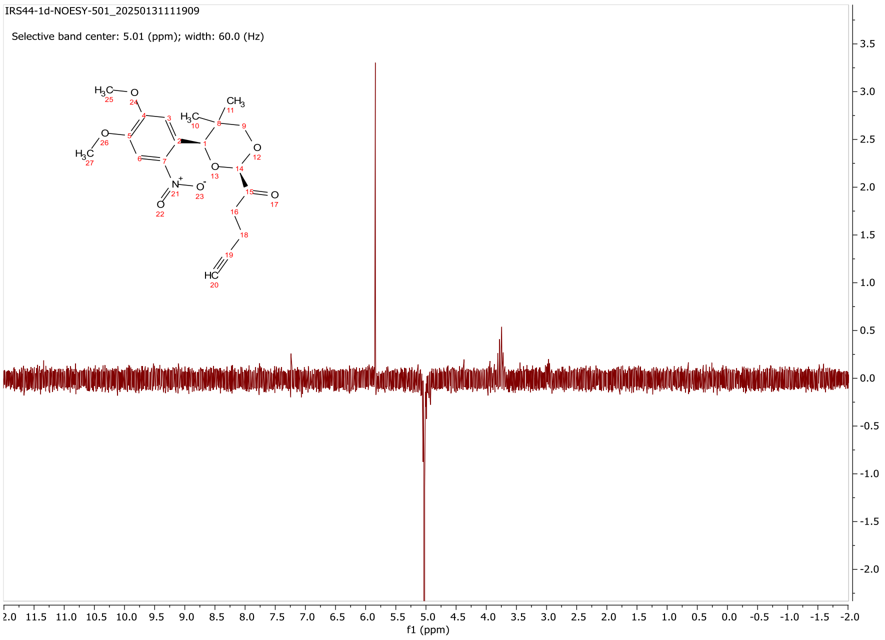

Supplement: Supplementary file 1 — Supplementary Material [file CBIC-26-e202500275-s001.pdf]
